# A framework for modelling desert locust population dynamics and large-scale dispersal

**DOI:** 10.1371/journal.pcbi.1012562

**Published:** 2024-12-19

**Authors:** Renata Retkute, William Thurston, Keith Cressman, Christopher A. Gilligan

**Affiliations:** 1 Epidemiology and Modelling Group, Department of Plant Sciences, University of Cambridge, Downing Street, Cambridge, United Kingdom; 2 Met Office, Fitzroy Road, Exeter, United Kingdom; 3 Food and Agriculture Organization of the United Nations, Viale delle Terme di Caracalla, Rome, Italy; Center for Advanced Systems Understanding (CASUS), GERMANY

## Abstract

There is an urgent need for mathematical models that can be used to inform the deployment of surveillance, early warning and management systems for transboundary pest invasions. This is especially important for desert locust, one of the most dangerous migratory pests for smallholder farmers. During periods of desert locust upsurges and plagues, gregarious adult locusts form into swarms that are capable of long-range dispersal. Here we introduce a novel integrated modelling framework for use in predicting gregarious locust populations. The framework integrates the selection of breeding sites, maturation through egg, hopper and adult stages and swarm dispersal in search of areas suitable for feeding and breeding. Using a combination of concepts from epidemiological modelling, weather and environment data, together with an atmospheric transport model for swarm movement we provide a tool to forecast short- and long-term swarm movements. A principal aim of the framework is to provide a practical starting point for use in the next upsurge.

## Author summary

There is a critical need for tools that can help manage and predict the spread of transboundary pests, especially the desert locust, one of the most destructive pests for smallholder farmers and pastoralists. During upsurges, desert locusts form massive swarms that can travel long distances, causing severe damage to crops and natural vegetation. To address this, we have developed an integrated modelling framework designed to predict the behaviour of these migratory locust populations. The framework takes into account the selection of breeding sites, the life cycle of the locust (from egg to adult), and the movement of swarms as they search for food and breeding grounds. By combining techniques from epidemiological modelling, weather forecasting, environmental data, and atmospheric models, the tool is designed to forecast locust swarm movements in both the short and long term. The ultimate goal of the framework is to provide a practical resource that can be used to prepare for and respond to future locust upsurges.

## Introduction

Desert locust (DL) (*Schistocerca gregaria*) is one of the most dangerous migratory pests for agricultural and rangeland production [1], making control a priority for food security in many regions [2]. The period 2019-2021 was marked by one of the largest DL upsurges over two decades, which saw DL invasion extending from Kenya to India [3, 4]. Mathematical models have previously been developed and used to assess particular aspects of locust dynamics. These include: collective motion of gregarious locusts [5–8], longitudinal flight dynamics [9, 10], foraging behaviour [11, 12], phase transitions between solitarious and gregarious forms [13–16], forecasting gregarization areas [17], climate change role on gregarization [18], population dynamics [19–21], and influence on crop production [22, 23]. Recently, data analytics methods, such as regression and machine learning, have been applied to predict breeding areas of DL [24–28], potential habitat areas of DL [29–32], presence of DL [33–43], the incidence of locust upsurges [44], and migratory routes of locust swarms [45]. They have also been applied to detect DL impact on vegetation senescence [46], cropland damage [47] and socio-economic implications [48]. Major challenges remain in spatiotemporal forecasting of DL, in particular to predict the long–range movements of swarms. The dispersal ability of swarms is a key element in assessing the risks to crop and pasture land and in optimising the management of DL [49, 50].

The current practice for predicting swarm movement relies heavily on expert opinion to integrate data from surveillance, past knowledge of DL behaviour, current and predicted wind movements. Since its inception by FAO approximately 50 years ago, the DL early warning system has been successful in reducing the severity and impacts of DL upsurges during that period [51–53]. New technologies including computational modelling, remote sensing and crowd-sourcing are being progressively incorporated into the FAO early warning system for DL. During the recent 2019-21 DL upsurge, near real–time outputs from weather-driven atmospheric transport models, adapted to model DL swarm dispersal, were used to improve predictions of areas at risk. These innovations included twice-weekly forecasts of likely swarm movements using NAME (the Numerical Atmospheric-dispersion Modelling Environment, [54]) provided by the UK Met Office [55] and a web–based app for forward or backward simulation of swarm movement based on air trajectories from the Hybrid Single-Particle Lagrangian Integrated Trajectory (HYSPLIT) model, provided by the US National Oceanic and Atmospheric Administration (NOAA) [56]). The availability of atmospheric transport models for wind-assisted dispersal of DL provides a valuable tool for analysis (using historic weather data) and prediction (using forecast weather and surveillance data) of long-distance swarm movements. Atmospheric transport models have previously been used to analyse the migratory trajectories of insects, including the annual migration of painted lady butterflies [57], seasonal migration of fall armyworm moths [58] and midge flight activity [59]. The NAME model also underpins a successful, near real-time, weather-driven, early warning system for long-range dispersal of wheat rust spores [60] initially deployed in Ethiopia [61] and now extended to South Asia [62].

In the current paper, we introduce and test a spatially explicit and stochastic compartmental framework that follows gregarious locust populations through egg, hopper and adult stages as they develop, mature, and disperse in search of areas suitable for feeding and breeding. We consider a domain extending across five sub-Saharan countries (Kenya, Ethiopia, Somalia, Eritrea and Djibouti) that were affected by the 2019-21 DL upsurge and we focus on gregarious populations because these pose the largest threat to livelihoods [63]. The framework builds on epidemiological modelling concepts [64] and an atmospheric transport model for swarm movement. The framework takes account of surveillance data for input, as well as dynamically changing conditions that affect the availability of vegetation for locust feeding. It is designed for analysis and prediction at country-wide and regional scales, with a spatial resolution of 1 km × 1 km.

The principal aim of the work is to provide a novel integrated modelling framework that can be used as a practical starting point for use in the next upsurge to inform surveillance and control. Specifically we address the following topics:

- construction of a modelling framework that integrates DL breeding, development through egg, hopper and adult stages, feeding and swarm migration;
- characterisation of breeding sites using a combination of site-specific static and dynamic variables to predict egg-laying, hopper and adult development leading to DL swarming;
- incorporation of weather-driven models for wind trajectories to predict daily pathways of swarm migration;
- use of remote-sensed data to predict the duration of swarm feeding at a single location given the state and availability of vegetation for feeding;
- use of the modelling framework to analyse and predict: (i) long–distance, seasonal movement of swarms, e.g. to reach Kenya from breeding sites in Somalia; (ii) short–term forecasting of swarm movements.

We also address the question of how to assess flight direction and likely origin and landing sites for swarms observed in flight.

## Materials and methods

### Modelling framework overview

**Fig 1:**
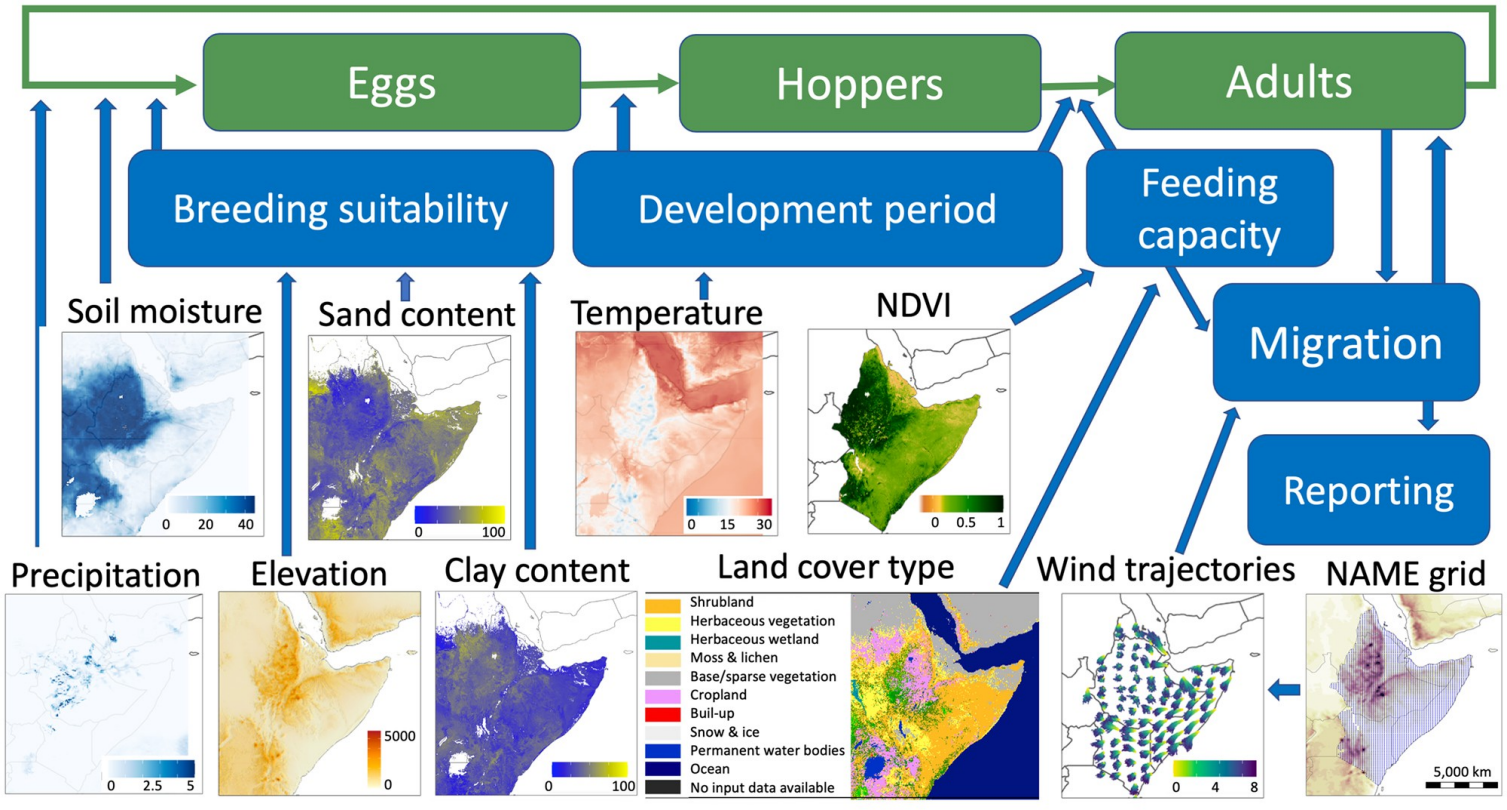
Conceptual diagram of the spatially explicit model that integrates the life cycle of desert locust, climate variables, remote sensing data, and wind–assisted migration. Life cycle compartments are *Eggs*, *Hoppers* and *Adults* (green boxes), sub-models are *Breed suitability*, *Development period*, *Feeding capacity*, *Migration* and *Reporting* (blue boxes). The model is restricted to the dynamics of gregarious locusts. The base layer of the map is sourced from Natural Earth (https://www.naturalearthdata.com).

We assumed that DL populations can be in one of three non-overlapping life-stages, also called compartments: eggs, hoppers (nymphs) and adults (Fig 1). Each compartment has different ecological requirements and responds differently to environmental conditions, such as climate, soil and vegetation [65]. The model operates on a gridded landscape at a resolution of 1 km × 1 km over one day time steps and represents three main processes: (i) the biology and behaviour of DL; (ii) meteorological and environmental conditions; and (iii) wind–assisted migration of DL swarms. We restricted the model to dynamics of gregarious locusts, as these pose the largest threat to livelihoods [63]. As the model focuses on gregarious swarm behaviour and movement, we have not accounted for transitions between solitarious and gregarious phases observed in DL.

### Study area

We consider a domain extending across five sub-Saharan countries (Kenya, Ethiopia, Somalia, Eritrea and Djibouti) that were affected by the 2019-21 DL upsurge (Fig A in S1 Appendix). In accordance with our study area, our simulations were restricted to longitudes 30° to 55°E and latitudes 5°S to 20°N.

### Datasets

The data used encompass a diverse range of spatial and temporal resolutions. We chose a spatial resolution of the modelling framework to be 1 km × 1 km (Fig D in S1 Appendix). The framework could be formulated at any required spatial resolution, but we deemed that 1 km was suitable for biological and migratory processes under consideration. Datasets were upscaled or downscaled to the spatial resolution of 1 km. The exception was for the data used to calculate breeding suitability on the Google Earth Engine (GEE) platform [66] (elevation, sand and clay content in the soil), which were used at their native resolution (90-250 m). The GEE platform has built-in capabilities to handle data at different scales.

- **DL surveys.** Surveillance data on desert locust reports were obtained from the FAO Locust Hub website [67]. We extracted all records dated between 1^*st*^ January 2010 and 1^*st*^ July 2021 with locations within the study area. The repository has data assigned to the following classes: ‘Hoppers’, ‘Adults’, ‘Bands’, ‘Swarms’, ‘Ecology’ and ‘Control’. The ‘Ecology’ class contains records on ecological conditions at all visited sites irrespective of DL presence or absence (Fig E in S1 Appendix).
- **Elevation.** The Shuttle Radar Topography Mission (SRTM) digital elevation dataset) [68] was used for elevation. The resolution of the dataset was 90 m (Fig G in S1 Appendix).
- **Sand and clay content in the soil.** Sand content at 5 cm depth (weight percentage of the sand particles (0.05-2 mm) and clay content at 5 cm depth (weight percentage of the clay particles (< 0.0002 mm) were obtained from ISRIC soilgrids dataset) [69, 70]. The resolution of the data was 250 m (Fig H in S1 Appendix).
- **Land cover data.** We extracted data for land cover classification from the Copernicus global map of land cover at 100 m resolution (CLC100) [71]. Land cover maps represent spatial information on different types of the Earth’s surface, e.g. urban areas, sparse vegetation, cultivated vegetation. We downscaled the resolution to 1 km × 1 km (Fig I in S1 Appendix).
- **The normalized difference vegetation index NDVI.** We used 16-day averaged moderate-resolution imaging spectroradiometer (MODIS) data (NASA LP DAAC at the USGS EROS Center Terra Vegetation Indices 16-Day Global MOD13Q1.061) [72]. The data for Kenya, Ethiopia, Somalia, Djibouti and Eritrea and dates in the range between 1^*st*^ January 2019 and 1^*st*^*January*2022 were downloaded from the Google Earth Engine platform [66] at 1 km × 1 km resolution (Fig J in S1 Appendix).
- **Temperature, soil moisture and precipitation.** The UK Met Office Numerical Atmospheric-dispersion Modelling Environment (NAME) was used to model a wide range of atmospheric dispersion events [73]. The predictions were driven using historic analysis meteorology data from the global configuration of the Unified Model (UM) [73]. The UM provides 0-10 cm soil moisture at 3 hourly time intervals on its native horizontal grid of approximately 10 km by 10 km (Fig K in S1 Appendix). We upscaled the resolution to 1 km × 1 km, by assigning each 10 km × 10 km grid value to all 1 km × 1 km cells within the larger cell.
- **Wind trajectories.** The Lagrangian atmospheric dispersion model NAME [54] was used to calculate stochastic trajectories of potential wind-borne DL swarm migrations. Originally developed by the UK Met Office to model the release, transport, dispersion and removal of hazardous material in the atmosphere [54], NAME has subsequently been adapted to model long-distance wind-borne transport of insect vectors of viral disease [59] and fungal spores [60]. NAME wind trajectories were calculated starting from 3860 source locations spaced on a regular 20 km × 20 km grid. For each day between 1^*st*^ September 2019 and 31^*st*^ December 2021 and the 3860 starting locations, 1000 individual trajectories were computed (Fig L in S1 Appendix). Trajectories commenced 2h after local sunrise and terminated 1 h before local sunset [74]. This resulted in the generation of nearly 3.3 billion trajectories over the study period, each providing highly-resolved data (longitude, latitude, height, pressure and temperature) at a temporal resolution of 0.5 h. As wind trajectories follow turbulent atmospheric flows, the height of wind trajectories varied with time and location, but were between 0 and 2000 m above the ground. Realisations of wind trajectories were calculated and stored as Network Common Data Form (NetCDF) files.

### Implementation of the modelling framework

**Fig 2:**
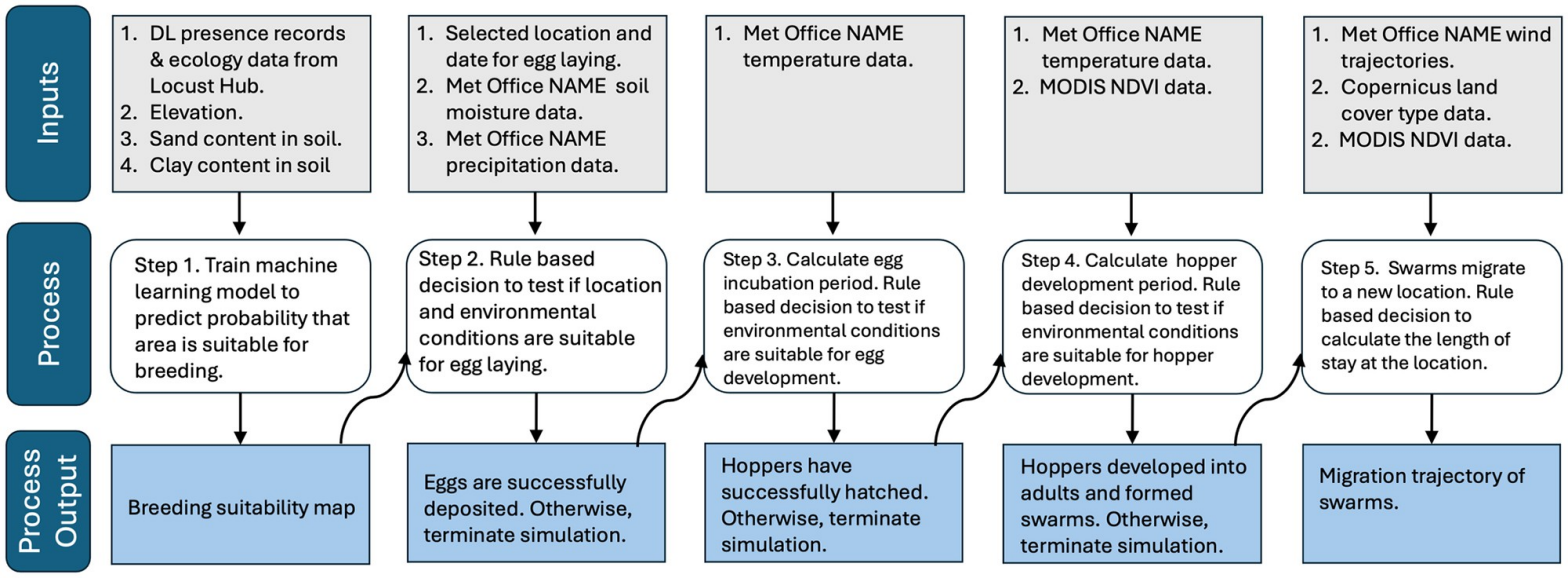
Schematics of the modelling framework.

The steps involved in execution of the framework are shown in Fig 2. The first step in modelling is to produce a breeding suitability map. Identifying breeding areas is important both for monitoring situations for preventive management and for understanding potential DL migratory patterns. The framework simulation starts with choosing a location and date for breeding. Step two involves checking if environmental conditions at the specified location are suitable for egg laying. If eggs are successfully deposited, then the model proceeds to step three. For this, the egg incubation period leading to hopper emergence is calculated based on the temperature at the location. If the egg incubation period is within a biologically viable range, we assume that hoppers have hatched. Step four involves calculating hopper development period leading to adult emergence and testing if conditions in terms of available vegetation, are suitable for DL feeding. The final step is dispersal and feeding of swarms, which involves analysis of wind-assisted dispersal trajectories and testing how long vegetation available on a ground can sustain a swarm at successive landing sites.

### Components of the modelling framework

#### Pre-processing of NDVI

The NDVI is known to be correlated with photosynthetic characteristics of plants [75] and is used to identify vegetation state at breeding and landing sites. As remote sensing data are usually noisy due to cloud cover and other factors [76], we applied a Whittaker smoother for the NDVI time series analysis [77, 78], with the smoothing parameter set to one (Fig M in S1 Appendix).

We calculated NDVI trends prior to the date of interest from which to infer whether there was fresh vegetation available (increasing trend) for feeding or if the resource were diminishing (decreasing trend) at landing sites. The time resolution of the NDVI series is 14 days. The NDVI trend was assumed to be in one of three states: increasing NDVI values, constant NDVI values or decreasing NDVI values. The NDVI trend was estimated in two steps. First, we used the R package *segmented* [79] to calculate break-points of the smoothed NDVI curve during a period of 150 days prior to the date of interest. Then we combined segments that had the same direction of change, i.e. increasing NDVI value or decreasing NDVI value. The last segment before swarm landing date then was used to define the NDVI trend (Fig N in S1 Appendix). To find local peaks in a NDVI profile, we used the R repository ‘findPeaks’ [80]. A peak was defined as a point such that *m* points either side of it have a lower or equal value to the point and was assumed to indicate the onset of fresh vegetation. We set *m* = 2, which corresponds to ± 1 month (Fig O in S1 Appendix).

#### Predicting suitability for breeding

We applied a machine learning approach to combine environmental data with presence/absence reports of hopper/band in order to predict areas suitable for DL breeding. We aggregated hopper and band occurrence records dated 2010-2021 and denoted these as ‘presence data’. We cross-referenced locations in the ‘Ecology’ class that had no reports of hoppers or bands within a 1 km radius and assigned these entries to ‘absence data’. Traditionally, trained surveyors monitor hopper and band presence using common practices employed by the FAO and national ministries [81]. Surveys are planned in traditional locust habitats or in places where locusts were previously reported [81]. In total, there were 13,390 locations with hopper/band presence and 6,578 locations in which hopper/band were absent. We used 80% of the data for model training and 20% of the data for model validation. Three static variables were used: elevation, sand content in soil at 5 cm depth, and clay content in soil at 5 cm depth (breeding suitability sub-model in Fig 1). Similar concepts of predicting breeding regions have been applied previously using a range of factors, for example, temperature, rainfall, sand and moisture contents [26]; soil moisture and soil texture [27], or using soil moisture time series only [37].

Calculations to produce maps of breeding suitability across the five countries in the domain of interest were performed on the Google Earth Engine platform [66]. We used a Random Forest [82] classifier with the following options: the number of decision trees was set to 100, the number of variables per split was set to 2, the minimun leaf population (i.e. the end node) was set to 2, and the initial seed was set to 0. The ‘Out-of-Bag’ error associated with prediction did not decrease significantly when the number of decision trees was set higher than 100, in accord with results for random forest methods reported by [83]. The importance of covariates was obtained by normalising the classifier importance values. The breeding suitability values were adjusted to a 0-100 scale. We used the R package PresenceAbsence [84] for evaluating the results of breeding suitability predictions based on validation data. For details see section S4 in S1 Appendix.

#### Egg laying

We model a coherent unit of gregarious locusts at a similar developmental stage, here at the stage of breeding and egg laying. First, we determined if the location was suitable for egg laying by drawing a number from a uniform random distribution and testing if the number was smaller than the breeding suitability value for the location (transition from adults to eggs in Fig 1). A second condition that there was sufficient moisture in the soil for egg laying required either a precipitation event (precipitation > 0) or soil moisture to be above 12 mm, at any time 24-48 hours prior egg laying [27, 85]. For details see section S5 in S1 Appendix.

#### Development from eggs to hoppers

The percentage daily development of eggs can be expressed as a function of temperature (in Celsius) [86]:

$$\begin{array}{c} g_{egg}\left(T\right)=\{\begin{array}{cc} 9.41\times \text{exp}(-0.00357*{\left(35.019-T\right)}^{2}), & \text{if}\;10\leq T\leq 34\text{,}\\ 0, & \text{otherwise.} \end{array} \end{array}$$ (1)

We used the UK Met Office NAME environment to provide temperature values at 3 hourly time intervals (development period sub-model in Fig 1). The development period of eggs was obtained by integrating Eq 1 until the first day it reached 100%. If the calculated egg development period was within 11-50 days [87], we assumed that egg development to hoppers was successful, otherwise it was assumed to be unsuccessful. For details see section S6 in S1 Appendix.

#### Development from hoppers to adults

The percentage daily development of hoppers can be expressed as a function of temperature (in Celsius) [88]:

$$\begin{array}{c} g_{hopp}\left(T\right)=\{\begin{array}{cc} 0.222\times T-3.166, & \text{if}\;23\leq T\leq 32\text{,}\\ 0, & \text{otherwise.} \end{array} \end{array}$$ (2)

We used the UK Met Office NAME environment to provide temperature values at three hourly time intervals. The development period from hoppers to adults at each location following hopper emergence was calculated by integrating Eq 2 until the first day it reached 100%. Here we assume that when locusts reach the adult stage they have sufficient fuel (fat) for long-duration flight. If the calculated hopper to adult development period was within 24-95 days [88], we assumed that adult emergence i.e. fledging was successful. A second criterion for the development of hoppers to adults involved the availability of vegetation (transition from hoppers to adults in Fig 1). Hoppers are known to prefer fresh vegetation for feeding [89]. Environmental conditions for hopper feeding and subsequent fledging as adults were deemed to be suitable in our model if either of two conditions were satisfied during the hopper development period: (i) smoothed NDVI was increasing; or (ii) smoothed NDVI was above a threshold. The NDVI threshold of 0.09 was chosen for the Red Sea region: for other regions, the threshold can be changed, for example a value of 0.14 has been proposed for Mauritania in the western Sahel [90, 91]. For details see section S7 in S1 Appendix.

#### Estimating feeding capacity at swarm landing sites

The aim was to evaluate how vegetation at swarm landing sites could satisfy feeding requirements of a swarm and to translate that into a potential feeding period for a swarm at a given location. We assumed that a swarm stays at a location for as long as there is vegetation for feeding and then embarks on long-distance windborne movement. To derive rules for estimating feeding periods, we: (i) analysed the relationship between land cover types and NDVI values within the study area; (ii) reviewed the literature for information on the relationship between land cover type, NDVI, and above ground biomass; and (iii) reviewed the literature for information on food requirement for an average swarm.

We investigated three factors that were considered to be important when determining food availability: land cover type, the current value of NDVI (vegetation density), and NDVI trend (increasing, decreasing or constant) at a swarm landing site (feeding capacity sub-model in Fig 1). We aggregated vegetation classes in the Copernicus global map CLC100 into four groups: (i) bare/sparse vegetation, under the name sparse vegetation); (ii) shrubs, herbaceous vegetation and herbaceous wetland, under the name shrubs; (iii) cultivated and managed vegetation/agriculture, under the name cropland; and (iv) closed forests (5 classes) and open forests (6 classes), under the name forest. Depending on the value of the NDVI, the vegetation state on the ground was classified into six classes [92]: no vegetation (NDVI<0), lowest density (NDVI ∈ [0, 0.15)), lower density (NDVI ∈ [0.15, 0.3)), dense vegetation (NDVI ∈ [0.3, 0.45)), higher density (NDVI ∈ [0.45, 0.6), highest density (NDVI ∈ [0.6, 1]). We collated typical ranges of above ground biomass corresponding to a particular land cover type and NDVI range from the literature. Next, we extracted from the literature the amount of food consumed by a swarm. Based on the values of these factors, we derived rules for estimating feeding periods at a swarm location (see Section below for further details). Briefly, we aligned potential amount of above ground biomass with a typical range of food consumed by a swarm. Finally, we divided feeding periods into three classes: short stay (1-2 days), medium stay (2-4 days), and long stay (4-7 days) based on land cover type, NDVI trend (increasing, decreasing or constant) and vegetation state (value of NDVI). For additional details see section S8 in S1 Appendix.

#### Simulating movement of swarms

To simulate day to day movement of swarms, a single NAME wind trajectory was sampled from the pre-calculated 1000 trajectories for a given location (migration sub-model in Fig 1). We assumed that the propensity of a swarm to start flying in the upcoming days depended on food availability i.e. feeding capacity at the swarm location. Swarms start flying 2h after local sunrise and terminated their flight 1 h before local sunset [74]. On the morning of the day a swarm flies, we searched for the closest grid coordinates, randomly selected a single trajectory and translated the corresponding wind trajectory to start at the required location (Fig EE in S1 Appendix). The whole swarm takes to the air two hours after sunrise and starts to settle about an hour before sunset [93]. We assumed that swarms move downwind with ground speed equal to wind speed [94].

Instances of windborne displacements of DLs from Africa to the Caribbean and South America have been reported, but these events are extremely rare and require specific meteorological conditions, such as temperature inversion with very warm air between 400 and 1600 m above sea level [95, 96]. We assumed that during the period we are interested in conditions were not suitable for inter-continental transport, so wind trajectories leading to the ocean were stopped at the closest grid cell on land.

#### Characterising wind trajectories for long-distance swarm dispersal

An overarching aim was to identify time periods when wind direction was suitable for swarms to reach Kenya from breeding sites in Somalia. We chose locations that could act as stepping stones for swarm movement. Analysis was done as in [97]. We calculated the angle between a vector corresponding to the East direction and a vector connecting the start and end location of a trajectory. This was repeated for all wind trajectories from January 2020 till December 2021. Next we fitted the von Mises distribution to daily wind trajectories in chosen locations. The von Mises distribution has two parameters: the directional mean and concentration. Finally, we plotted temporal change in values of fitted parameter. For details see section S9 in S1 Appendix.

### Testing the model performance

The principal test of the model performance was to assess how well the model components fit together to mimic observed patterns of the DL upsurge during 2019-2021 and to reproduce long-distance movements of DL swarms during the upsurge. We simulated breeding, hopper and swarm emergence in Somalia during September 2020 and assessed whether simulations of subsequent swarm migration and feeding were consistent with the reported observations of swarms arriving in Kenya, between four and five months later. In the absence of detailed observational data on the individual components, the outputs (breeding site maps, development rates, seasonal consistency of wind direction, and sources of food) for each component were assessed against published data and expert knowledge.

To test the performance of the model in simulating long-distance swarm movement we first ran model simulations from a limited breeding area in northeast Somalia in early September. We ran simulations, until 25 successful breeding, development and migration histories were obtained. This was done to test if the model could simulate DL histories consistent with observed reports, i.e. swarms reaching the northeast Kenya from mid December 2020 to mid January 2021. Finally, to test the robustness of the model, we ran 1,000 model realisations from an extended region in northern Somalia. This allowed us to draw maps for two statistics: the expected date of arrival and the proportion of trajectories that visited a location. The expected date of arrival was calculated as the median of the dates when trajectories visited a location.

## Results

We first present results for the components of the modelling framework using meteorological, remote-sensed and soil data for the period corresponding to the most recent DL upsurge in East Africa (Sept 2019—Dec 2021). The components encompass breeding sites, nymph and adult emergence, feeding potential at breeding and landing sites and wind-dispersed swarm dispersal. Analyses were conducted at 1 km × 1 km resolution across the domain encompassing Ethiopia, Kenya, Somalia, Djibouti and Eritrea. We then validate the modelling framework by testing the ability of the integrated model to reproduce long-distance migration of DL swarms from breeding sites in northern Somalia to reach Kenya based on surveillance data from the 2019-2021 upsurge. Finally, we show how the model can be used for short-term forecasting of swarm migration using near real-time weather forecast data.

### Areas suitable for breeding

**Fig 3:**
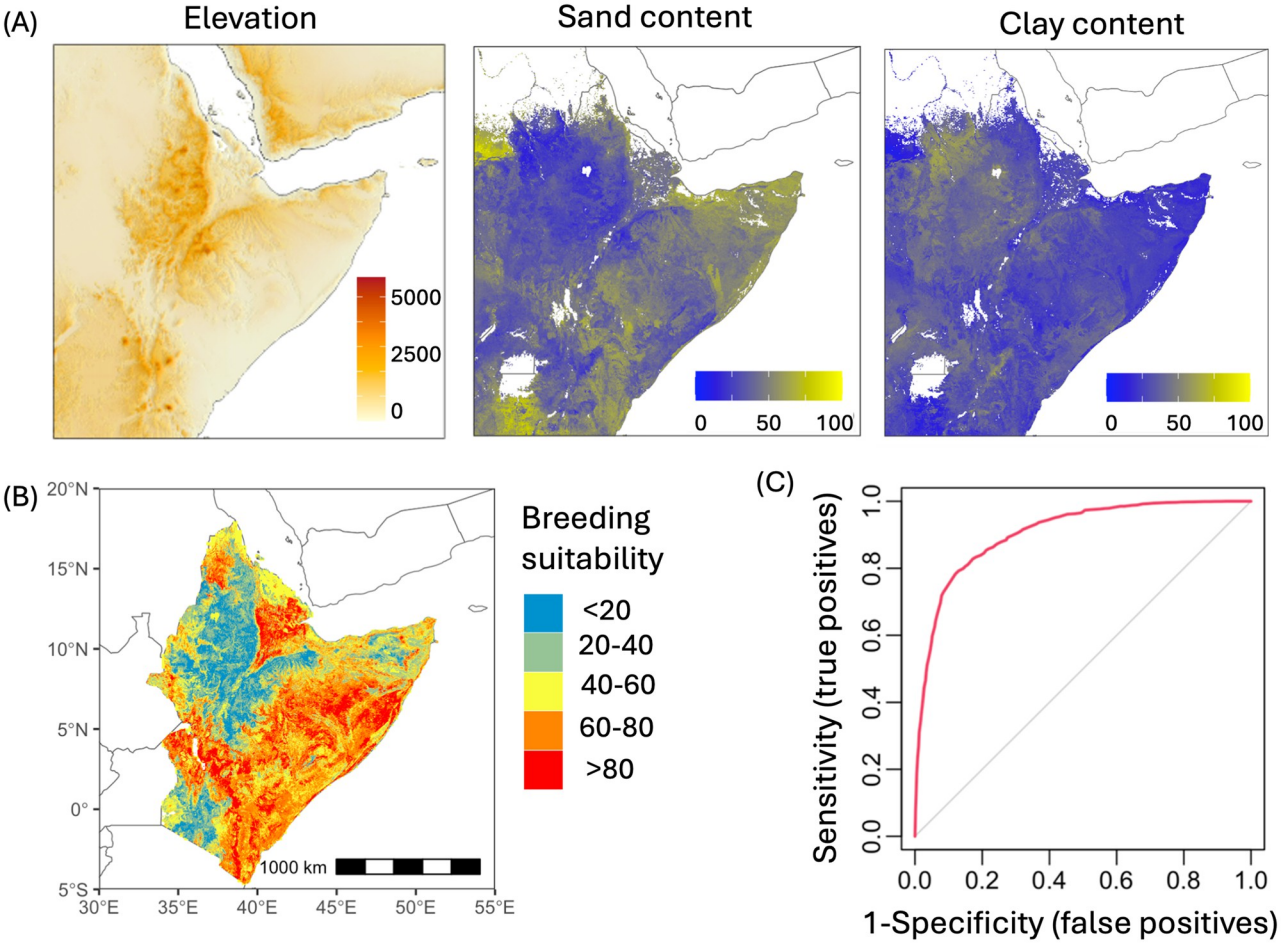
Suitability for breeding. (A) Covariates used for predicting suitability of breeding: elevation, sand content and clay content. (B) Predicted breeding suitability map. (C) Model validation (based on 20% of data retained from model training). The base layer of the map is sourced from Natural Earth (https://www.naturalearthdata.com).

The fitted model showed that all three covariates (Fig 3A) had similar importance for desert locust breeding sites: with elevation accounting for 32% of the variation, sand content 34%, and clay content 34%. A suitability map for desert locust breeding sites is shown in Fig 3B. Using a normalised score of 0-100 our results indicate that 43% of 1 km × 1 km grid cells had breeding suitability higher than 60, and 13% probability higher than 80 on a normalised scale of 0-100. Fig 3C shows the area under the curve of the Receiver Operating Characteristic (ROC) curve for the validation data set. We obtained the following accuracy statistics values: percent correctly classified = 0.83, sensitivity = 0.9, specificity = 0.71, and area under the curve = 0.91. The map (Fig 3B) shows considerable heterogeneity within countries and across the domain of interest. The general patterns are consistent with previously published maps e.g. [26] but with higher degrees of spatial heterogeneity consistent with the resolution of the model (Fig S in S1 Appendix).

### Distribution of development periods for eggs to hoppers and hoppers to adults

**Fig 4:**
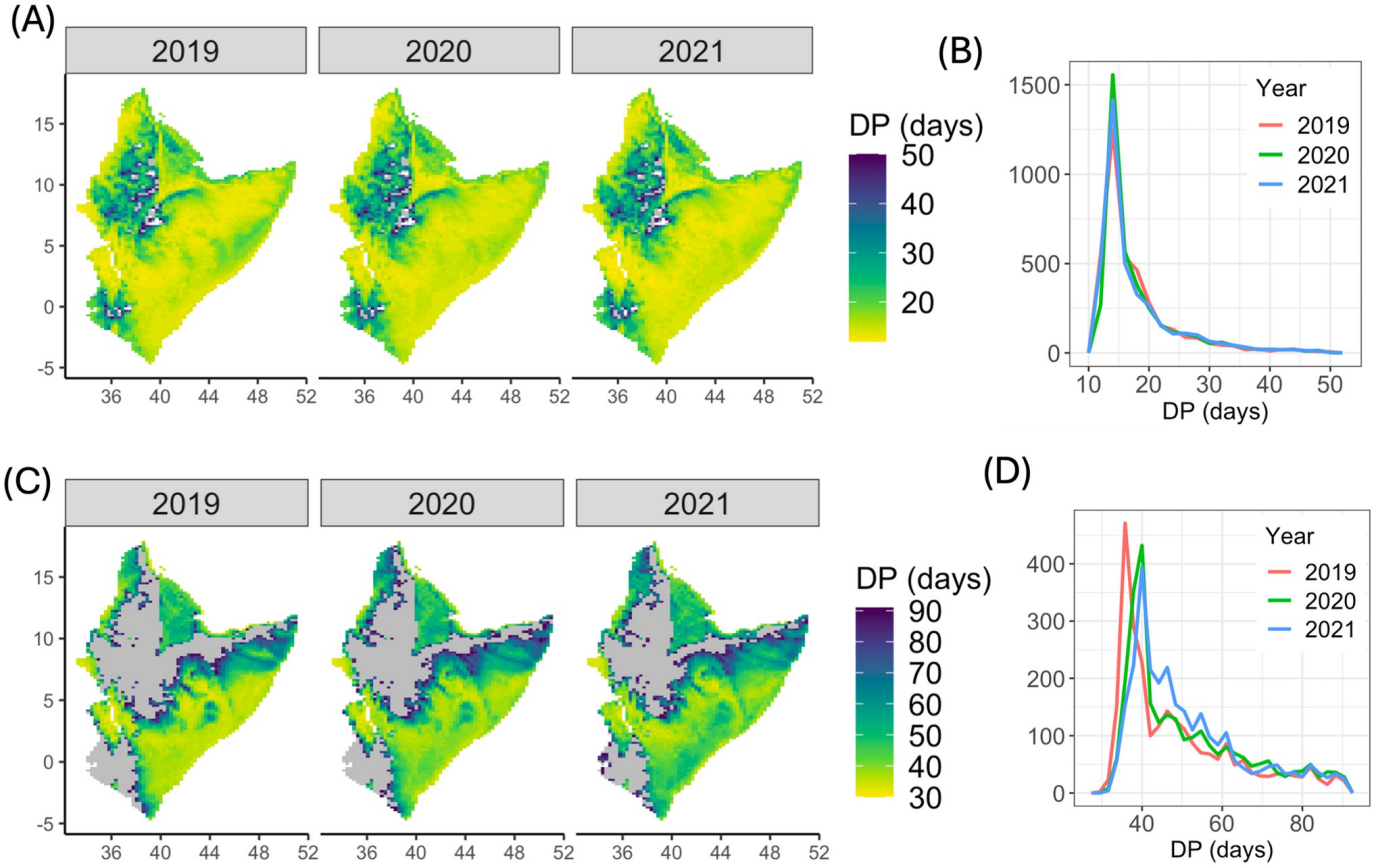
Development periods. (A-B) Spatial distribution and histogram of egg development periods (DP) for September 2019, September 2020 and September 2021. (C-D) Spatial distribution and histogram of hopper development periods (DP) for October 2019, October 2020 and October 2021. Gray shows areas which are not suitable for development. The base layer of the map is sourced from Natural Earth (https://www.naturalearthdata.com).

The spatial distributions of the duration of egg development to hoppers are shown in Fig 4A for eggs deposited in the study area on 1^*st*^ September 2019, 1^*st*^ September 2020 and 1^*st*^ September 2021. There was considerable variation in the development period from eggs to hoppers across the domain but marked consistency in the spatial distributions on corresponding dates in successive years: the average egg development period was 18.8 days for 2019, 19.1 days for 2020 and 19.0 days for 2021 (Fig 4B). The spatial distribution of the duration of hopper development into adults also showed substantial spatial variation across the domain on a selected date (1^*st*^ October) for three years (2019, 2020 and 2021) (Fig 4C). The average development period from hoppers to adults was 48.5 days for 2019, 50.9 days for 2020 and 51.2 days for 2021 (Fig 4D).

### Length of stay for swarms

**Fig 5:**
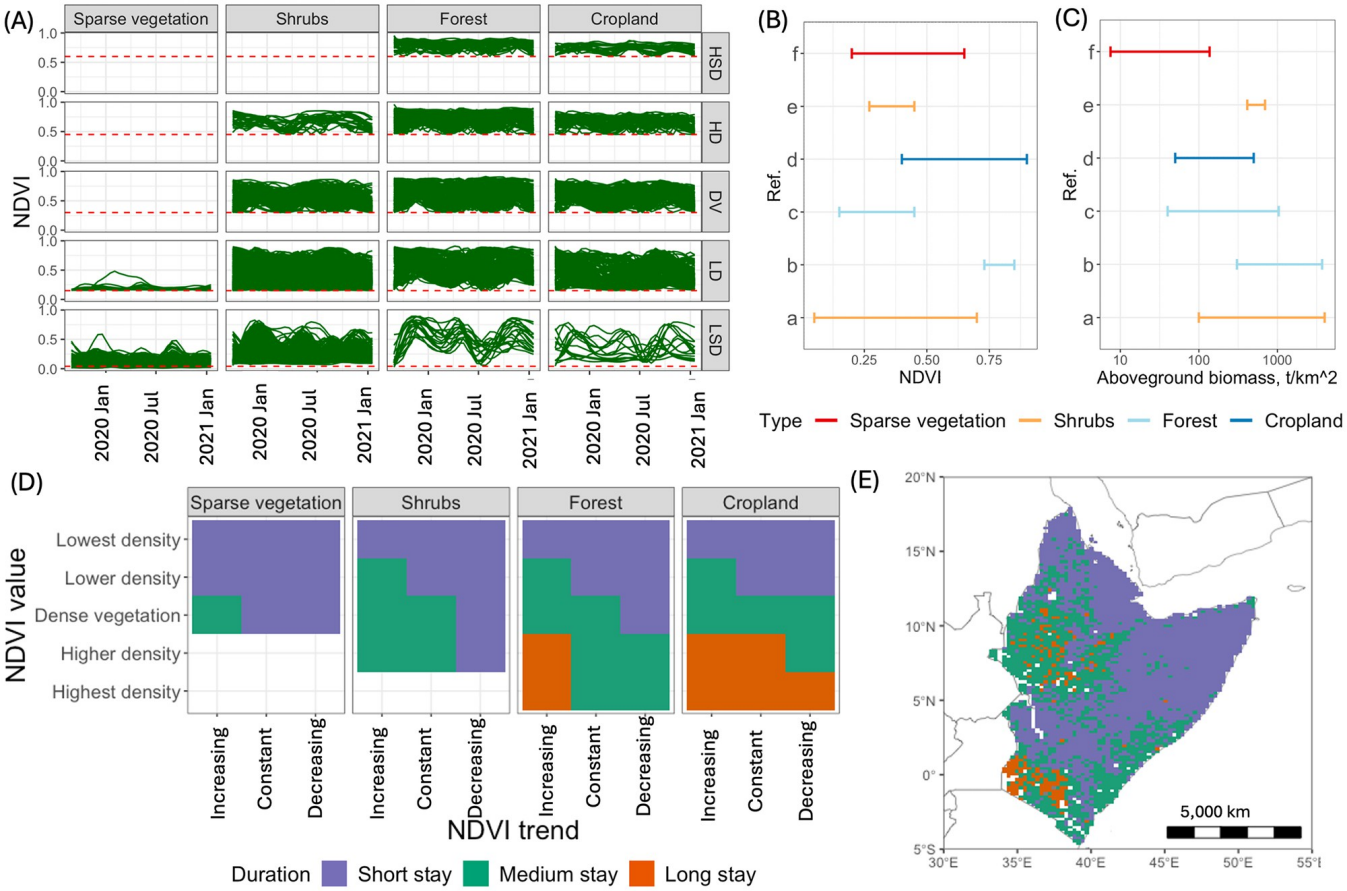
Evaluating length of stay for swarms. (A) Smoothed NDVI profiles classified according to land cover type (sparse vegetation, shrubs, forest and cropland) and vegetation state (highest density (HSD), high density (HD), dense vegetation (DV), low density (LD), lowest density (LSD). The minimum NDVI is shown as a red dashed line. (B)-(C) Ranges of NDVI and aboveground biomass: sparse vegetation (a- [98]), shrubs (b- [99], c- [100]), forest (d- [101], e- [102]) and cropland (f- [103]). (D) Length of stay for swarms depends on land cover type, NDVI trend (increasing, decreasing or constant) and vegetation state (value of NDVI). Here short stay is 1-2 days, medium stay is 2-4 days, and long stay is 4-7 days. (E) Spatial distribution of potential length of stay on 15^*th*^ January 2021. The base layer of the map is sourced from Natural Earth (https://www.naturalearthdata.com).

The surface phenology of each land cover group was inferred from the temporal profiles of NDVI [104] from which we inferred food availability and length the of stay at swarm landing sites. Fig 5A shows smoothed NDVI profiles classified according to land cover type and vegetation state. We can identify certain characteristics of vegetation availability. For example, the highest density of vegetation is possible only for forest and cropland classes.

We collated the available literature on ranges of aboveground biomass for different types of land cover. Analysis based on mixed gardens showed that NDVI values between 0.73 and 0.85 correspond to the total carbon stock between 0.1 and 1.8 tonnes per 30 m × 30 m pixel [102]. The estimation of carbon content can be obtained by multiplying the result of aboveground biomass measurements with a 47% conversion factor [105]. This allowed us to convert carbon content back to aboveground biomass. For mangrove forest, NDVI values between 0.15 and 0.45 correspond to aboveground biomass between 1 and 26 kilograms per 5 m × 5 m pixel [101]. For crops, NDVI values ranging from 0.4 to 0.9 correspond to aboveground biomass between 0.5 to 5 tonnes per hectare [103]. For shrubs, NDVI values ranging from 0.05 to 0.7 correspond to aboveground biomass between 0 to 40 tonnes per hectare [100], and NDVI values ranging from 0.3 to 0.6 correspond to aboveground biomass between 4 to 7 tonnes per hectare [99]. Fig 5B and 5C shows the ranges of NDVI and the ranges of aboveground biomass scaled to tonnes per square kilometre for the four land cover types used in the analyses.

The daily feeding-rates of individual desert locust vary with age and activity: adults eat roughly half their own weight (≈1.0 g), while a migrating adult swarming locust would need to eat its own weight and possibly three times as much (2.0 g—5.0 g) [106]. There is wide variability in estimates of the density and size of locust swarms: from 25 locusts per square metre [106] to 10^9^ locusts in a swarm covering an area of 20 square kilometres [107]. This gives a range of locust density between 3.5 × 10^7^ to 5 × 10^7^ locusts per 1*km*^2^. If we assume that a locust eats its own weight (≈2.0 g), then the required amount of food consumed by a swarm per square kilometre would be between 70 and 100 tonnes of plant material per day.

Based on the calculated amount of food consumed by a swarm and aboveground biomass availability, we concluded that certain combinations of land cover type and NDVI characteristics should provide food for swarms for up to 7 days. We divided the length of stay of DL swarms into three classes: short stay (1-2 days), medium stay (2-4 days), and long stay (4-7 days). Fig 5D shows how length of stay depends on the situation on the ground. A snapshot of the spatial distribution of potential length of stay on is shown for 15^*th*^ January 2021 in Fig 5E. The number of days a swarm stays and feeds was sampled from the range corresponding to the class to which the location was assigned. For NDVI values less than zero, swarms would stay only over night and start flying in the morning.

### Modelling wind trajectories involved in swarm dispersal

**Fig 6:**
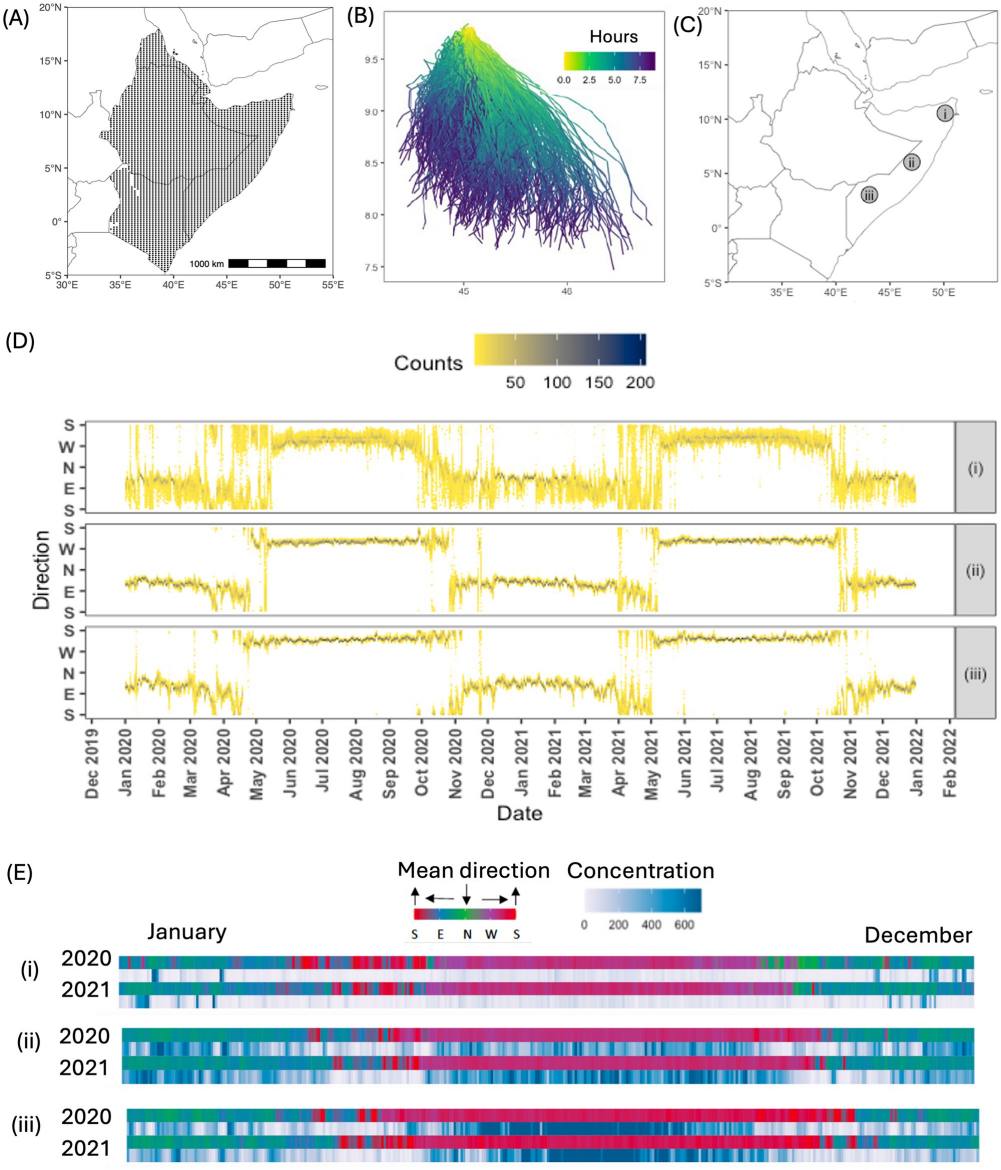
Characterisation of wind trajectories involved in swarm dispersal. (A) Source locations for wind trajectories. (B) An example of NAME wind trajectories on the 15^*th*^ January 2021. The colour shows the difference in hours between points on a trajectory and the initial point. (C) Chosen locations. (D) Distribution of daily wind direction at the three chosen locations shown in (C) during 2020-2021. (E) Daily values of fitted mean direction (top row) and concentration (bottom row) at the three locations shown in (C) for 2020 and 2021. Mean directions from: South (S), East (E), North (N), and West (W). A concentration value 1 is approximately a width of 140°, and value 600 is approximately a width of 4.7°. The base layer of the map is sourced from Natural Earth (https://www.naturalearthdata.com).

We assume that DL swarms are aided by wind-dispersal. The sites for which 1000 individual NAME trajectories were computed daily between 1^*st*^ September 2019 and 31^*st*^ December 2021 for each of 3860 source locations spaced on a regular 20 km × 20 km grid are shown in Fig 6A. An illustrative set of trajectories for potential swarm dispersal from a single site on a single release day is given in Fig 6B. The results of fitting the von Mises distribution to three sites (Fig 6C) are summarised in Fig 6D and 6E for for 2020 and 2021. Temporal changes in the directional mean show seasonal variation in global wind patterns, while the concentration serves as a measure of stochasticity associated with natural variation in turbulent wind flow [97]. Changes in wind direction are evident (Fig 6D and 6E). The fitted mean direction values indicate winds directed from the north between December and January across all three locations in both 2020 and 2021. In contrast, during summer months the wind was directed from the south at two locations, with high concentration values (>600) during most days.

### Integrating the components to simulate breeding, nymph and adult emergence, migration and feeding

**Fig 7:**
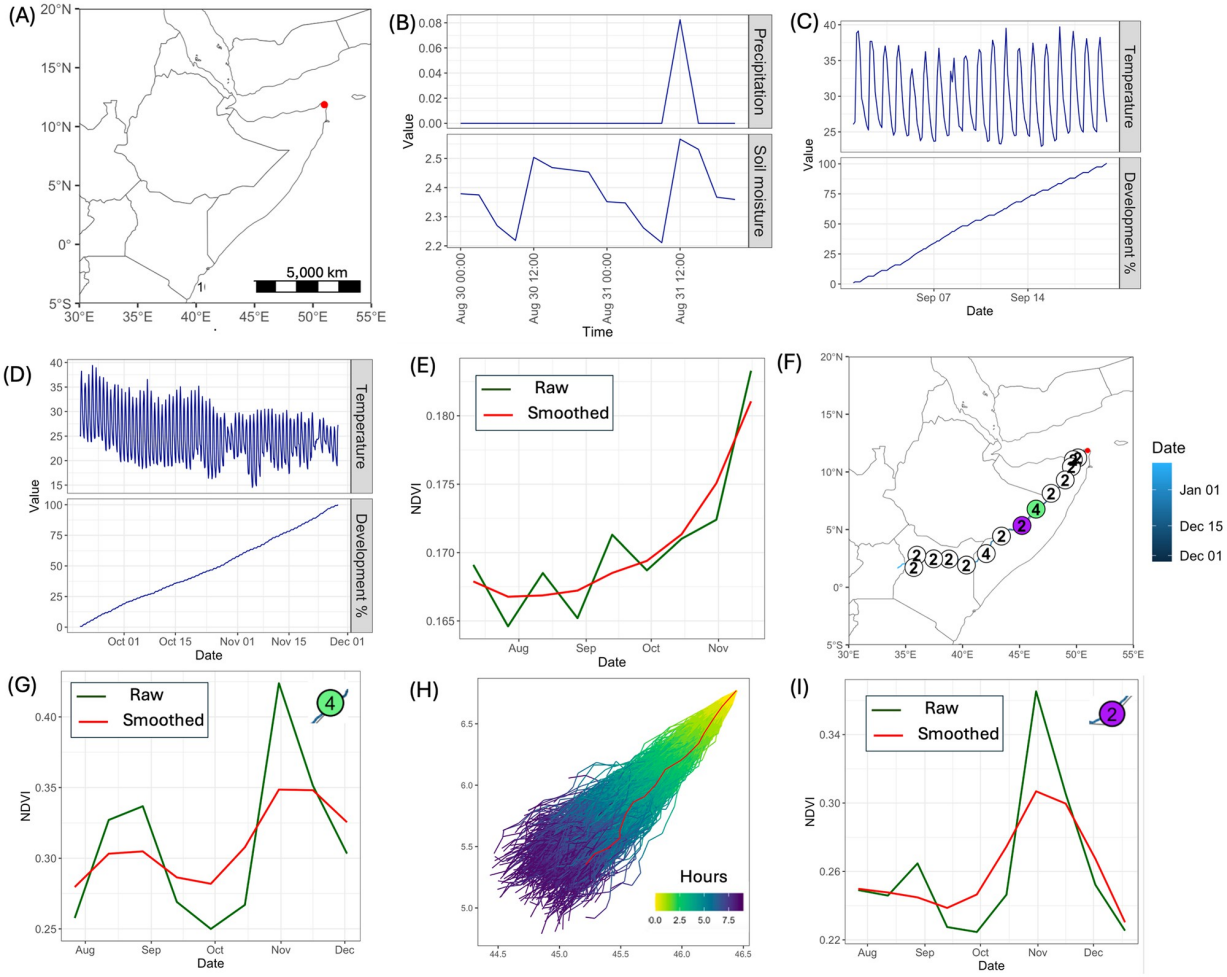
Integrating the components of the framework. (A) Breeding location. (B) Precipitation and soil moisture up to 48 hours before egg laying. (C) Temperature profile and change in development from egg to hopper. (D) Temperature profile and change in development from hopper to adult. (E) Raw and smoothed NDVI profiles at the breeding site from July to December 2020. (F) Swarm movement pathway. Circles indicate landing sites, and numbers show how long swarms stayed. (G) Raw and smoothed NDVI profiles at the location shown in green in (F) Date swarms were at the location is 13^*th*^ December 2020. (H) NAME wind trajectories on the 29th December 2020 from the location shown in green in (F). The colour shows the difference in hours between points on a trajectory and the initial point. (I) Raw and smoothed NDVI profiles at the location shown in purple in (F). Date swarms were at the location is 18^*th*^ December 2020. The base layer of the map is sourced from Natural Earth (https://www.naturalearthdata.com).

We illustrate the integration of the model from a single site in north east Somalia and based on a single randomly selected trajectory. Simulation was started by choosing the location and date for breeding (Fig 7A). The date of egg laying was set as 1st September 2020. We checked if the location was suitable for egg laying. For this particular location, the probability of being suitable for breeding was 0.65. The precipitation profile showed that there was an episode of rain the day before (Fig 7B). Based on temperature values, the predicted date for hopper emergence was 19^*th*^ September 2020 (Fig 7C) with adult emergence on 28^*th*^ November 2020 (Fig 7D). Next, we tested if there was enough vegetation available for feeding. The values for NDVI were above the threshold (≥ 0.09) during the time when hoppers hatched and became adults (Fig 7E). The final step involved migration of swarms, which involved testing how long the vegetation at the breeding site could sustain a swarm, which was a function of land cover and NDVI, as well as swarms following prevailing winds. An example of a swarm migration pathway together with landing sites and days stayed is shown in Fig 7F. We illustrate the state of vegetation at two landing sites shown in green and purple. Land cover type was shrubs for both sites. For the first landing site, the smoothed NDVI profile suggested a medium feeding stay (Fig 7G). An ensemble of NAME wind trajectories from the landing sites on the 29^*th*^ December 2020 (after 4 days of feeding) is shown in Fig 7H. Single trajectory was randomly selected to advance swarm migration (shown in red in Fig 7H). At the new landing site, the smoothed NDVI profile suggested short feeding stay (Fig 7I).

This analysis for integration of the framework is available as a tutorial using the R markdown language (see S12 in S1 Appendix).

### Model output analysis

**Fig 8:**
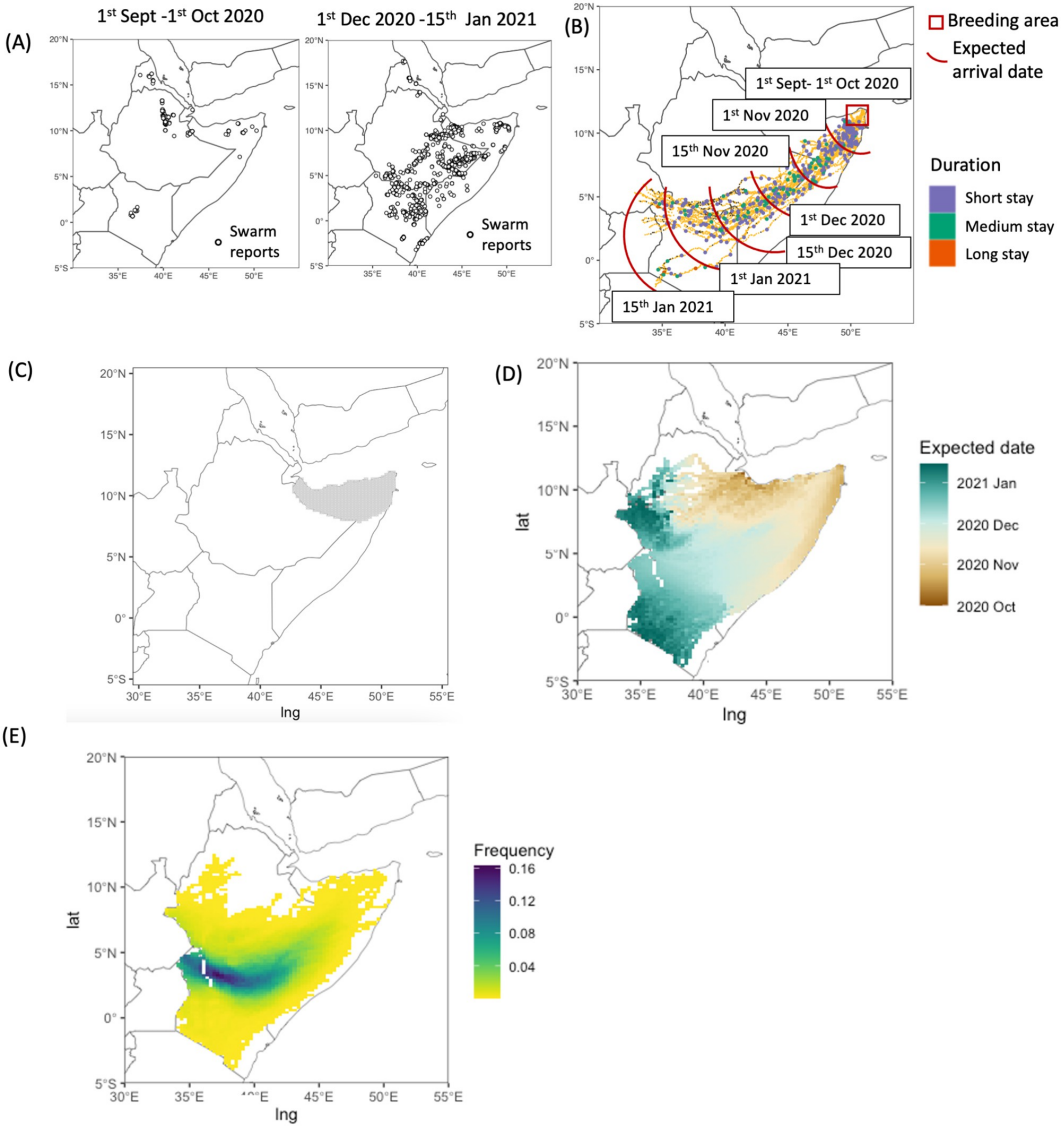
Model output analysis. (A) Locations of reported swarms from Locust Hub during two periods. (B) Simulated breeding, development and migration of DL. Red rectangle indicates areas that were sampled as a breeding ground. Lines show simulated DL migration trajectories. Circles indicate landing locations and are coloured according to the duration swarms stayed. Red curves indicate expected date when swarms reached a particular area. (C) Sites where model simulations started on 1st of September. (D) Distribution of expected arrival dates. (E) Distribution of fraction of simulations passing through grid cell. The base layer of the map is sourced from Natural Earth (https://www.naturalearthdata.com).

We chose two periods from the reported swarm locations [67]: (i) 1^*st*^ September—1^*st*^ October 2020; and (ii) 1^*st*^ December 2020—15^*th*^ January 2021. The locations of swarms during these two periods are shown in Fig 8A. We chose a breeding time in September 2020, based on FAO data and reports of the situation in the Horn of Africa [108]. We simulated locust breeding, development, feeding and dispersal using historic weather data from 1^*st*^ September 2020 until 15^*th*^ January 2021. The second period (1^*st*^ December 2020—15^*th*^ was used to test for arrival times in Kenya, with progressive cycles of feeding and swarm dispersal between breeding and later detection in Kenya. First, we ran simulations from a small breeding area, shown as a red square in Fig 8B. This served as a pilot study and confirmed that the model can produce cases in which the temporal and spatial dynamics are consistent with the situation recorded during the actual DL upsurge.

Next, we extended the potential breeding area to cover a larger region corresponding to the territory of Somaliland and north east Somalia (Fig 8C). This region has large areas with high and others with low breeding suitability values Fig 3), so at least 30% of simulations were terminated at the early stages. Overall, conditions based on precipitation and soil moisture were favourable for breeding and egg laying. Temperature values were favourable for egg development, giving a development period between 11 and 16 days. That is consistent with laboratory studies, in which the duration of the egg development period was 11-12 days at 35-41°C [87]. Our simulations indicated that the hopper development period had a wider range (between 25 and 50 days). These results are in close agreement with those obtained from laboratory experiments, in which the hopper development period varied from 25 days at a mean temperature of 32°C to approximately 50 days at 24°C [109].

The seasonal variation of swarm migration is driven by the shift in the Intertropical Convergence Zone [110]. Winds emanating from the north have been associated with DL displacement from Somalia to Tanzania during multiple DL upsurges over the last century [86, 111]. Our simulations confirmed that wind conditions were favourable for long-distance migration towards the southwest (Fig 8D). By considering a larger breeding area, we could observe the effect of stochasticity arising from different initial locations on the subsequent displacement of swarms. The destinations of migration pathways had locations ranging between South Sudan and Tanzania. The spread of swarms reaching multiple countries is not unusual, having been observed, for example, during the 2003-2005 desert locust upsurge in West Africa [112].

Our analysis clearly shows that swarms originating from breeding sites in Somalia would reach Kenya during the second half of December (Fig 8B–8D). The expected date of arrival was similar across both the border between Somalia and Kenya, and the border between Ethiopia and Kenya. However, our simulations indicated differences in high risk areas, i.e. areas that had higher frequency of trajectories reaching them due to converging wind trajectories (shown in dark blue in Fig 8E). These areas were consistent with real-world swarm presence between December 2020 and January 2021 (shown in Fig 8A). If the simulation had continued beyond 15^*th*^ January 2021, the swarms would have moved further to the west and reached Uganda.

### Short term prediction of swarm migration

Here we investigate how the model could be used for short-term (1-2 days) forecasting of swarm migration. To mimic real-life conditions, we assumed that swarms are most likely to be spotted and reported while they are flying between landing sites. Hence, the only data available are the co–ordinate and date of the sighting, with no information about flight direction, which is notoriously difficult for observers on the ground to assess [113], nor is it usually known where a swarm has come from.

**Fig 9:**
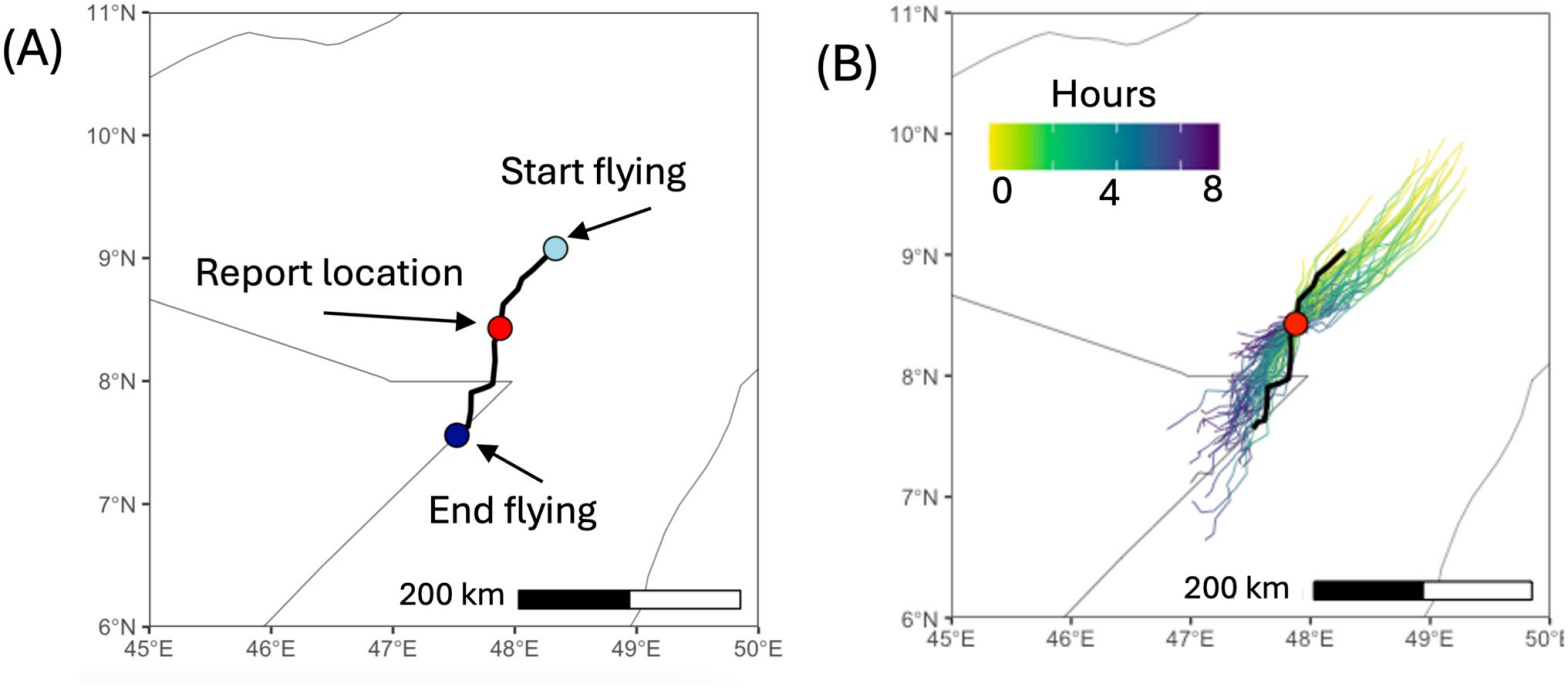
Short term prediction. (A) Simulated swarm migration trajectory (black line) and reported location (red dot). (B) Possible migration trajectories on reporting day passing close to the reporting site. The colour shows the difference in hours between points on a trajectory and the initial point. The base layer of the map is sourced from Natural Earth (https://www.naturalearthdata.com).

We considered a reporting location at the border between Somalia and northern Ethiopia (Fig 9A). The forecasting of DL movement was simulated as follows. First we sampled 10,000 flight start coordinates within a 250 km radius around the reporting site. We sampled a trajectory from each starting coordinate for the day of reporting. Only those trajectories that passed within 5 km or closer to the reporting location were retained (see section S10.2 in S1 Appendix). The landing points of these trajectories could then be used to derive a risk map of swarm locations at the end of the reporting day (dark blue trajectories in Fig 9B). If the time-of-day of the swarm sighting was known, this could be incorporated as an additional filter into analysis and could provide a refined risk map. The modelling framework can in principle be used to simulate locust migration and feeding for up to seven days in order to mimic a seven-day weather forecast analogous to those used to forecast wheat stem rust [61].

## Discussion

We have developed an integrated modelling framework to predict and analyse the population dynamics and large-scale dispersal of desert locust, motivated by the recent (2019-2021) upsurge in East Africa. The key features of our model are identification of suitable breeding sites, which is combined with egg and hopper development, and swarm movement, which is influenced by turbulent wind flow. The model also accounts for the feeding behaviour of DL, where the duration swarms spend at a landing site is determined by the availability of food at the site. Our aim was to provide a cohesive and comprehensive framework based on the available data and current understanding of DL biology. As new data emerge and knowledge develops, the framework provides an opportunity to advance the model by refinement of sub-models.

Previous modelling studies on DL relied predominantly on statistical approaches to analyse time series data and lacked explicit mechanisms to account for DL population dynamics and especially for swarm movement. For example, Sun *et al.* [40] used data for elevation, land cover, sand and clay content in soil, soil moisture, NDVI and surface temperature, combined with historical locust ground survey data to produce dynamic forecasts of DL hopper band occurrence. The approach in [40] did not, however, extend to accounting for the migratory behaviour of desert locust swarms that arise from hopper bands, something we address in the integrated modelling framework introduced here. Sorel *et al.* [45] designed an agent-based model where the time available for swarm dispersal was reduced by time spent feeding. Our sub-model for estimating feeding capacity at swarm landing sites is based on a similar hypothesis that length of stay depends on the state of the vegetation. However, our proposed modelling framework integrates not only feeding and movement of swarms, but the whole life cycle of DLs with environmental conditions. Mamo *et al.* [22, 23], meanwhile, used deterministic ordinary differential equations to model the effects of early intervention on DL upsurges and impacts on crop production. The models of Mamo *et al.* [22, 23] provide valuable mathematical insights into the dynamics of control at given sites, but the models do not account for uncertainty due to weather-driven variability nor for swarm movement. Magor *et al.* [114] also analysed early control intervention in DL campaigns, using historic data and a simple model with a locust multiplication factor dependent on breeding conditions at a given site. In our model, the dynamic conditions of breeding sites are incorporated by recent precipitation events in addition to the intrinsic suitability of soil conditions. We also allow for turbulent wind-assisted dispersal of DL swarms by coupling breeding and emergence of hopper bands with a weather-driven dispersal model, interspersed with variable feeding durations at landing sites.

Migrant pests such as DL could be considered to be metapopulations occupying discrete habitat patches, with colonization and extinction rates defining the dynamics of the whole population [21, 115]. Full parameterisation of a metapopulation model requires data on population sizes within the patches, which are not available for the 2019-2021 upsurge in East Africa. Our approach does not require knowledge of the size of DL populations since we model a coherent unit of gregarious locusts at a similar developmental stage. A multi-agent system to represent events of locust plague development and management decisions was designed in [116–118]. The model was built on a virtual territory represented by a grid of 100 cells, each cell being 10 km by 10 km. Similar to our approach, the main entity comprised coherent groups of locusts. Locust dispersal was simulated by swarms randomly moving to nearby cells, which is in contrast to our approach, where we used meteorological and environmental data to influence swarm movement. Recently, the migration paths of DL during the 2018-2020 upsurge in Africa and Asia were reviewed in conjunction with DL survey data and the distribution of suitable locust habitats [119]. However, the authors have not provided details on which meteorological products were used to extract wind direction and wind speed, nor how individual migratory paths were constructed.

In our modelling framework we utilised the ability of the NAME atmospheric dispersion model to reproduce stochasticity associated with turbulent wind flow. Essentially this involves modelling an ensemble of possible trajectories from a starting location, each of which represents one possible pathway through the turbulent atmosphere. In previous studies on wind-assisted insect migration, the direction of migration was assumed to follow a single air parcel trajectory. One popular choice is to use wind trajectories obtained from the HYSPLIT modelling system [120]. HYSPLIT trajectories have been used to study migratory trajectories of many insect species, such as seasonal migration of fall armyworm moths in the USA [58], and trans-Saharan migration of painted lady birds [57]. During the 2019-2021 DL upsurge, NOAA created a web-based app for forward or backward simulation of swarm movement based on HYSPLIT’s air trajectories [56]. Simulated HYSPLIT forward trajectories were also used to asses DL invasion risk to China by Wang *et al.* [121]. Another alternative approach to derive single air parcel wind trajectories is to use the **u** and **v** values of wind speed components derived from re-analysed meteorological data or from weather forecasting models. The displacement of a swarm of migratory locusts of the species *Schistocerca cancellata* was simulated using the directions and intensities of the wind derived from Weather Research and Forecasting model [122]. The advantage of accounting for the inherent stochasticity of wind-assisted dispersal as in the framework proposed here lies in the ability to compare the likelihood of different trajectories and DL landing sites from an initial site. We note that the HYSPLIT modelling platform can also be used to calculate ensemble pathways.

We assumed that swarms followed wind trajectories, but in reality the movement can be more complicated. For example, swarms can move more slowly in vegetated areas [94], or exhibit ‘rolling behaviour’, i.e. a continual exchange between flying locusts and those on the ground [123], that would reduce average ground speed of the whole swarm. These aspects potentially can be incorporated into our modelling framework either by accounting for wind speed and swarm flight height via Draper’s formula [94], or by allowing for within-swarm social dynamics of individual swarms [124]. We assumed that swarms continue flying until just before sunset [86, 93, 125]. Another aspect which could be incorporated into our modelling framework is allowing swarms to terminate their flight if they encounter favourable habitat. A swarm will stop migration when it encounters areas suitable for breeding and egg laying. The modelling framework could be used to include cycles of multiple-generations to investigate how favourable conditions for egg-laying impact the long term migration of swarms.

Our framework follows gregarious locust populations through egg, hopper and adult stages. By separating the breeding process into two parts (the breeding suitability map and recent precipitation events that favour egg laying and development) we can take advantage of the high temporal resolution data on temperature, precipitation and soil moisture provided by the UK Met Office Unified Model. We found broad correspondence between our prediction and predictions at broad scales in [26]. In addition to the increased spatial resolution, an advantage of our approach to predict breeding suitability is that elevation, sand and clay content in soil are invariant to climate change, at least for the current time horizon used for climate change impact evaluation.

Feeding capacity was assessed based on land cover type, NDVI trend and vegetation state. The longest stay was assumed for cropland, with higher and highest density of vegetation and with increasing and constant NDVI value, which indicates pre-peak and peak of crop development stage. There are no direct observations of length of stay available to compare with our simulations. However, there is some circumstantial evidence in that no instances of prolonged locust stay with measurable impact on vegetation were reported during the 2020-2021 DL upsurge [46], while minimal damage was reported to crops [47]. This could be because swarms did not encounter much cropland on their migration path. Our simulations indicated that swarms did not stay long at landing sites; the average length of stay was 2.6 days. Further refining of the rules using observational field data on the feeding behaviour of swarms would be beneficial to improve predictability.

To test the framework, we simulated breeding, development and migration histories for locations and time periods chosen based on the observed field surveillance data. Our simulations showed good correspondence with the observed increase in swarms reaching the northeast of Kenya after 1^*st*^ December 2020. The modelled spread of swarms is in agreement with previous evidence that swarms may fly up to nine hours and may easily move 200 km or more in a day [51, 127].

Our proposed algorithm is particularly useful to model short-term and long-term desert locust migration patterns with limited reporting data. The projected feeding and migration trajectories could be used to guide surveillance. We used historic wind data from the UK Met Office Unified model to map risk for up to seven days. This limit is set by the current availability of weather *forecast* data for generating future wind trajectories for use in the NAME model. A period of seven days should be sufficient for control operations to be implemented [128].

In East Africa, the 2019-2021 DL upsurge was characterised by a high degree of spatial and temporal heterogeneity, with some regions experiencing persistently higher DL invasion frequency [2]. Our proposed modelling framework can help to explain this heterogeneity, and can serve to improve locust monitoring and response efforts. Further work is under way to assess different surveillance methods employed during the 2019-2021 DL upsurge, to perform rigorous parameter estimation of the model, and to investigate how to use the modelling framework to reduce risks from the harmful effects of DL upsurges by including control measures under resource constraints. This preparedness to respond quickly to upsurges will become more critical as climate change influences the distribution of DL [29] and it is therefore important to be able to predict DL migration in new regions. The intention is to have the modelling framework ready now to be used if another upsurge were to occur.

## Supporting information

S1 Tutorial A framework for modelling desert locust population dynamics and large-scale dispersal. (PDF)

S1 Appendix A framework for modelling desert locust population dynamics and large-scale dispersal. (PDF)
