# Supplementary material for: A framework for modelling desert locust population dynamics and large-scale dispersal
Source: PLoS Comput Biol. 2024 Dec 19;20(12):e1012562. doi: 10.1371/journal.pcbi.1012562 (PMC11658591; doi:10.1371/journal.pcbi.1012562)
Supplement: S1 Tutorial — (PDF) [file pcbi.1012562.s001.pdf]

# S1 Tutorial to: A framework for modelling desert locust population dynamics and large-scale dispersal

Renata Retkute, William Thurston, Keith Cressman, and Christopher A. Gilligan

05/11/2024

This document provides a detailed tutorial on the essential steps in using the framework. The code integrates DL breeding, development through egg, hopper and adult stages, feeding and swarm migration with remote-sensed data and weather-driven models for wind trajectories.

## Upload required libraries and functions

```
library(raster)
library(ggplot2)
theme_set(theme_bw())
library(lubridate)
library(RColorBrewer)
library(ncdf4)
library(RNetCDF)
library(rnaturalearth)
library(rnaturalearthdata)
library(dplyr)
library(stringr)
library(spam)
library(segmented)
library(ggspatial)
library(viridis)
source('Utilities.R')
```

## Wind trajectory source coordinates

The Met Office's Numerical Atmospheric-dispersion Modelling Environment (NAME) is used to model a wide range of atmospheric dispersion events . The model (that incorporates a Lagrangian Particle Dispersion Model) can simulate wind trajectories that are stochastic so that an ensemble of trajectories provides a probabilistic distribution of the dispersal of particulates such as spores or insects. NAME wind trajectories were calculated starting from 3860 source locations spaced on a regular 20km x 20km grid across Ethiopia, Somalia, Eritrea and Kenya.

```
src<-read.csv("data/sourcelist_3860.csv", header = FALSE)
colnames(src)<-c("ID", "lng", "lat","Country")
fig + geom_point(data=src, aes(x=lng, y=lat), size=0.1)
```

## Initialisation of simulations

Simulation starts with choosing a location and date for breeding:

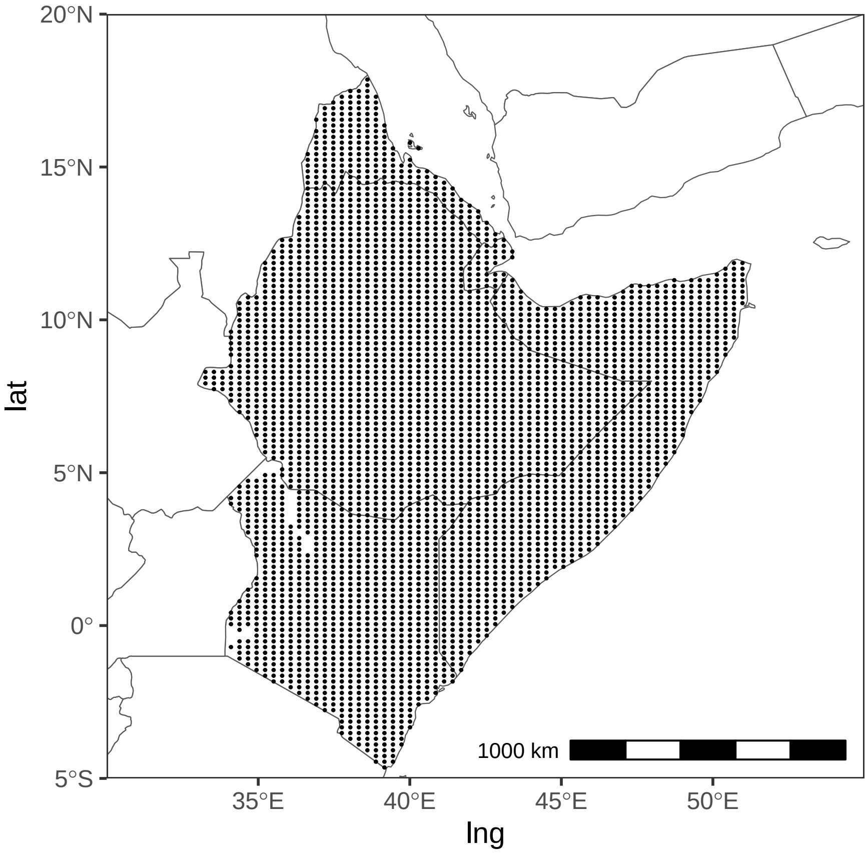


Figure A: Source locations for wind trajectories. The base layer of the map is sourced from Natural Earth (https://www.naturalearthdata.com).

```
coord.eggL<-data.frame(lng=50.97656, lat=11.859380)
date.eggL<-as.Date("2020-09-01")
fig+ geom_point(data=coord.eggL, aes(x=lng, y=lat), col='red', size=1.5)
```

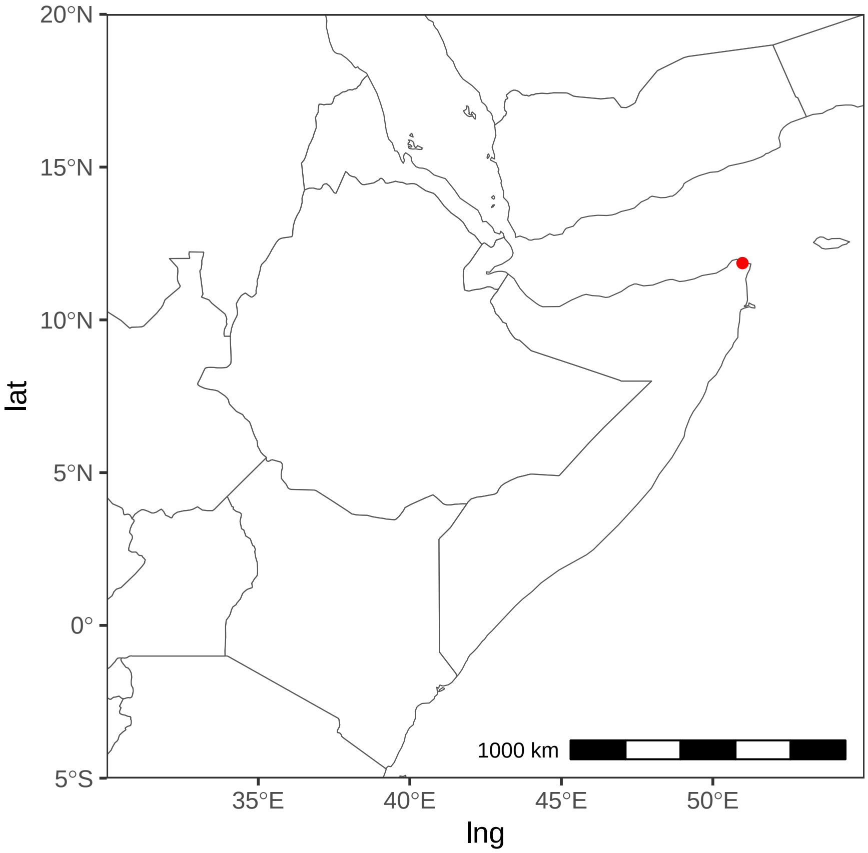


Figure B: Breeding location. The base layer of the map is sourced from Natural Earth (https://www.naturalearthdata.com).

Set random seed for reproducability:

```
rn.sd.1<-14
rn.sd.2<-4
```

Declare variables:

```
ind<-1
BRD<-data.frame(ind=c(), lng=c(), lat=c(), date=c()) # Save breeding data
HPRS<-data.frame(ind=c(), lng=c(), lat=c(), dateS=c(), dateF=c()) # Save hopper development data
ST.DAYS<-data.frame(ind=c(), lng=c(), lat=c(), date=c(), stay=c()) # Save length of stay at landing sit
TRJ<-data.frame(date =c(), lng=c(), lat=c(), flt.tm=c(), ind=c()) # Save migration trajectories
```

Set values of location variables to be equal to the breeding location:

```
lng<-coord.eggL$lng
lat<-coord.eggL$lat
```

## Breeding sub-model

We need to check if location is suitable for egg laying. This uses breeding suitability map.

```r
fln<-"data/breeding_suitability_map_1km.tif"
sbr <- raster(x=fln)
sbr <- rasterToPoints(sbr)
sbr<-as.data.frame(sbr)
colnames(sbr)<-c('lng', 'lat', 'sbr')
pal<-c('#2891C9', '#A0C29B', '#FAFA64', '#FB8C32', '#E80F14')
sbr$rng<-"<20"
sbr$rng[sbr$sbr>=20 & sbr$sbr<40]<-"20-40"
sbr$rng[sbr$sbr>=40 & sbr$sbr<60]<-"40-60"
sbr$rng[sbr$sbr>=60 & sbr$sbr<80]<-"60-80"
sbr$rng[sbr$sbr>=80]<-">80"
sbr$rng<-factor(sbr$rng, levels = c("<20","20-40",
                                    "40-60","60-80",">80"))
fig+
  geom_tile(data=sbr, aes(x=lng, y=lat, fill=rng)) +
  xlab("")+ ylab("") +
  scale_fill_manual(name="Breeding suitability",
                    values = pal)+
  theme(legend.position="bottom")
```

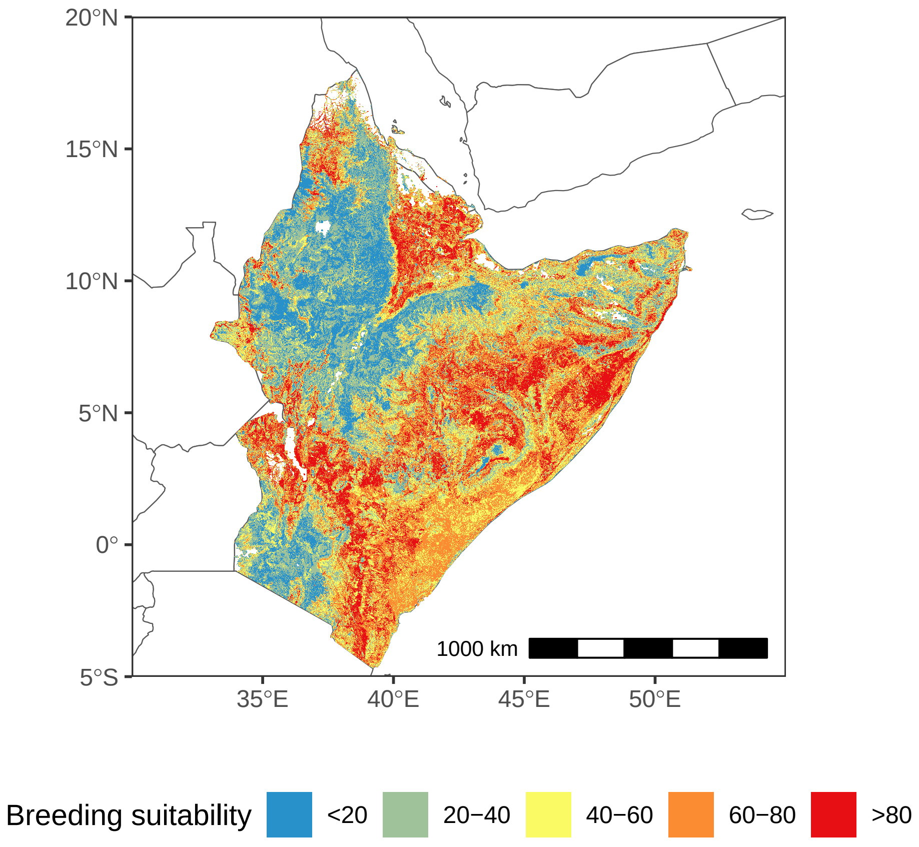


Figure C: Breeding suitability map. The breeding suitability values were adjusted to a 0-100 scale. The base layer of the map is sourced from Natural Earth (https://www.naturalearthdata.com).

Next we check if environmental conditions are suitable for egg laying. For this we need precipitation and soil moisture 24-48 hours before.

```
dt0<-date.eggL
xx<-get_prec_soilm(dt0, lng, lat)
ggplot(xx, aes(x=time, y=value))+
  geom_path(col='darkblue')+
  facet_grid(type~., scales = 'free_y')
```

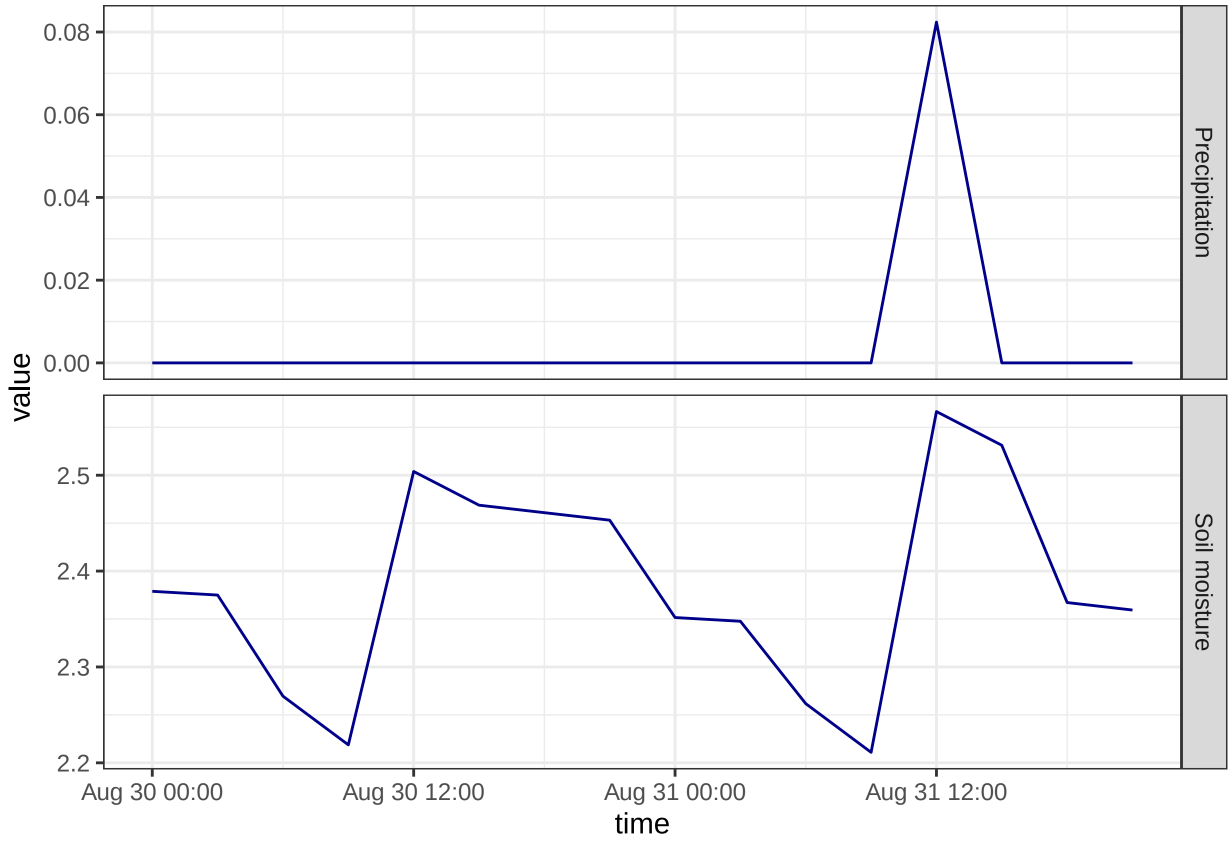


Figure D: Precipitation and soil moisture up to 48 hours before egg laying.

The code returns a vector with:

- Probability that site is suitable for breeding.
- Max precipitation 24-48 hours before.
- Max soil moisture 24-48 hours before.

```
stbbreed<-suit_breeding(dt0, lng, lat)
print(stbbreed)
```

```
## [1] 0.65663728 0.08239746 2.56640601
```

## Egg laying submodel

This sub-model tests if location and environmental conditions are suitable for egg laying.

```
stb.egg.dvlp<-0
set.seed(rn.sd.1)
# Test if site suitable for breeding and recent precipitation event
if(runif(1)<stbbreed[1] & stbbreed[2]>0) stb.egg.dvlp<-1
```

```r
# Or test if site suitable for breeding and soil moisture above the threshold
if(runif(1)<stbbreed[1] & stbbreed[3]>12) stb.egg.dvlp<-1
if( stb.egg.dvlp==1) {
  print("Egg laying succesful")
  BRD<-rbind(BRD, data.frame(ind=ind, lng=lng, lat=lat, date=dt0))
}
```

```
## [1] "Egg laying succesful"
```

### Egg development submodel

This sub-model calculates egg incubation period based on temperature:

```r
date<-date.eggL
lng<-coord.eggL$lng
lat<-coord.eggL$lat
xx<-temp_profile_egg_dev(date, lng, lat)
xx$type<-"Temperature"
dvlp_rt<-egg_development(xx$value)*3/24 # 3-hour temperature data
cm_dvlp_rt<-cumsum(dvlp_rt)
wh<-which(cm_dvlp_rt>=100)
wh<-min(wh)
ans<-rbind(xx[1:wh,],
           data.frame(time=xx$time[1:wh], value=cm_dvlp_rt[1:wh], type="Development %"))

ggplot(ans, aes(x=time, y=value))+
  geom_path(col='darkgray')+
  facet_grid(factor(type, levels=c("Temperature", "Development %"))~., scales = 'free_y')
```

Next, the sub-model tests if environmental conditions are suitable for egg development. The code returns if eggs will develop and hatching date:

```r
  eggdevday<-egg_dev(dt0, lng, lat, 10, 50)
if(is.Date(eggdevday)) {
  print(paste0("Length of egg developmenet: ", as.numeric(eggdevday-dt0), " days."))
  print(paste0("Hoppers hatching on ", eggdevday))
}
```

```
## [1] "Length of egg developmenet: 18 days."
## [1] "Hoppers hatching on 2020-09-19"
```

### Hopper development sub-model

This sub-model calculates length required for hopper development based on temperature:

```r
date<-eggdevday
lng<-coord.eggL$lng
lat<-coord.eggL$lat
xx<-temp_profile_hopper_dev_dur(date, lng, lat)
xx$type<-"Temperature"
dvlp_rt<-hopper_development(xx$value)*3/24 # 3-hour temperature data
cm_dvlp_rt<-cumsum(dvlp_rt)
wh<-which(cm_dvlp_rt>=100)
wh<-min(wh)
ans<-rbind(xx[1:wh,],
           data.frame(time=xx$time[1:wh], value=cm_dvlp_rt[1:wh], type="Development %"))
```

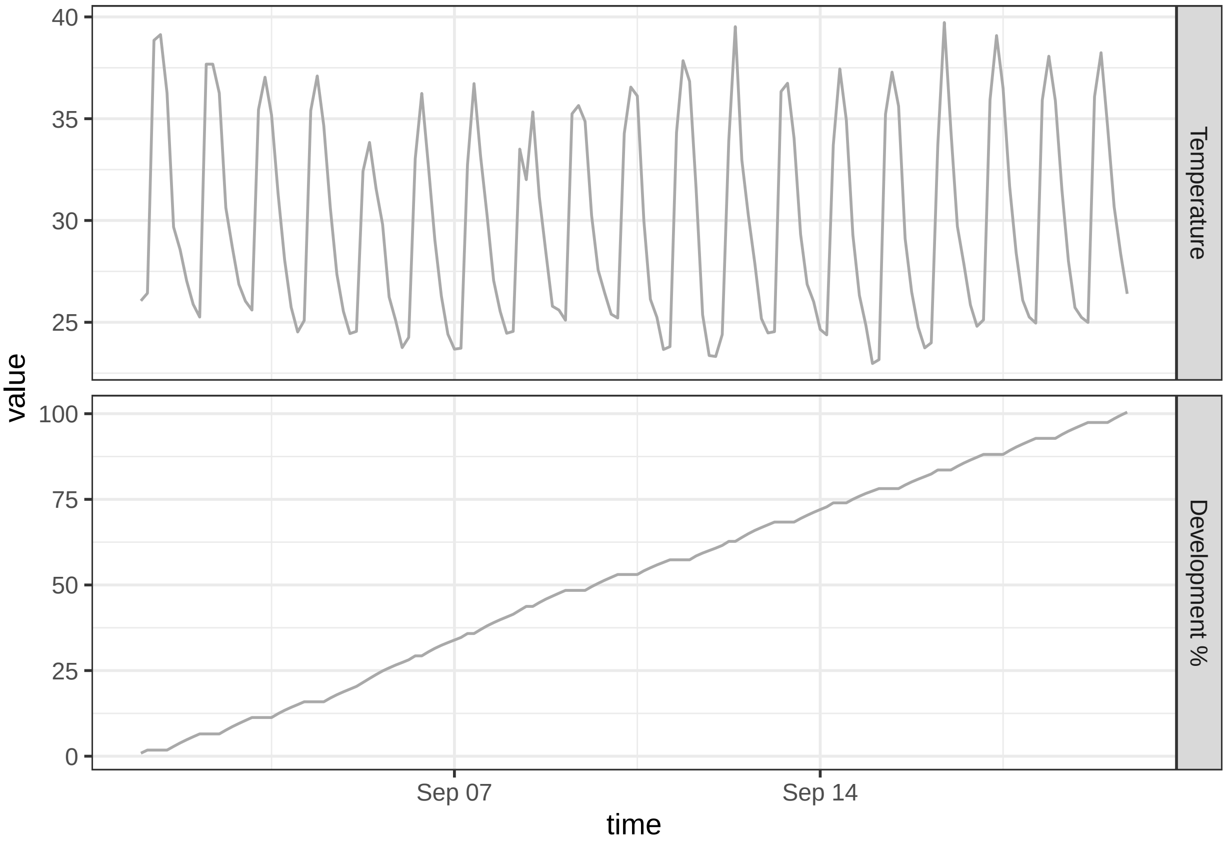


Figure E: An example of temperature and corresponding accumulation of development rate of eggs.

```
ggplot(ans, aes(x=time, y=value))+
  geom_path(col='darkgray')+
  facet_grid(factor(type, levels=c("Temperature", "Development %"))~., scales = 'free_y')
```

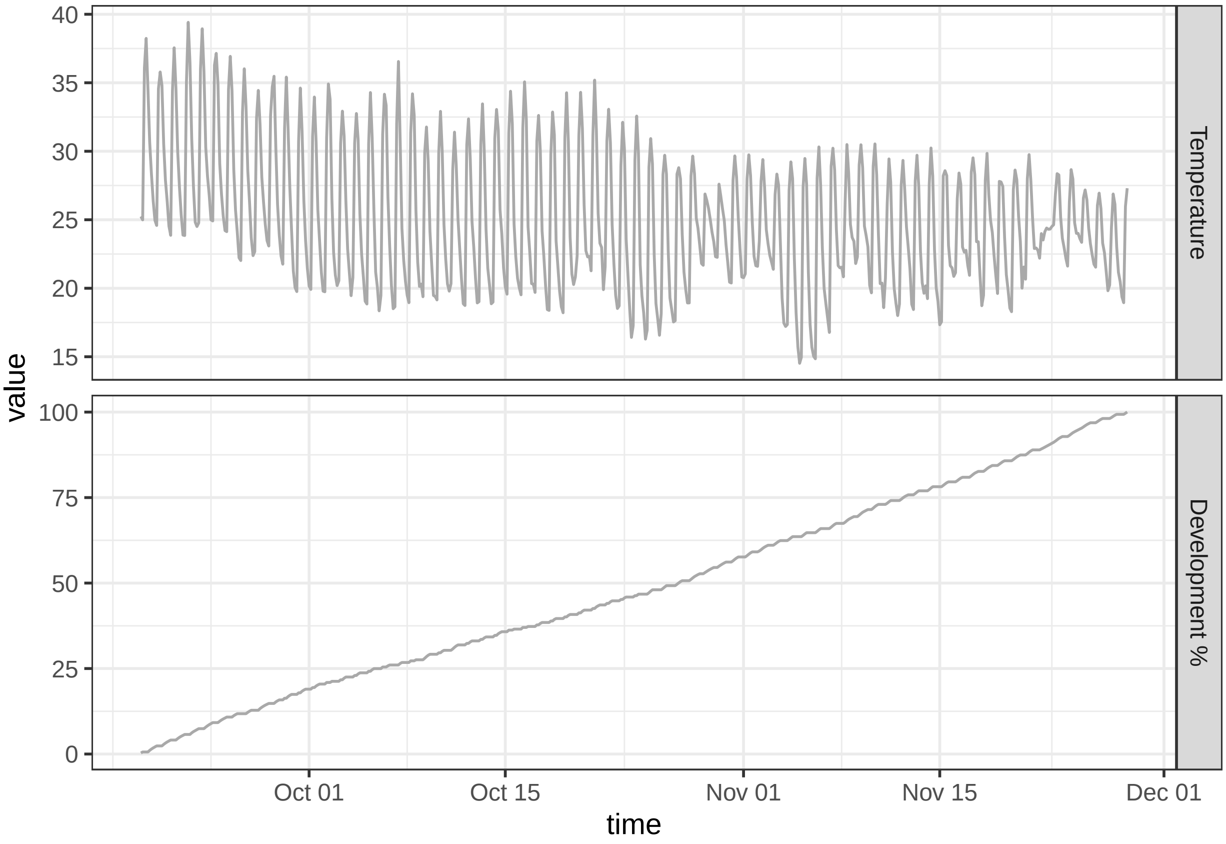


Figure F: An example of temperature and corresponding accumulation of rate development rate of hoppers.

Sub-model tests if conditions in terms of available vegetation, are suitable.

```
dt1<-eggdevday
htoia<-hopper_dev(dt1, lng, lat, 0.09, 24, 95) # Threshold for NDVI, limits for development period
ndvi<-get_NDVI_values(lng, lat, eggdevday, htoia)
yy<-smooth_Whittaker(ndvi$NDVI, lambda=1)
ndvi$NDVIsmooth<-yy
ggplot()+
  geom_path(data=ndvi, aes(x=date, y=NDVI), col='darkgreen') +
  geom_path(data=ndvi, aes(x=date, y=NDVIsmooth), col='red')
```

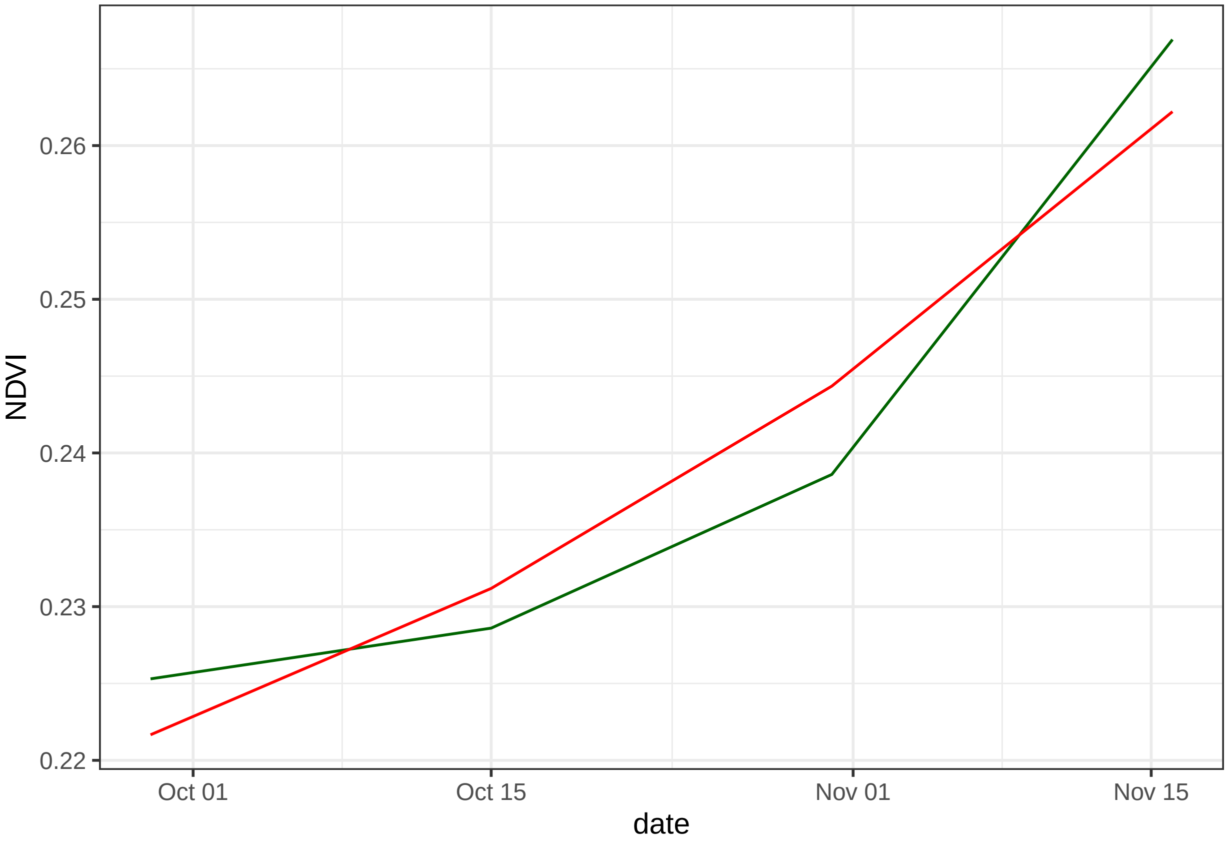


We can see above that both conditions for succesfull hopper development were satisfied, i.e. NDVI was above 0.09 and it was increasing. The code returns date when hoppers will develop into adults:

```
if(is.Date(htoia)) {
    print(paste0("Length of hopper developmenet: ", as.numeric(htoia-eggdevday), " days."))
  print(paste0("Adults appear on ", htoia))
  HPRS<-rbind(HPRS, data.frame(ind=ind, lng=lng, lat=lat, dateS=dt1, dateF=htoia))
}
```

```
## [1] "Length of hopper developmenet: 70 days."
## [1] "Adults appear on 2020-11-28"
```

### Dispersal of swarms

The final step is migration of swarms, which involves testing how long vegetation available on a ground can sustain a swarm and swarms following prevailing winds.

On the first migration day, figure below shows distribution of wind trajectories from the location of breeding and chosen wind trajectory in red.

```
date<-htoia
xx<-data.frame(date =c(), lng=c(), lat=c(), flt.tm=c(), ind=c())
for(k in 1:1000){
    day.trj<-get_wind_ind(date, lng, lat, k)
    tm<-as.numeric(day.trj$date-min(day.trj$date))/(60*60)
    day.trj$flt.tm<-tm
    day.trj$ind<-k
    xx<-rbind(xx, day.trj)
}
set.seed(rn.sd.1)
```

```
j<-sample(1:1000,1)

ggplot(xx, aes(x=lng, y=lat)) +
  geom_path(aes(group=ind, col=flt.tm)) +
  theme(legend.position = "none") +
  xlab("") + ylab("") +
  scale_color_viridis(option = "D", direction=-1)+
  theme(panel.grid.major = element_blank(), panel.grid.minor = element_blank(),
        panel.background = element_blank()) +
  geom_path(data=xx[xx$ind==j,], aes(x=lng, y=lat), col='red') +
  coord_fixed()
```

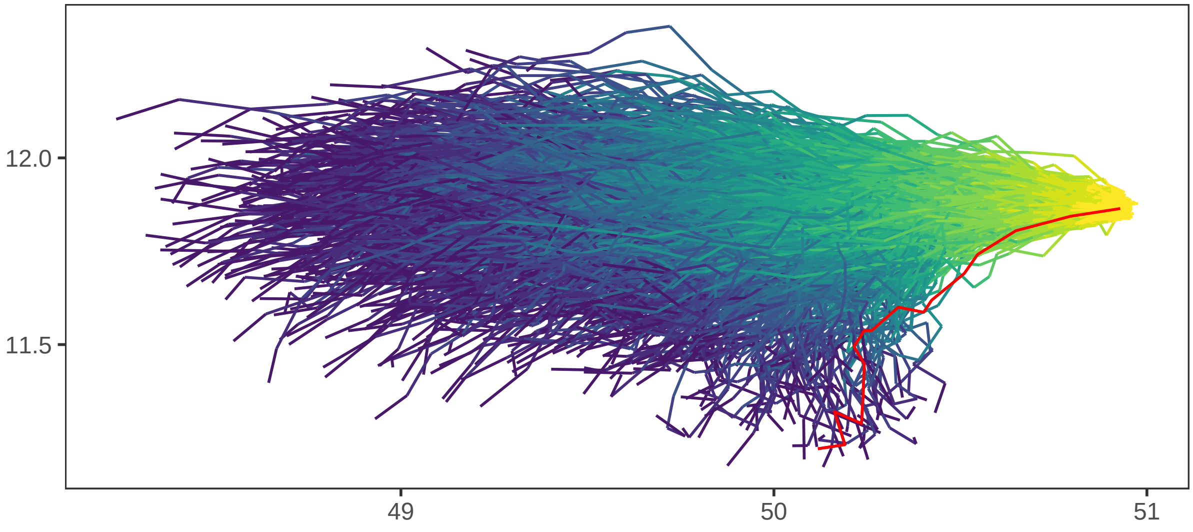


Figure G: An example of NAME wind trajectories on the 15th of January 2021. The colour shows the difference in hours between points on a trajectory and the initial point.

The duration of swarm feeding at a single location depends on the state and availability of vegetation for feeding;

```
ggplot(lng.stay[!is.na(lng.stay$stay),], aes(x=tr, y=vl)) +
  geom_tile(aes(fill=stay)) +
  facet_grid(.~lc) +
  scale_fill_manual(name = "Duration",
                    values=c("#7570b3", "#1b9e77", "#d95f02"))+
  xlab("NDVI trend")+ ylab("NDVI value")+
  theme(axis.text.x = element_text(angle = 90, vjust = 0.5, hjust=1))
```

Check land cover type:

```
day.trj<-xx[xx$ind==j,]
wh<-which(day.trj$flt.tm==max(day.trj$flt.tm))
lng<-day.trj$lng[wh]
lat<-day.trj$lat[wh]
lct<-get_land_cover(lng, lat)
ndvi<-get_NDVI_values(lng, lat, date-150, date)
yy<-smooth_Whittaker(ndvi$NDVI, lambda=1)
ndvi$NDVIsmooth<-yy
a<-swrm_stay(lct, ndvi, 6, rn.sd.2)
```

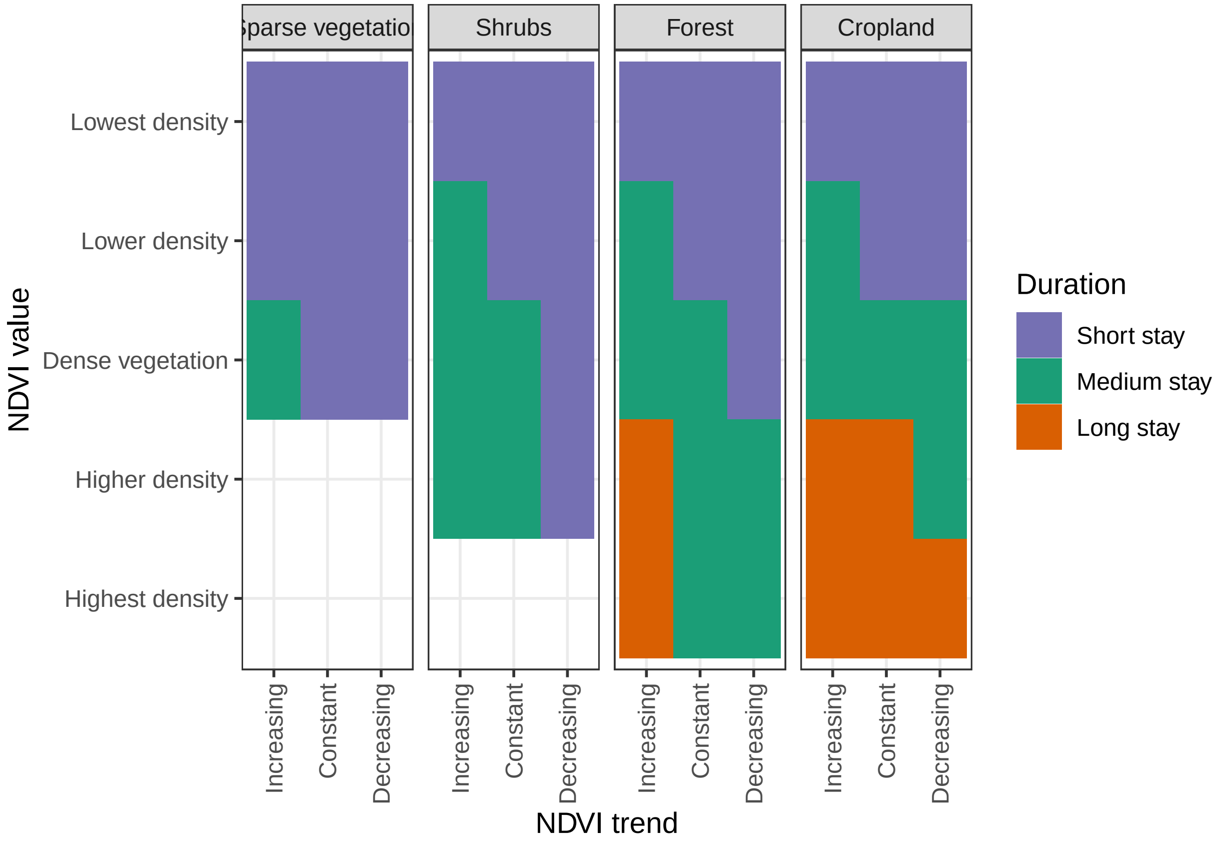


Figure H: Length of stay for swarms depends on land cover type, NDVI trend (increasing, decreasing or constant) and vegetation state (value of NDVI). Here short stay is 1-2 days, medium stay is 2-4 days, and long stay is 4-7 days.

```r
print(paste0("Land cover type: ", get_lct(lct)))
```

```
## [1] "Land cover type: Shrubs"
```

```r
print(swrm_stay_type(lct, ndvi, 6))
```

```
## [1] "Short stay"
```

```r
print(paste0("Staying for ", a, " days."))
```

```
## [1] "Staying for 2 days."
```

```r
print(paste0("Date ", date))
```

```
## [1] "Date 2020-11-28"
```

Next we check status of vegetation. Values of raw NDVI (green) and smoothed NDVI (red) are shown below:

```r
ggplot()+
  geom_path(data=ndvi, aes(x=date, y=NDVI), col='darkgreen') +
  geom_path(data=ndvi, aes(x=date, y=NDVIsmooth), col='red')
```

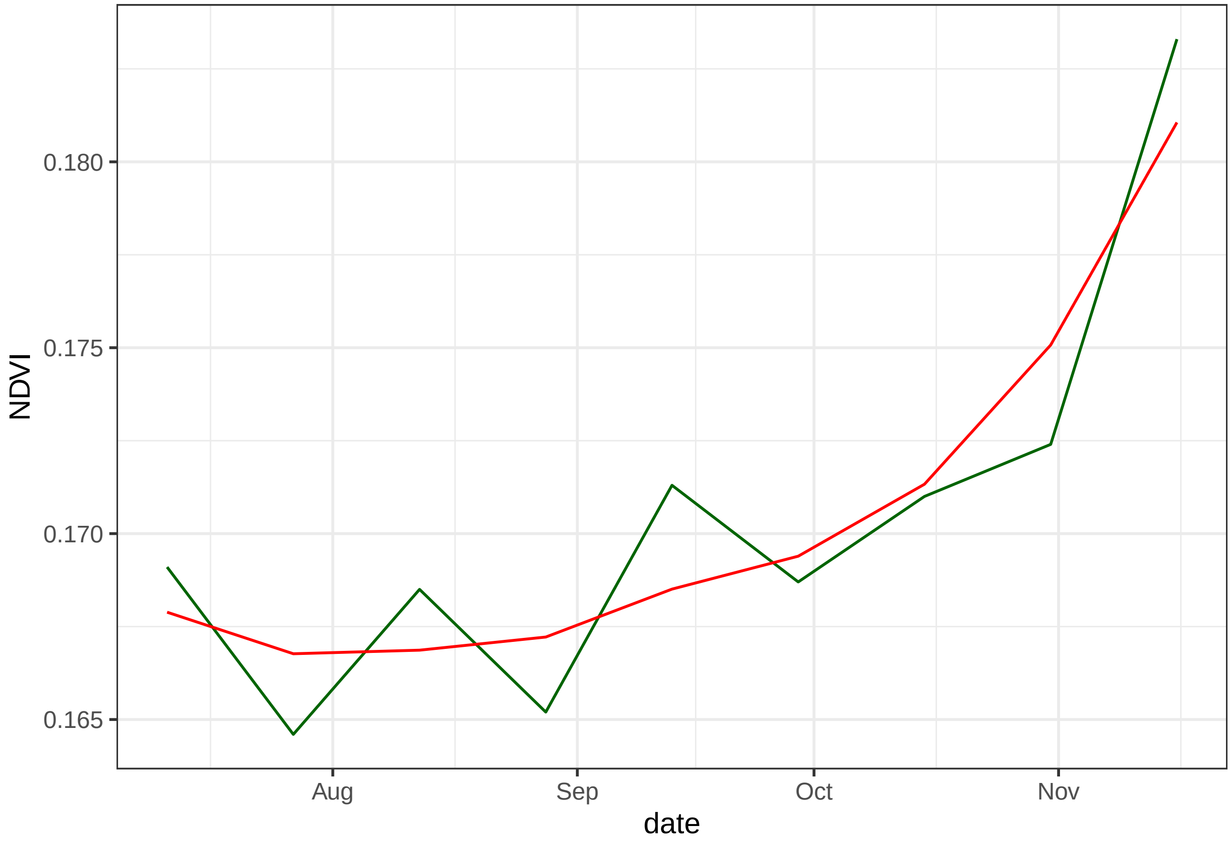


Figure I: Raw and smoothed NDVI profile from July to December 2020.

The code returns how long a swarm will stay at the landing site:

```r
a<-swrm_stay(lct, ndvi, 3, rn.sd.2)
print(paste0("Land cover type: ", get_lct(lct)))
```

```
## [1] "Land cover type: Shrubs"
```

```
print(swrm_stay_type(lct, ndvi, 6))
```

```
## [1] "Short stay"
```

```
print(paste0("Staying for ", a, " days."))
```

```
## [1] "Staying for 2 days."
```

Run simulations till 15th of January 2021.

```
    date<-htoia
    lng<-coord.eggL$lng
    lat<-coord.eggL$lat
      day.trj<-get_wind(date, lng, lat, rn.sd.1)
      tm<-as.numeric(day.trj$date-min(day.trj$date))/(60*60)
      day.trj$flt.tm<-tm
      day.trj$ind<-ind
      TRJ<-rbind(TRJ, day.trj)
      dd<-1
      while(dd<100){ # Follow for 100 days or up to 15th of January
        wh<-which(day.trj$flt.tm==max(day.trj$flt.tm))
        lng<-day.trj$lng[wh]
        lat<-day.trj$lat[wh]
        lct<-get_land_cover(lng, lat)
        ndvi<-get_NDVI_values(lng, lat, date-150, date)
        # Continue simulations only if staying within study area
        if(is.na(ndvi$NDVI[1])){
          dd<-100
        } else {
          if(sum(ndvi$NDVI)==0){
            dd<-100
          } else {
            dd<-dd+1
            yy<-smooth_Whittaker(ndvi$NDVI, lambda=1)
            ndvi$NDVIsmooth<-yy
            a<-swrm_stay(lct, ndvi, 6, rn.sd.2)
            ST.DAYS<-rbind(ST.DAYS, data.frame(ind=ind, lng=lng,
                                               lat=lat, date=date, stay=a))
            date<-date+1+a
            day.trj<-get_wind(date, lng, lat, rn.sd.1)
            tm<-as.numeric(day.trj$date-min(day.trj$date))/(60*60)
            day.trj$flt.tm<-tm
            day.trj$ind<-ind
            TRJ<-rbind(TRJ, day.trj)
            if(date>=as.Date("2021-01-15")) dd<-100
          }
        }
      }
```

Plot swarm migration pathway together with landing sites and days stayed.

```
ids<-6
fig+
  geom_path(data=TRJ,
            aes(x=lng, y=lat, group=ind, col=date)) +
  geom_point(data=coord.eggL, aes(x=lng, y=lat), col='red', size=1.5)+
```

```
  geom_point(data=ST.DAYS, aes(x=lng, y=lat), fill='white',
             pch=21, size=5) +
    geom_point(data=ST.DAYS[ids,], aes(x=lng, y=lat), fill='lightgreen',
             pch=21, size=5) +
      geom_point(data=ST.DAYS[ids+1,], aes(x=lng, y=lat), fill='purple',
             pch=21, size=5) +
  geom_text(data=ST.DAYS, aes(x=lng, y=lat, label=stay), size=3)
```

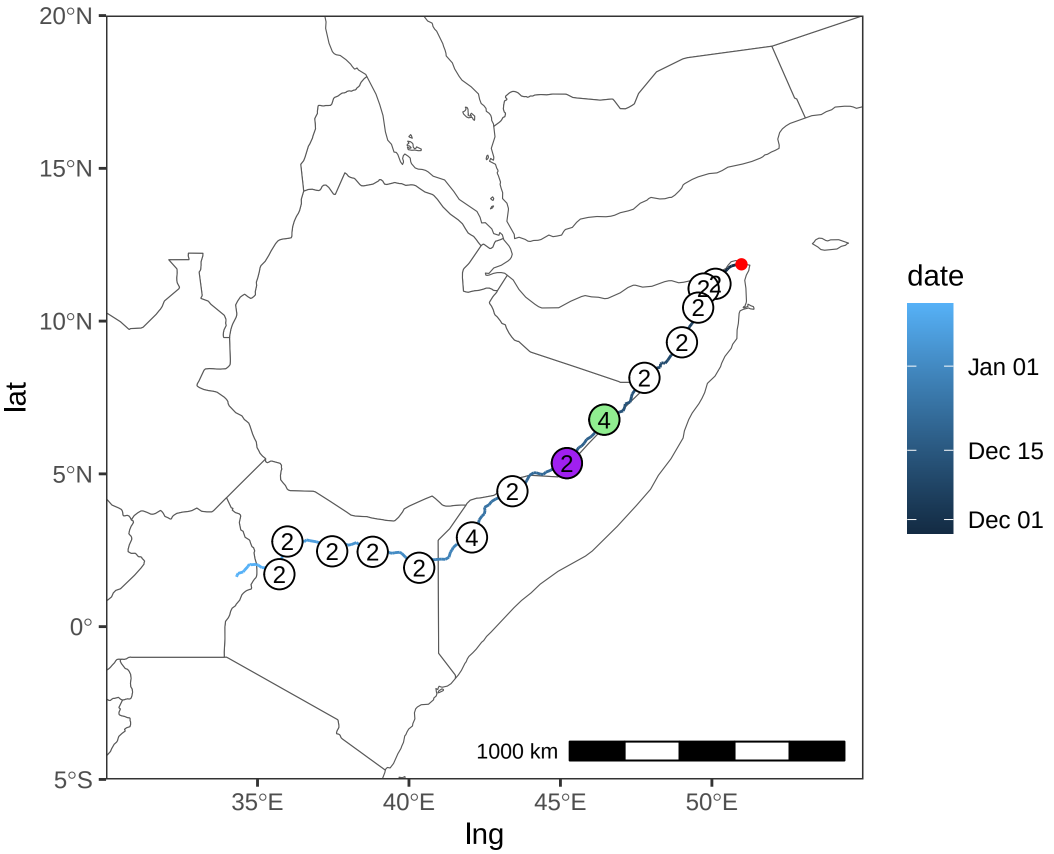


For illustration, we will look in more details what happened at and between the landing sites shown above in green and purple.

```
lng<-ST.DAYS$lng[ids]
lat<-ST.DAYS$lat[ids]
date<-ST.DAYS$date[ids]
lct<-get_land_cover(lng, lat)
ndvi<-get_NDVI_values(lng, lat, date-150, date)
yy<-smooth_Whittaker(ndvi$NDVI, lambda=1)
ndvi$NDVIsmooth<-yy
a<-swrm_stay(lct, ndvi, 6, rn.sd.2)
print(paste0("Land cover type: ", get_lct(lct)))
```

```
## [1] "Land cover type: Shrubs"
```

```
print(swrm_stay_type(lct, ndvi, 6))
```

```
## [1] "Medium stay"
```

```
print(paste0("Staying for ", a, " days."))
```

```
## [1] "Staying for 4 days."
```

```r
print(paste0("Date ", date))
```

```
## [1] "Date 2020-12-13"
```

Values of raw NDVI (green) and smoothed NDVI (red) are shown below:

```r
ggplot()+
  geom_path(data=ndvi, aes(x=date, y=NDVI), col='darkgreen') +
  geom_path(data=ndvi, aes(x=date, y=NDVIsmooth), col='red')
```

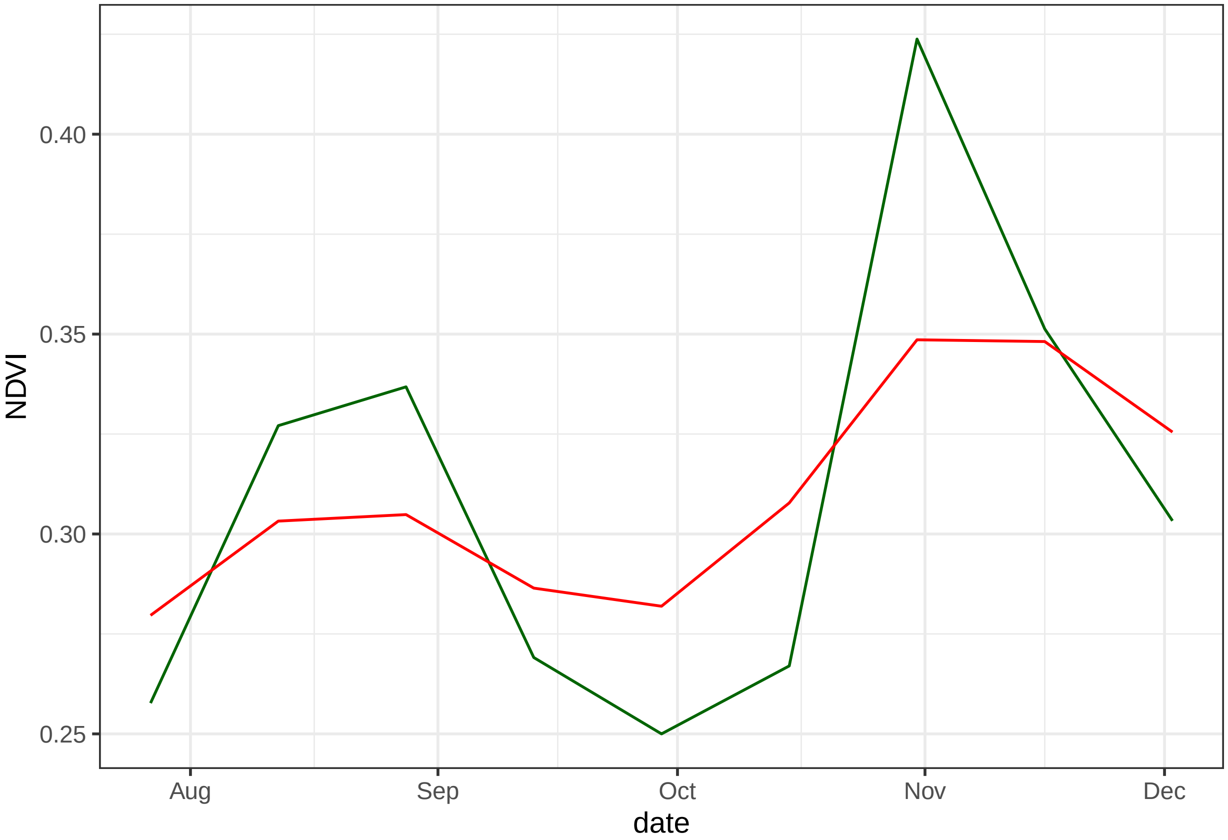


At the first landing site, land cover type was shrubs and a swarms stayed for 4 days.

Figure below shows distribution of wind trajectories from the landing site and chosen wind trajectory in red.

```r
date<-ST.DAYS$date[ids+1]
xx<-data.frame(date =c(), lng=c(), lat=c(), flt.tm=c(), ind=c())
for(k in 1:1000){
    day.trj<-get_wind_ind(date, lng, lat, k)
    tm<-as.numeric(day.trj$date-min(day.trj$date))/(60*60)
    day.trj$flt.tm<-tm
    day.trj$ind<-k
    xx<-rbind(xx, day.trj)
}
set.seed(rn.sd.1)
j<-sample(1:1000,1)

ggplot(xx, aes(x=lng, y=lat)) +
  geom_path(aes(group=ind, col=flt.tm)) +
  theme(legend.position = "none") +
```

```
  xlab("") + ylab("") +
  scale_color_viridis(option = "D", direction=-1)+
  theme(panel.grid.major = element_blank(), panel.grid.minor = element_blank(),
        panel.background = element_blank()) +
  geom_path(data=xx[xx$ind==j,], aes(x=lng, y=lat), col='red') +
  coord_fixed()
```

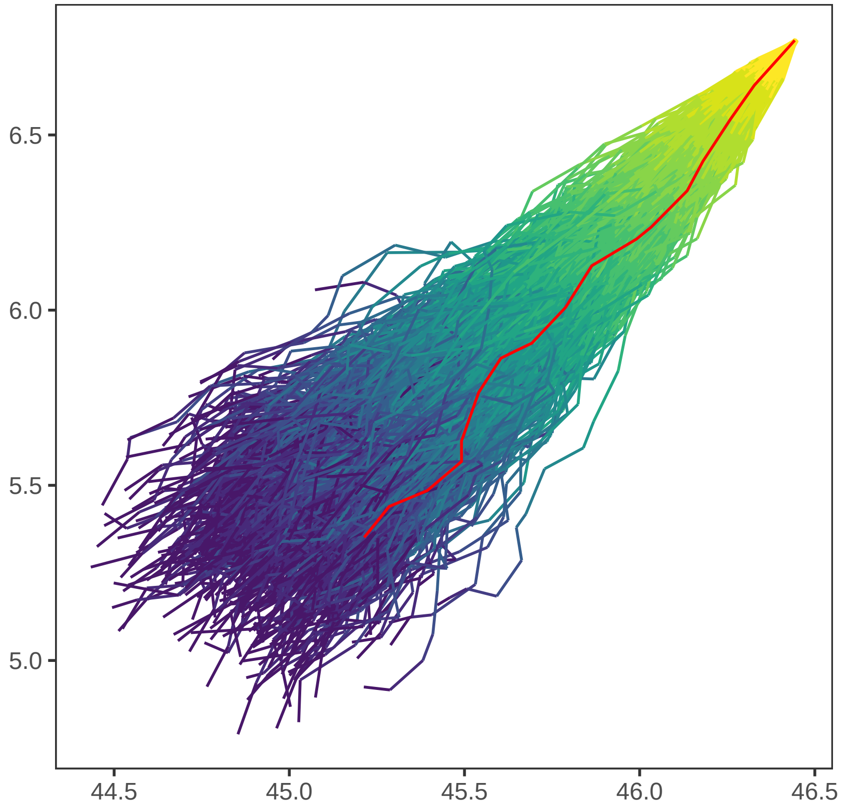


Now for second landing site shown in green:

```
lng<-ST.DAYS$lng[ids+1]
lat<-ST.DAYS$lat[ids+1]
date<-ST.DAYS$date[ids+1]
lct<-get_land_cover(lng, lat)
ndvi<-get_NDVI_values(lng, lat, date-150, date)
yy<-smooth_Whittaker(ndvi$NDVI, lambda=1)
ndvi$NDVIsmooth<-yy
a<-swrm_stay(lct, ndvi, 6, rn.sd.2)
print(paste0("Land cover type: ", get_lct(lct)))
```

```
## [1] "Land cover type: Shrubs"
```

```
print(swrm_stay_type(lct, ndvi, 6))
```

```
## [1] "Short stay"
```

```
print(paste0("Staying for ", a, " days."))
```

```
## [1] "Staying for 2 days."
```

```
print(paste0("Date ", date))
```

```
## [1] "Date 2020-12-18"
```

Values of raw NDVI (green) and smoothed NDVI (red) are shown below:

```
ggplot()+
  geom_path(data=ndvi, aes(x=date, y=NDVI), col='darkgreen') +
  geom_path(data=ndvi, aes(x=date, y=NDVIsmooth), col='red')
```

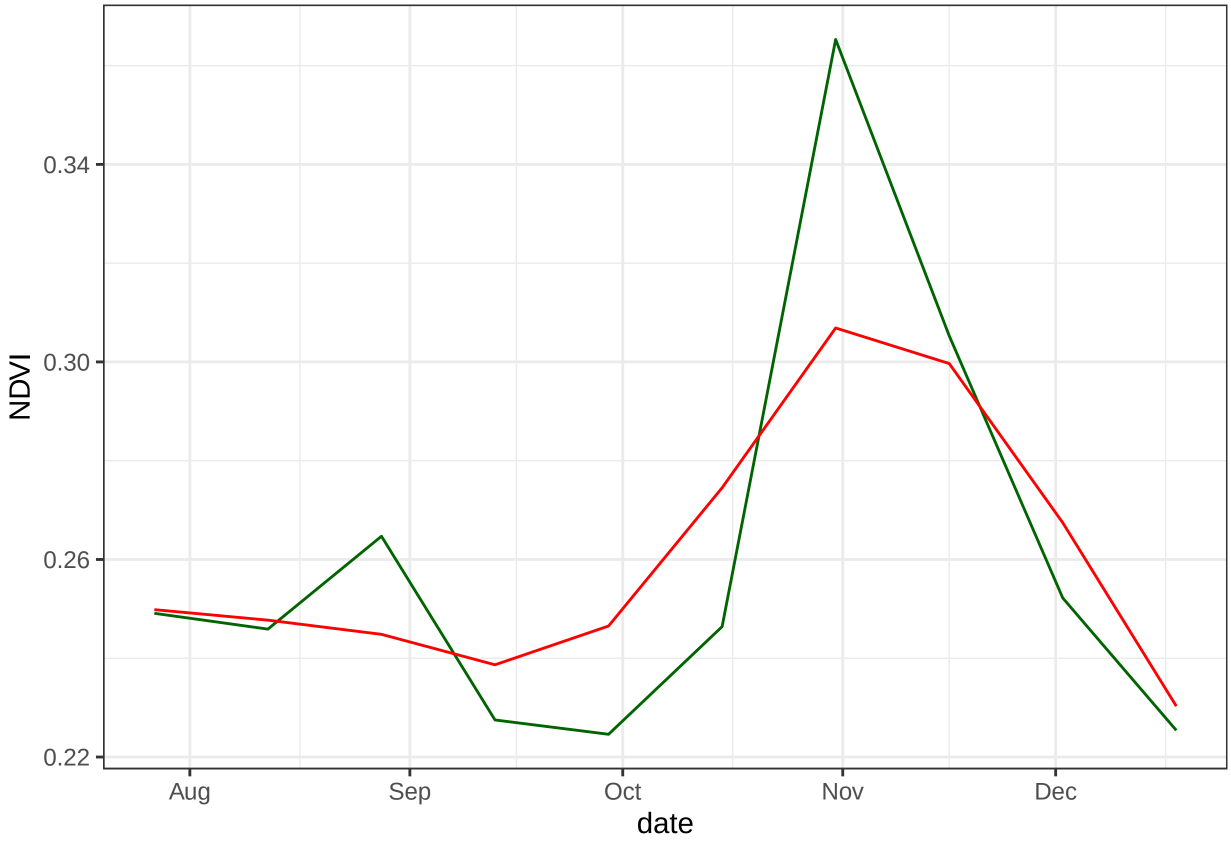


Figure J: Raw and smoothed NDVI profile.
