# Supplementary material for: A framework for modelling desert locust population dynamics and large-scale dispersal
Source: PLoS Comput Biol. 2024 Dec 19;20(12):e1012562. doi: 10.1371/journal.pcbi.1012562 (PMC11658591; doi:10.1371/journal.pcbi.1012562)
Supplement: S1 Appendix — (PDF) [file pcbi.1012562.s002.pdf]

S1 Appendix to: A framework for modelling desert locust population dynamics and large-scale dispersal

Renata Retkute[a], William Thurston[b], Keith Cressman[c], Christopher A. Gilligan[a]

[a]Epidemiology and Modelling Group, Department of Plant Sciences, University of Cambridge, Downing Street, Cambridge, CB2 3EA, UK

[b]Met Office, Fitzroy Road, Exeter, EX1 3PB, United Kingdom

[c]Food and Agriculture Organization of the United Nations, Viale delle Terme di Caracalla, Rome, 00153, Italy

This document provides a detailed description of the essential steps in using the framework as well as additional analysis of the model. The code used in the analyses is available on a GitHub repository at https://github.com/rretkute/PestDynamicsAndDispersal. The data used in the analyses is available via the University of Cambridge Data Repository at https://doi.org/10.17863/CAM.11188.

Footnotes are provided for each reference on the page where the reference is used for readers' convenience. The references are also summarised in a bibliography at the end of the document.

Table of Contents

Table of Contents ***1***

S1. Introduction ***4***

S1.1. Aim of the study **4**

S1.2. Study Area **4**

S1.3. Simplified life cycle of DLs **4**

S1.4. Modelling framework. **5**

S2. Data. ***6***

S2.1. Spatial resolution **6**

S2.2. Desert locust data **6**

S2.2.1. Data used for breeding suitability mapping 7

S2.2.2. Data used for evaluation of swarm movement 7

S2.3. Elevation data **8**

S2.4. Sand and clay content in the soil **9**

S2.5. Land cover data 9
S2.6. MODIS NDVI data 10
S2.7. The Met Office NAME model: temperature, soil moisture and precipitation 11
S2.8. The Met Office NAME model: wind trajectories 12
S3. NDVI data analysis *13*
S3.1. Smoothing of NDVI data 13
S3.2. Assessing NDVI trend 14
S3.3. Finding peaks in a NDVI time serries 15
S4. Finding areas suitable for breeding *16*
S4.1. Desert locust data and environmental covariates 16
S4.2. Machine learning approach to map potential breeding sites 16
S4.3. Running the breeding suitability prediction code 17
S4.4. Choosing the number of decision trees 17
S4.5. Predicted breeding suitability across the domain. 17
S4.6. Evaluating performance of the classifier 18
S4.7. Comparison with published maps 19
S5. Egg laying *19*
S5.1. Testing if area is suitable for egg laying. 20
S5.2. Testing if conditions are suitable for egg laying. 20
S6. Development from eggs to hoppers *21*
S6.1. Time required for eggs to develop. 21
S6.2. An example of egg development period distribution. 21
S7. Development from hoppers to adults *22*
S7.1. Time required for hoppers to develop into adults. 22
S7.2. Analysis of hopper development period. 23
S7.3. Conditions required for hoppers to develop. 24
S8. Estimating food availability for migrating swarms *24*
S8.1. Food requirement for an average swarm 24
S8.2. Relationship between land cover type, NDVI, and aboveground biomass 24
S8.3. Relationship between land cover types and NDVI density 26
S8.4. Feeding period 26
S8.5. Spatial distribution of feeding period 27
S9. Estimating time periods suitable for migration *28*
S9.1. Calculating wind direction angle 28
S9.2. Temporal distribution of wind direction 28

S9.3. Fitting the von Mises distribution **29**

S10. Predicting day to day movement of swarms ***30***

S10.1. Simulating day to day movement of swarms. **30**

S10.2. Short term prediction of swarm migration **30**

S11. Simulating breeding, development and movement of DLs ***32***

S11.1. Model simulation from a specific breeding site **32**

S11.2. Model simulation from an extended breeding area in north east Somalia **33**

S11.3. Model simulation from an extended region in northern Somalia **34**

S12. Tutorial ***35***

S1. Introduction

S1.1. Aim of the study

The principal aim of the work is to provide an integrated modelling framework that can be used as a practical starting point for use in the next desert locust (DL) upsurge to inform surveillance and control. Specifically, we address the following topics:

- construction of a modelling framework that integrates DL breeding, development through egg, hopper and adult stages, feeding and swarm migration;
- characterisation of breeding sites using a combination of site-specific static and dynamic variables to predict egg-laying, egg, hopper and adult development leading to DL swarming;
- incorporation of weather-driven models for wind trajectories to predict daily pathways of swarm migration;
- use of remote-sensed data to predict the duration of swarm feeding at a single location given the state and availability of vegetation for feeding;

S1.2. Study Area

We consider a domain extending across five sub-Saharan countries (Kenya, Ethiopia, Somalia, Eritrea and Djibouti) that were affected by the 2019-21 DL upsurge. In accordance with our study area, our simulations were restricted to longitude 30^0E to 55^0E and latitude between 5^0S to 20^0N (Figure A). The domain can be exchanged for other domains, providing appropriate data are available for wind trajectories and other variables described below.

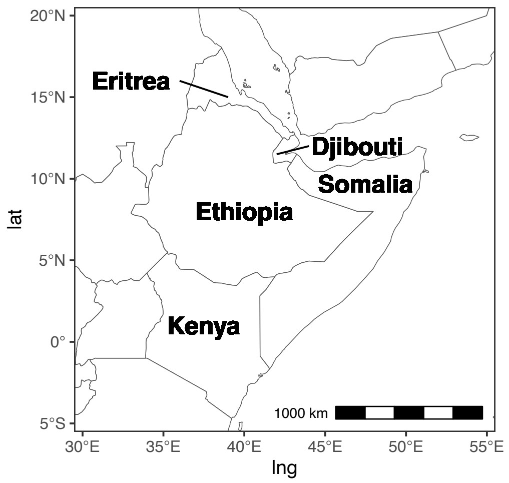

Figure A. Study area. *The base layer of the map is sourced from Natural Earth (https://www.naturalearthdata.com).*

S1.3. Simplified life cycle of DLs

We assumed that DL populations can be in one of three non-overlapping life-stages, also called compartments: eggs, hoppers (nymphs), and adults (Figure B). Transitions between compartments have different ecological requirements and the relative success of a generation of DLs moving into a new compartment responds differently to environmental

conditions, such as weather, soil, vegetation, and prevailing winds[1]. Below we describe all components of the modelling framework and how environmental, climatological and meteorological data are integrated within the framework.

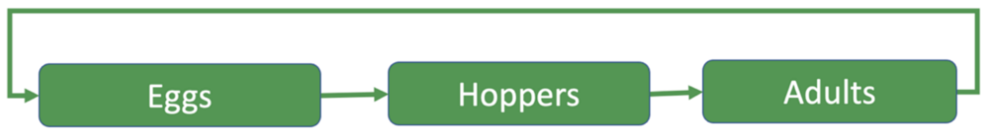

Figure B. *Simplified life cycle of desert locust.*

S1.4. Modelling framework.

The principal aim of the work is to provide a modelling framework that integrates DL breeding, development through egg, hopper and adult stages, feeding and swarm migration with remote-sensed data and weather-driven models for wind trajectories. Data required for the model components are described in Section S2. Pre-processing of normalized difference vegetation index (NDVI) values is described in Section S3.

A schematic of the modelling framework is shown in Figure C. The first step in modelling is to identify areas suitable for breeding (Section S6). Simulation starts with choosing a location and date for breeding. Step two checks if location and environmental conditions are suitable for egg laying (see also Section S6). If eggs are successfully deposited, then the model proceeds to step three. For this, the egg incubation period is calculated based on the temperature at the location, and if the period is within a biologically viable range, we assume that hoppers emerge (Section S7). Step four calculates the length required for hopper development and testing if conditions in terms of available vegetation, are suitable (Section S8). The final step is migration of swarms, which involves testing how long vegetation is available at a landing site to sustain a swarm (Section S9) before swarms migrate aided by prevailing winds (Section S10). We finish with a tutorial for an example simulation (Section S11).

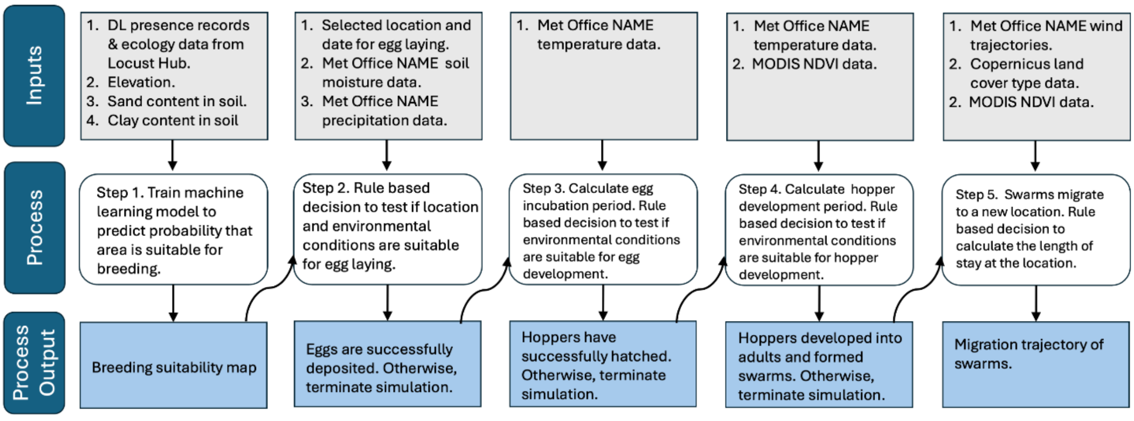

[1] http://www.fao.org/ag/locusts/common/ecg/359/en/SOPBiologyENv2021.pdf

Figure C. *Schematics of the modelling framework.*

S2. Data.

In this section we describe DL, remote sensing and meteorological data used for the modelling framework.

S2.1. Spatial resolution

We chose a spatial resolution of the modelling framework to be 1 km x 1 km (Figure D). The framework could be formulated at any required spatial resolution, but we deemed that 1 km is suitable for the biological and migratory processes under consideration. As data for different variables were available at various spatial resolutions, they were upscaled or downscaled to the spatial resolution of 1 km. The exception was the data used for breeding suitability (elevation, sand and clay content in the soil), which were used at their native resolution to perform calculations on the Google Earth Engine platform.

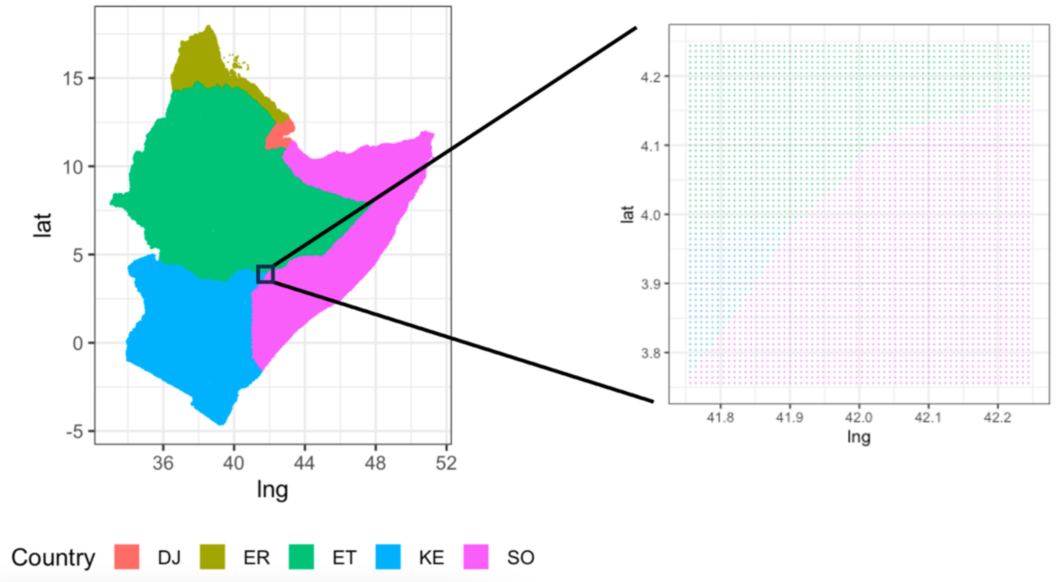

Figure D. *Model is defined on a regular 1 km x 1 km grid. Colours indicate countries. The base layer of the map is sourced from Natural Earth (https://www.naturalearthdata.com).*

Code for spatial resolution is available in file '2_1 Spatial resolution.R'.

S2.2. Desert locust data

We downloaded data from the FAO Locust Hub[2]. The repository has data assigned to the following classes: 'Hoppers', 'Adults', 'Bands', 'Swarms', 'Ecology' and 'Control'. Class 'Ecology' contains records on ecological conditions at all visited sites irrespective of DL presence or absence. The classes 'Hoppers' and 'Adults' contain data on solitarious DLs. Gregarious hoppers are assigned to the 'Bands' class, and gregarious adults are assigned to

the ‘Swarms’ class. We extracted all records dated after 1st January 2010 with locations within the study area.

S2.2.1. Data used for breeding suitability mapping

We aggregated hopper and band occurrence records and denoted these as ‘presence data’. Data for the ‘Ecology’ class that were used to infer locations in which active surveys never found locusts.

We cross-referenced locations in the ‘Ecology’ class that had no reports of hoppers or bands within a 1 km radius and assigned these entries to ‘absence data’. In total, there were 13,390 locations in which hoppers/bands were present and 6,578 locations in which hopper/bands were absent. The distributions of presence and absence data are shown in Figure E.

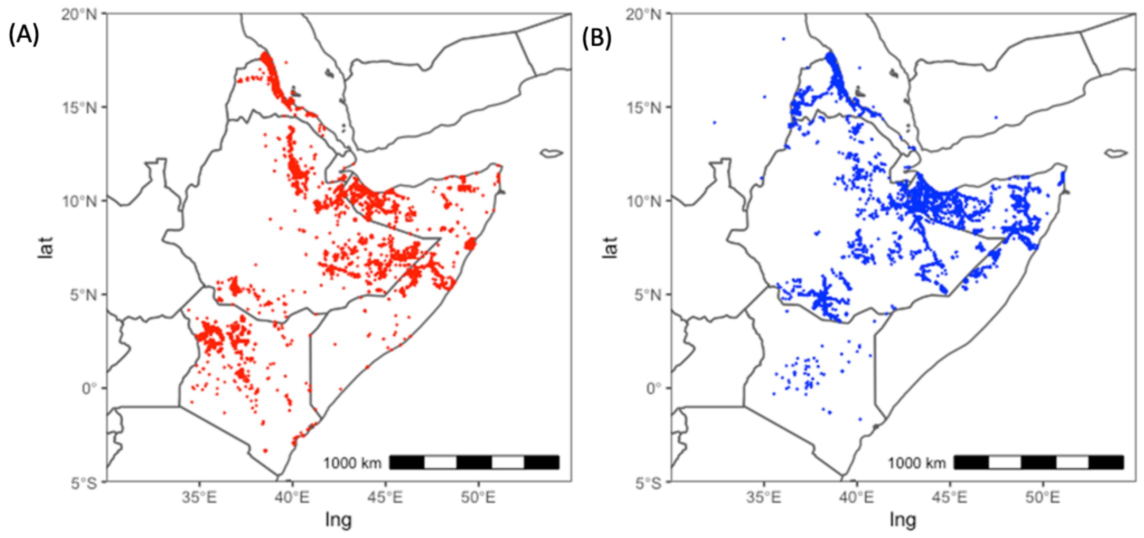

Figure E. *(A) Locations of hopper/band presence records. (B) Locations of hopper/band absence records. The base layer of the map is sourced from Natural Earth (https://www.naturalearthdata.com).*

Code for presence/absence data is available in file ‘2_2_1 Desert locust data absence presence.R’.

S2.2.2. Data used for evaluation of swarm movement

We extracted data for two time periods. The first period (1st September - 1st October 2020) corresponds with the egg laying time[2], and the second period (1st December 2020 - 15th January 2021) corresponds with the observed migration of DL from north Somalia and Ethiopia to Kenya[3]. The locations of reported swarms for the two time periods are shown in Figure F.

---

[2] Desert Locust briefs 2020, https://www.fao.org/ag/locusts/en/archives/briefs/2515/2516/index.html
[3] Desert Locust briefs 2021, https://www.fao.org/ag/locusts/en/archives/briefs/2515/2568/index.html

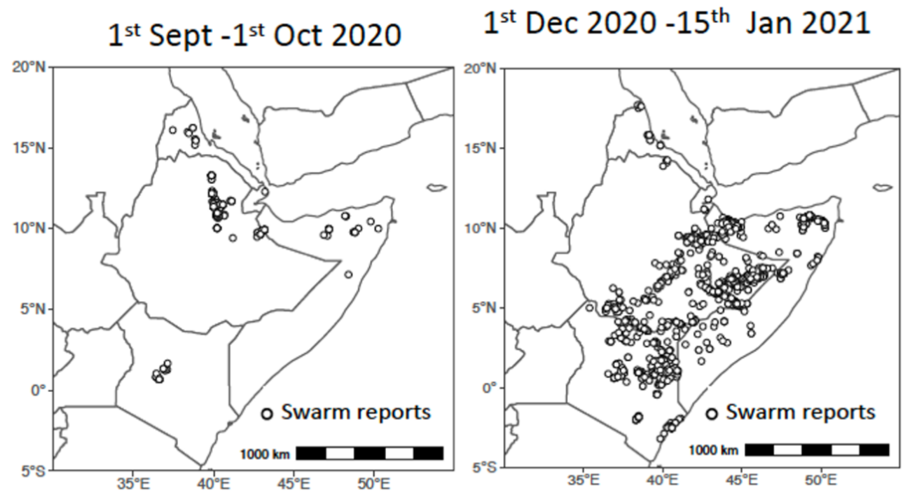

Figure F. *Locations of reported swarms. The base layer of the map is sourced from Natural Earth (https://www.naturalearthdata.com).*

S2.3. Elevation data

We used the Shuttle Radar Topography Mission (SRTM) digital elevation dataset[4] from the collection provided on Google Earth Engine platform ("CGIAR/SRTM90_V4"). The resolution of the dataset is 90 m. Figure G shows a map of elevation in the study area.

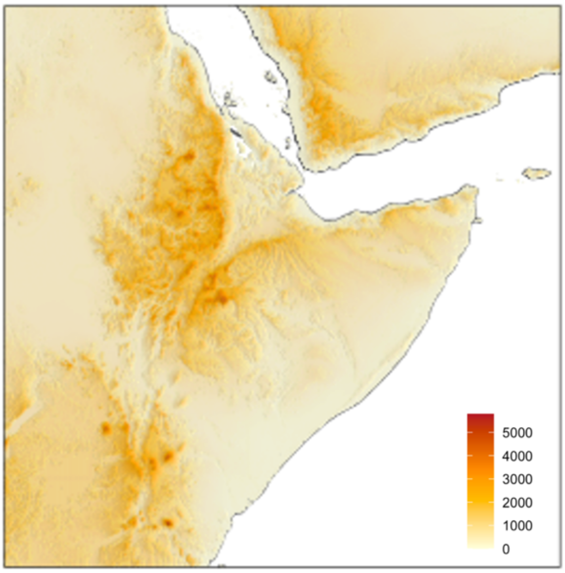

Figure G. *Elevation data (m). The base layer of the map is sourced from Natural Earth (https://www.naturalearthdata.com).*

[4] https://srtm.csi.cgiar.org

S2.4. Sand and clay content in the soil

We used sand content and clay content in the soil at 5 cm depth from the International Soil Reference and Information Centre (ISRIC) soil grids dataset[5]. We downloaded geotiff files for the African continent[6]. Geotiff files were uploaded to the Google Earth Engine platform as covariates for breeding suitability analysis. Resolution of the data was 250 m. Figure H shows the distribution of sand and clay content in the study area.

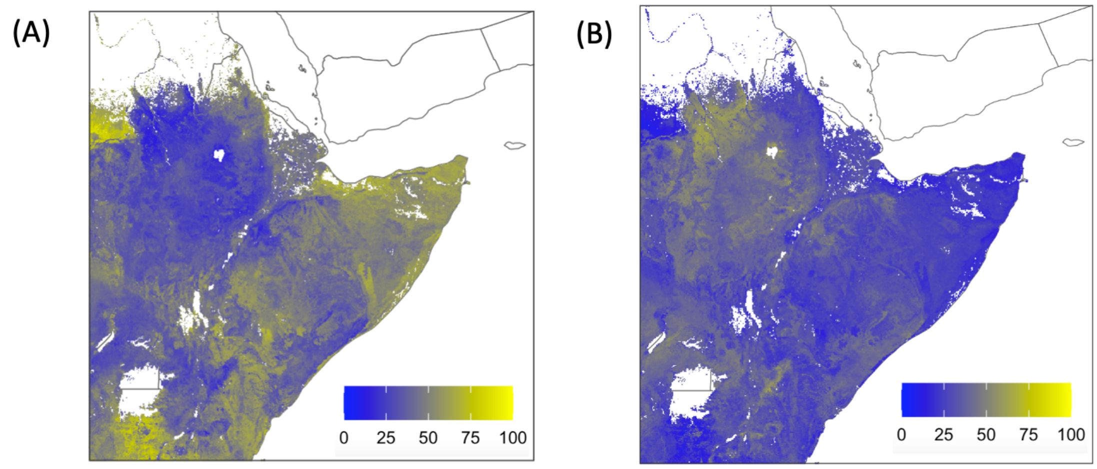

Figure H. *(A) Sand content in the soil; and (B) clay content in the soil. Values are proportion of sand particles (> 0.05 mm) and proportion of clay particles (< 0.002 mm) in the fine earth fraction (g/kg). The base layer of the map is sourced from Natural Earth (https://www.naturalearthdata.com).*

S2.5. Land cover data

We extracted data for land cover classification from the Copernicus global map of land cover (CLC100)[7]. Resolution of the data was 100m. We downscaled the resolution to 1 km x 1 km using the grid from section S2.1. Figure I shows the spatial distributions of land cover types in the study area.

The Copernicus global map CLC100 has 23 classes of land cover[8]. We aggregated vegetation containing types into four groups:

(i) bare/sparse vegetation, under the name *sparse vegetation*);
(ii) shrubs, herbaceous vegetation and herbaceous wetland, under the name *shrubs*;
(iii) cultivated and managed vegetation/agriculture, under the name *cropland*; and
(iv) closed forests (5 classes) and open forests (6 classes), under the name *forest*.

[5] https://files.isric.org/soilgrids/former/2017-03-10/data/
[6] https://files.isric.org/public/afsis250m/
[7] https://zenodo.org/record/3939050
[8] https://zenodo.org/record/3939050

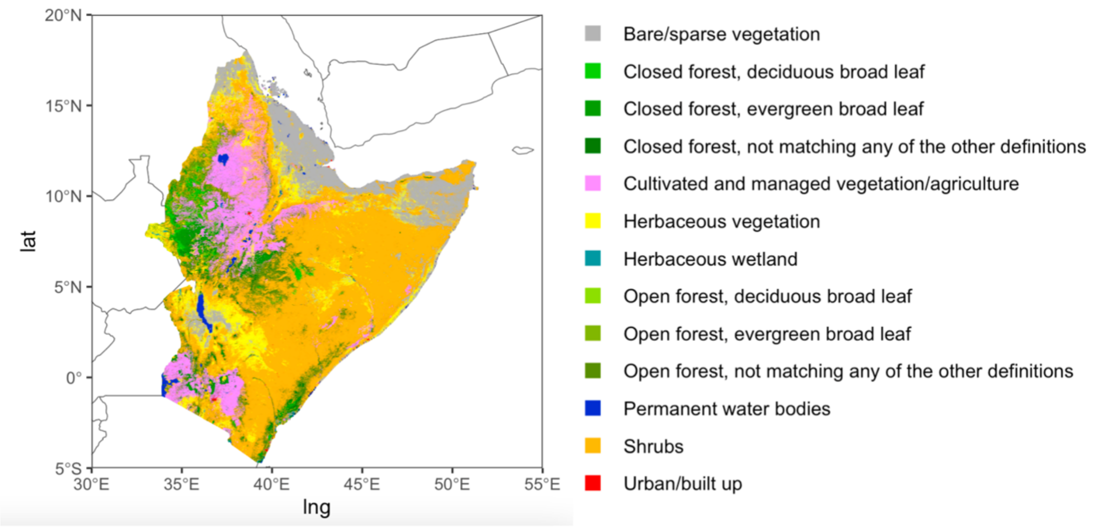

Figure I. *Maps showing distribution of land cover types* (CLC100)[9]. *The base layer of the map is sourced from Natural Earth (https://www.naturalearthdata.com).*

Code for downscaling land cover data is available in file '2_5 Land cover.R'.

S2.6. MODIS NDVI data

For normalized difference vegetation index (NDVI) values, we used 16-day averaged moderate-resolution imaging spectroradiometer (MODIS) data (NASA LP DAAC at the USGS EROS Center Terra Vegetation Indices 16-Day Global MOD13Q1.061)[10]. The data for Kenya, Ethiopia, Somalia, Djibouti and Eritrea and dates in the range between 1st January 2019 and 1st October 2023 were downloaded from Google Earth Engine at 1 km x 1 km resolution. An example of the spatial distribution of NDVI is shown in Figure J.

[9] https://zenodo.org/record/3939050

[10] https://doi.org/10.5067/MODIS/MOD13Q1.061

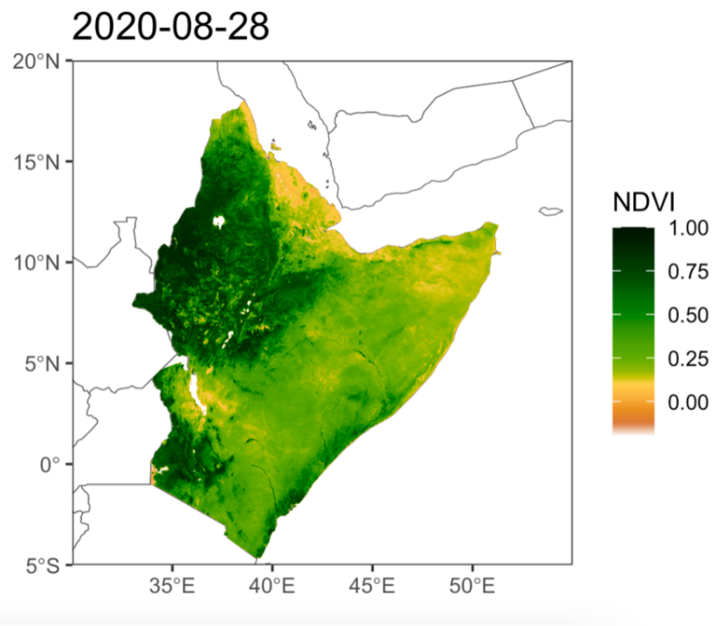

Figure J. *Distribution of NDVI values for 28th of August 2020. The base layer of the map is sourced from Natural Earth (https://www.naturalearthdata.com).*

The Javascript code ‘2_6 MODIS NDVI data.txt’ is available in the repository.

S2.7. The Met Office NAME model: temperature, soil moisture and precipitation

The Met Office's Numerical Atmospheric-dispersion Modelling Environment (NAME) is used to model a wide range of atmospheric dispersion events[11]. The predictions are driven using historic analysis meteorology data from the global configuration of the Unified Model (UM)[12]. The UM provides 0 - 10 cm soil moisture at 3 hourly time intervals on its native horizontal grid of approximately 0.1^0 equivalent to approximately 10 km x 16 km at the equator. An example of spatial resolution of temperature and soil moisture is shown in Figure K. As this is a lower resolution than the resolution we use for the model, we upscaled values by assigning this value to all 1 km x 1 km grid cells within a 10 km by 10 km area.

[11] https://doi.org/10.1007/978-0-387-68854-1_62
[12] https://www.metoffice.gov.uk/research/approach/modelling-systems/unified-model

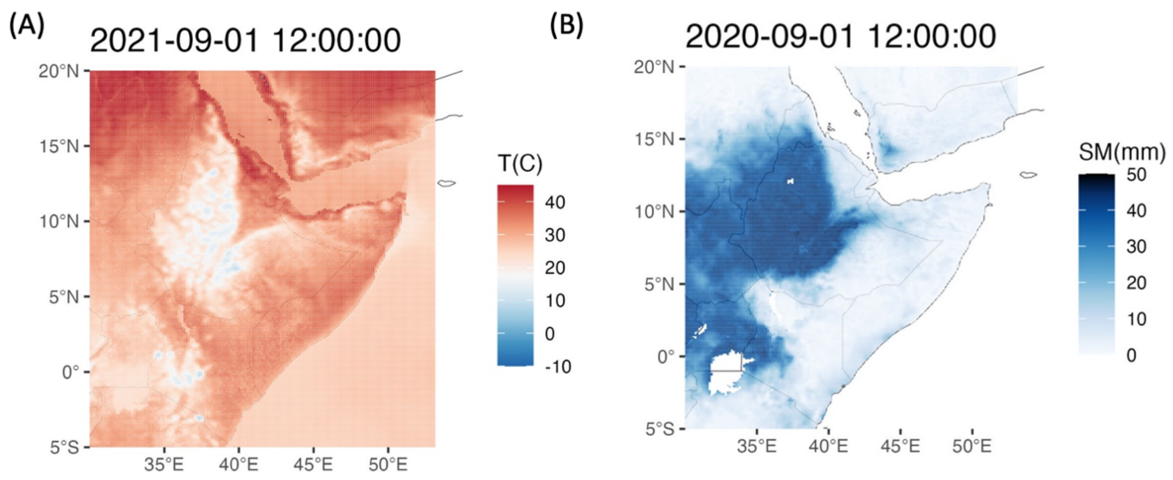

Figure K. *Distribution of temperature (A) and soil moisture(B) at 12.00 (EAT) on 1st Sept 2020. The base layer of the map is sourced from Natural Earth (https://www.naturalearthdata.com).*

S2.8. The Met Office NAME model: wind trajectories

The model (that incorporates a Lagrangian Particle Dispersion Model) can simulate wind trajectories that are stochastic so that an ensemble of trajectories provides a probabilistic distribution of the dispersal of particulates such as spores or insects. NAME wind trajectories were calculated starting from 3860 source locations spaced on a regular 20 km x 20 km grid across Ethiopia, Somalia, Eritrea and Kenya (Fig L(A)). For a given date and source location, 1000 individual trajectories were computed, each commencing 2h after local sunrise and terminating 1h before local sunset. An example of NAME wind trajectories at a particular location is shown in Fig L(B).

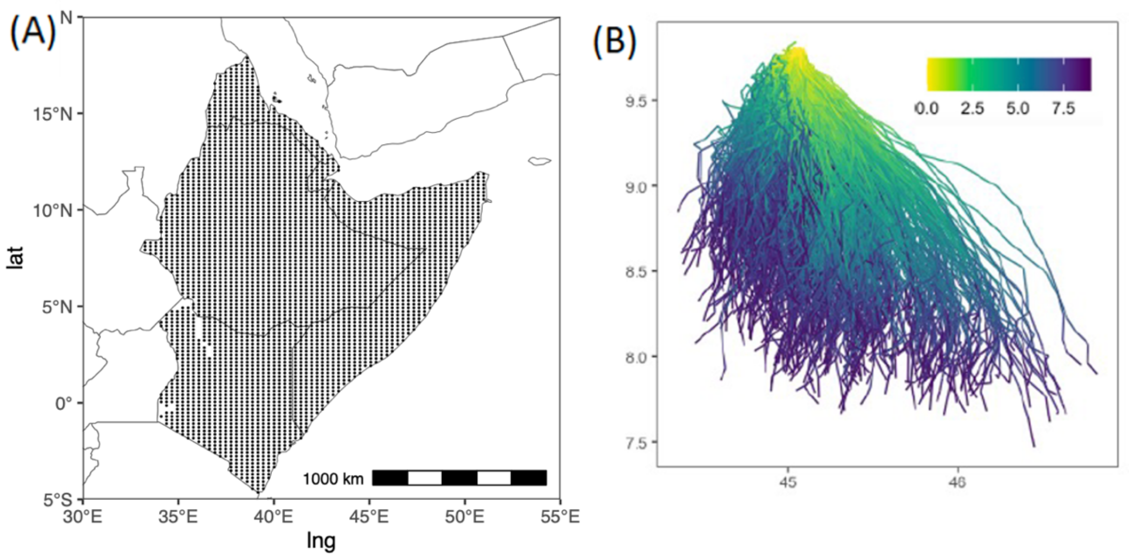

Figure L. *(A) Source locations for wind trajectories. (B) An example of NAME wind trajectories on the 15th of January 2021. The colour shows the difference in hours between points on a trajectory and the initial point. The base layer of the map is sourced from Natural Earth (https://www.naturalearthdata.com(.*

An example of files required to produce NAME trajectories is available in the repository: '2_8_1_maininput.txt', '2_8_1_output.txt', '2_8_1_sourcelocations.txt', '2_8_1_sources.txt' and '2_8_1_tgrids.txt'.

S3. NDVI data analysis

In this section we described pre-processing of NDVI data. As remote sensing data are usually noisy, we smoothed NDVI time series before performing any further analysis. An important property of NDVI is that it is dynamic for many types of vegetation. As an indicator of the change in NDVI value, we calculated NDVI trend during the period of 150 days prior to the date of interest. The time resolution of the NDVI series is 14 days. The NDVI trend was assumed to be in one of three states: increasing NDVI values, constant NDVI values or decreasing NDVI values. Finally, we performed a peak search in the NDVI time series. The presence of peaks indicates that there was an onset of fresh vegetation during the period.

S3.1. Smoothing of NDVI data

For smoothing of NDVI data, we applied a Whittaker smoother[13]. We implemented the algorithm provided by Eilers *et al.* 2017[14]. The algorithm is very fast as it is based on operations with sparse matrices. The amount of removed noise depends on the smoothing parameter, lambda, which we varied between 10^{-1} and 10^{1}. Figure M(A) shows an example of raw NDVI data and smoothed curves for three values of the smoothing parameter.

One way to quantify choice of smoothing parameter is leave-one-out cross-validation[15]. This is done by leaving out each non-missing observation in turn, smoothing the incomplete data and obtaining a fitted value for the left-out observation. Then the cross-validation (CV) error can be calculated as $\sqrt{\sum_i (y_{-i} - \hat{y}_i)^2}$. The CV error as a function of the smoothing parameter for two NDVI profiles is shown in Figure M(B). We can determine which of the smoothing values produce the smallest error, in this case these are 1.5 and 1. We chose the value of the smoothing parameter to be equal to one.

---

13 https://doi.org/10.1177/1471082x14549288
14 https://doi.org/10.1109/multi-temp.2017.8076705
15 https://doi.org/10.1109/multi-temp.2017.8076705

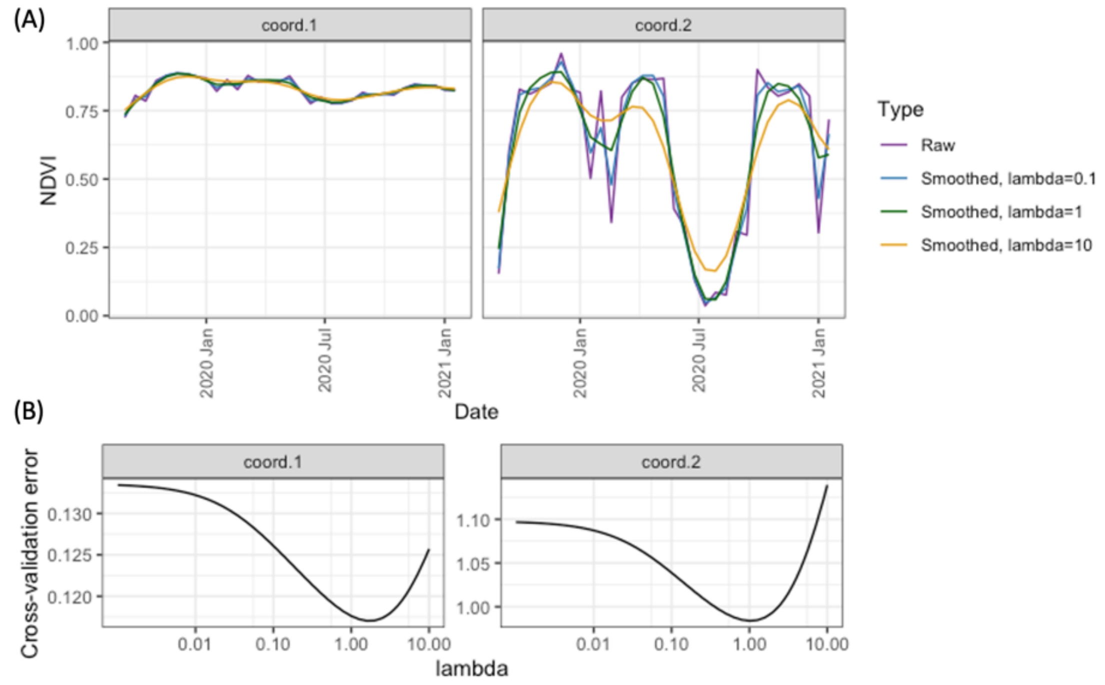

Figure M. *(A) Exemplar NDVI-profiles, and (B) cross-validation error as a function of the smoothing parameter. Coordinates of two location are Coord 1(34.10156, 0.046875) and Coord 2(36.63281, 7.171875).*

S3.2. Assessing NDVI trend

The NDVI trend was estimated in two steps. First, we used the R package **segmented**[16] to calculate breakpoints of the smoothed NDVI curve. Then we combined segments that had the same direction of change, i.e. increasing NDVI value or decreasing NDVI value. If the range of NDVI values within a segment was 0.1 or less, we assigned the trend as constant. The last segment before the swarm landing date was then used to define the NDVI trend. Figure N shows examples of trends for three lengths of window for the NDVI analysis (60 days, 150 days and 300 days prior to the date of interest). Red lines show the start and end of the trend period for increasing and decreasing NDVI, and blue lines show the range of the NDVI values during this period. The classification of trend was not sensitive to the choice of the analysis window. However, the 60 days window was too short to correctly estimate the start and end period for the NDVI trend and the range of the NDVI. Estimation based on 150 days and 300 days prior to the date of interest gave the same results but producing NDVI profile for 300 days requires accessing 2x more datasets. We therefore chose the analysis window of 150 days prior to the date of interest from which to estimate the NDVI trend.

[16] https://doi.org/10.1111/anzs.12200

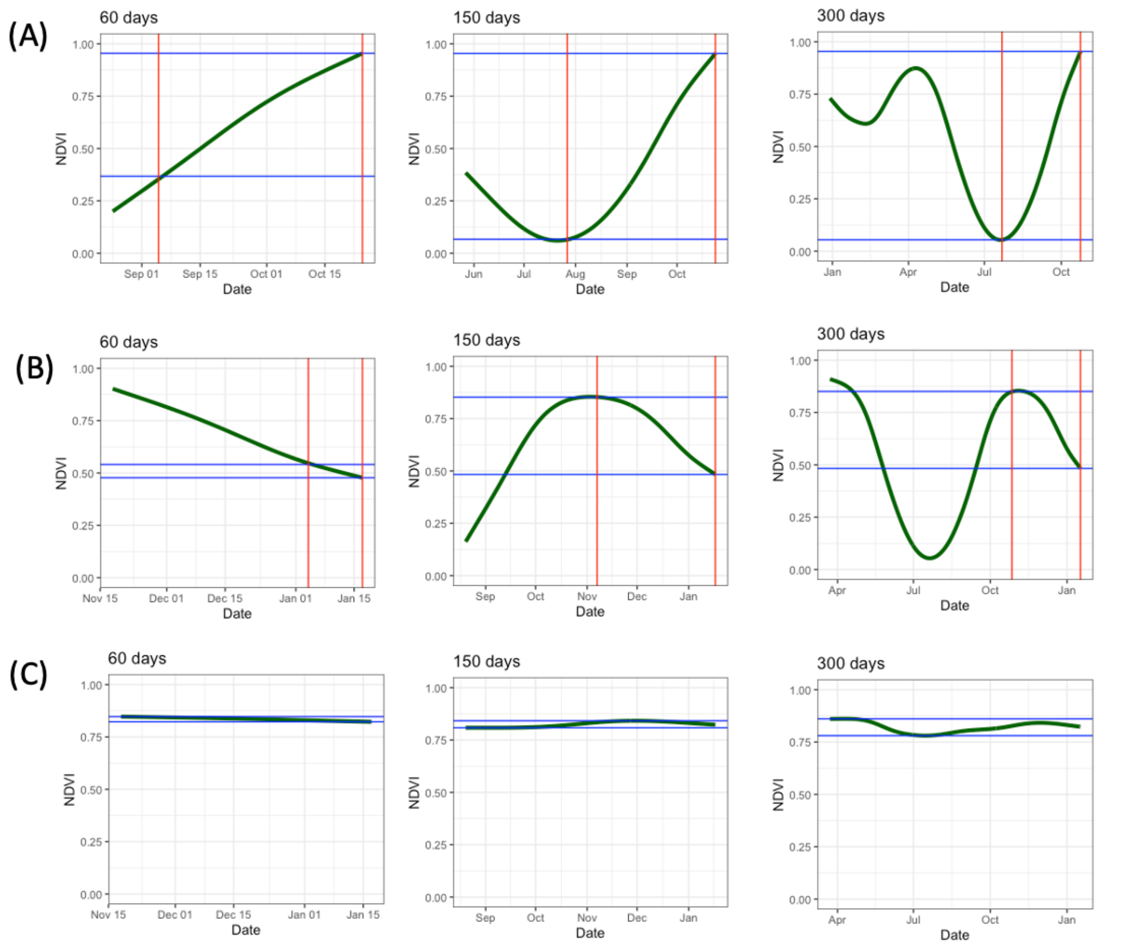

Figure N. *Examples of estimated NDVI trends: (A) increasing; (B) decreasing; (C) constant. Here red vertical lines show the start and end of time period for the NDVI trend.*

S3.3. Finding peaks in a NDVI time serries

We used the procedure 'findPeaks' from the R repository 'stas-G'[17]. A peak is defined as a point such that *m* points either side of it have a lower or equal value to the point. Figure O shows examples of peak finding for *m = 2*, *m = 10* and *m = 50.* We concluded that two points either side was enough to identify all local peaks, so we set *m = 2*.

[17] https://github.com/stas-g/findPeaks

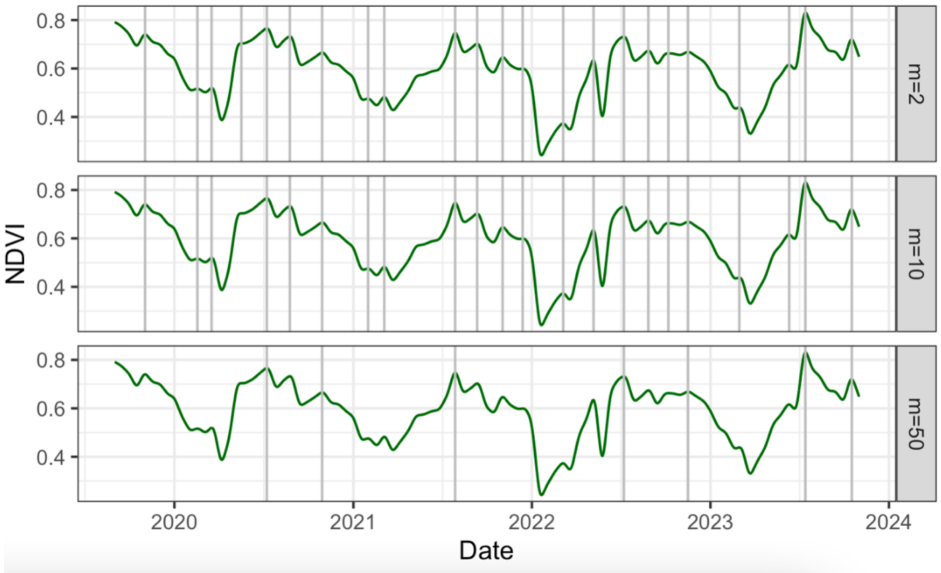

Figure O. *Examples of peak finding for different values of smoothing parameter (m).*

S4. Finding areas suitable for breeding

We used machine learning to predict breeding suitability. The model was trained and validated using hoppers/bands locust presence and absence data. We used elevation, sand content in soil and clay content in soil as covariates. In the absence of field data on breeding sites in the Horn of Africa, we compared our predicted breeding suitability for Kenya with the predicted breeding probabilities by Kimathi *et al.* 2020[18] in order to assess the broad consistency of the approaches.

S4.1. Desert locust data and environmental covariates

Data on presence and absence are discussed in Section 2.2.2. We used 80% of the data for model training and 20% of the data for model validation. We restricted our analysis to using three static variables: elevation; sand content in the soil at 5 cm depth; and clay content in the soil at 5 cm depth.

S4.2. Machine learning approach to map potential breeding sites

Breeding suitability mapping calculations were performed on the Google Earth Engine platform[19]. We used a Random Forest classifier[20]. The importance of covariates was obtained by normalising the classifier importance values. The breeding suitability values were adjusted to a 0-100 scale.

[18] https://doi.org/10.1038/s41598-020-68895-2
[19] https://doi.org/10.1016/j.rse.2017.06.031
[20] https://doi.org/10.1007/978-3-642-31537-4_13

S4.3. Running the breeding suitability prediction code

The code to predict breeding suitability across a landscape domain can be run in a browser at Google Earth Engine (GEE) https://code.earthengine.google.com. The code requires training data (as a CSV file), sand content data (as a tiff file) and clay content data (as a tiff file) to be added as assets to a Google Earth Engine (GEE) account. The elevation data are ingested in GEE (dataset "CGIAR/SRTM90_V4") and can be loaded directly.

The Javascript code '2_4 Running breeding suitability prediction code.txt ' is available in the repository.

S4.4. Choosing the number of decision trees

We varied the number of trees from 2 to 500. We used the GEE command '.explain()' to obtain information about the trained classifier, which among others gives values of an 'Out-of-Bag' error estimate corresponding to a given number of trees. Our analyses showed that the 'Out-of-Bag' error associated with prediction did not decrease significantly when the number of decision trees was set higher than 100 (Figure P).

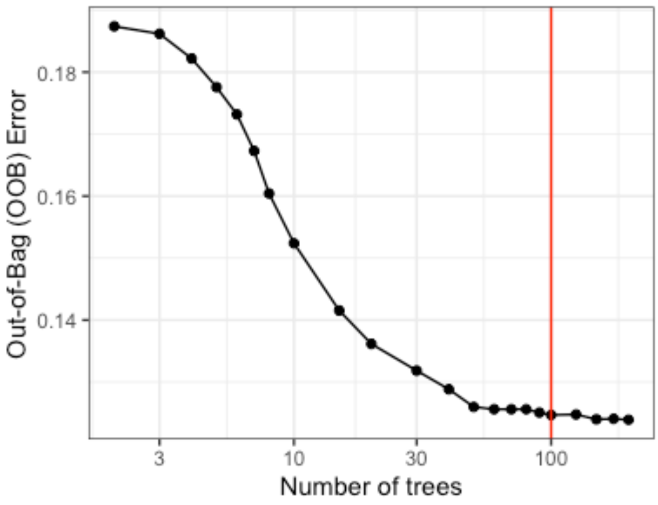

Figure P. *'Out-of-Bag' error as a function of number of trees. The red vertical line shows our chosen number of trees.*

S4.5. Predicted breeding suitability across the domain.

The classifier was run with the following options: the number of decision trees was set to 100, the number of variables per split was set to 2, the 17 minimum leaf population (i.e. the end node) was set to 2, and the initial seed was set to 0. The estimated breeding suitability map is shown in Figure Q.

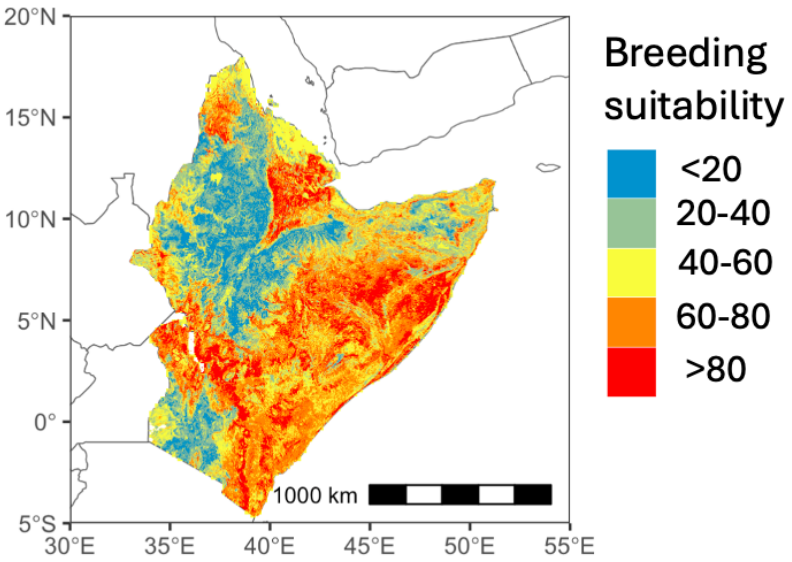

Figure Q. *Breeding suitability map for desert locust. The breeding suitability values were adjusted to a 0-100 scale. The base layer of the map is sourced from Natural Earth (https://www.naturalearthdata.com).*

S4.6. Evaluating performance of the classifier

We used the **R** package **PresenceAbsence**[21] to evaluate the results of the presence-absence analysis. For the validation data set, we obtained the following accuracy statistics values: percent correctly classified = 0.83, sensitivity = 0.9, specificity = 0.71, and area under the curve = 0.91. Figure R shows the area under the curve of the Receiver Operating Characteristic (ROC) curve.

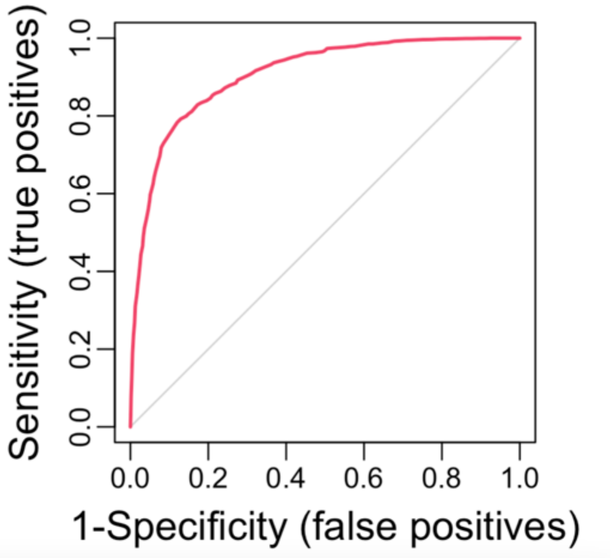

[21] https://www.jstatsoft.org/article/view/v023i11

Figure R. *The area under the curve of the Receiver Operating Characteristic (ROC) curve to evaluate the results of the presence-absence analysis for breeding suitability for desert locust.*

Figure R can be reproduced using file ‘4_6 Evaluating performance of the classifier.R’.

S4.7. Comparison with published maps

Kimathi *et al.* 2020 used datasets at a spatial resolution of 1 km, with the exception of soil moisture, for which the resolution was downscaled from approximately 55 km to 1 km. Figure S(A) from Kimathi *et al.* 2020 was converted into geotiff and values extracted using the R package **raster**. We found broad correspondence at high and very high suitability breeding regions between the two models (Figure S) albeit the higher resolution of the model introduced here indicates greater spatial heterogeneity in the suitability scores.

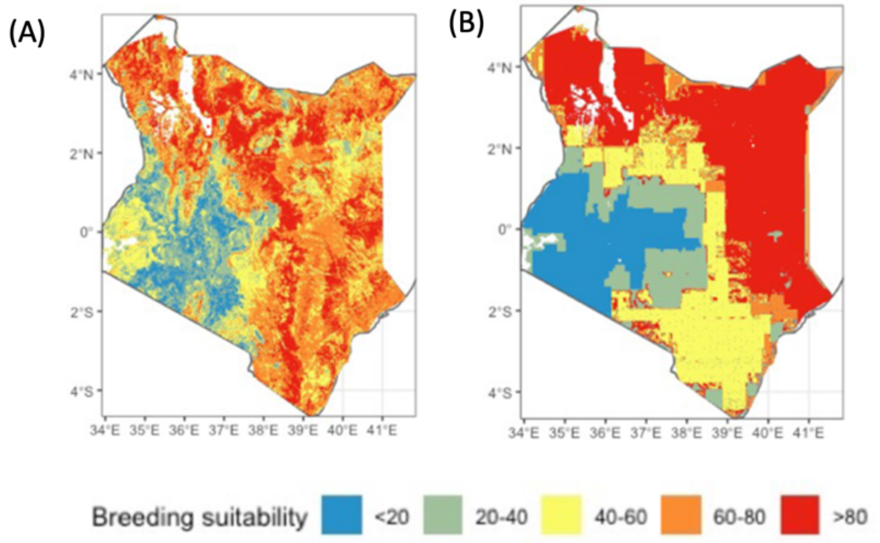

Figure S. *Comparing breeding suitability for desert locust: (A) this study; (B)* Kimathi *et al.* 2020[22]*. The base layer of the map is sourced from Natural Earth (https://www.naturalearthdata.com).*

S5. Egg laying

We separated the breeding process into two components: (i) testing areas suitable for DL breeding, and (ii) testing if there was either a precipitation episode or there was enough soil moisture (12 mm or above) to enable successful development of eggs.

[22] https://doi.org/10.1038/s41598-020-68895-2

S5.1. Testing if area is suitable for egg laying.

First, an area where breeding and egg laying was expected to occur was chosen. We consulted Desert Locust briefs[23] where swarms had been reported. Next, locations were sampled randomly from the defined area. For each sampled location we determined if the location was suitable for breeding by drawing a number from a uniform random distribution and testing if the number was smaller than the value from the breeding suitability map shown in Figure S. If the step was successful, i.e. breeding was possible, we proceeded to the next test.

S5.2. Testing if conditions are suitable for egg laying.

We required either a precipitation event (precipitation> 0) or soil moisture to be above 12 mm a day prior to egg laying. To test if the condition was satisfied, we used precipitation and soil moisture predictions from the Met Office's operational Numerical Weather Prediction model, NAME[24].

[23] https://www.fao.org/ag/locusts/en/archives/briefs/2515/2516/index.html
[24] https://gmd.copernicus.org/articles/12/1909/2019/

S6. Development from eggs to hoppers

We used an analytical dependence between temperature and percentage daily development to calculated the length of the development period from eggs to hoppers. We used the UK Met Office NAME environment to provide temperature values at 3 hourly time intervals. The length of the period required for eggs to develop depended on a particular location and time. If the calculated egg development period was outside 10-50 days[11], we assumed that egg development was unsuccessful.

S6.1. Time required for eggs to develop.

The percentage daily development of eggs can be expressed as a function of temperature (in Celsius)[25]:

$$q_{egg}(T) = \begin{cases} 9.41\, exp(-0.00357(35.019 - T)^2), & if\ 10 \le T \le 34, \\ 0, & otherwise. \end{cases}$$

To calculate the time required for eggs to develop, we integrated the above equation until the first day it reached 100%. An example is shown in Figure T.

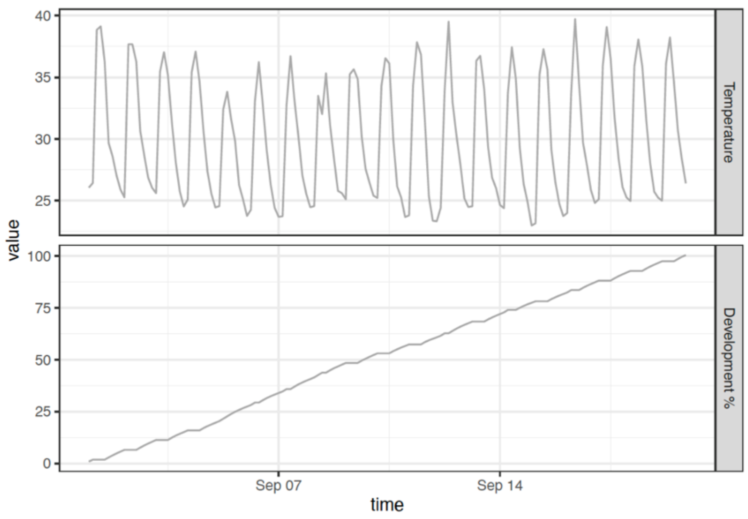

Figure T. *An example of temperature and corresponding accumulation of development rate of desert locust eggs.*

S6.2. An example of egg development period distribution.

The spatial distribution of duration of egg development for eggs deposited in the study area on 1st of September 2020 is shown in Figure U. The average egg development period was 19.1 days (Figure U(B)).

[25] Pedgley, D. 1981. Desert Locust Forecasting Manual. London: Centre for Overseas Pest Research

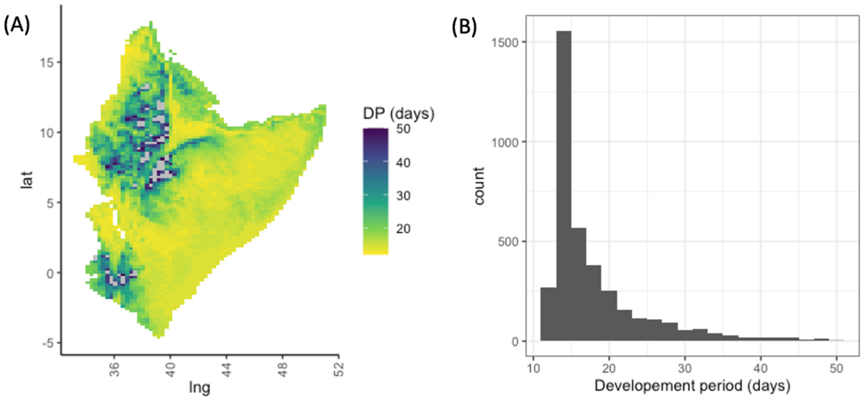

Figure U. *Spatial distribution (A) and histogram (B) of egg development periods for desert locust eggs deposited on 1st September 2020. Gray shows areas which are not suitable for development. The base layer of the map is sourced from Natural Earth (https://www.naturalearthdata.com).*

Figure U can be reproduced using file '6_2 Egg development period.R'.

S7. Development from hoppers to adults

Similar to calculations for the egg development period, we used analytical dependence between temperature and percentage daily development to calculate the length of the development period from hoppers to adults. We used the UK Met Office NAME environment to provide temperature values at 3 hourly time intervals. The length of the period required for eggs to develop depended on a particular location and time. If the calculated hopper development period was outside 24-95 days[11], we assumed that hopper development was unsuccessful. We plotted hopper development distributions for three chosen dates. An additional test for successful hopper development checked if environmental conditions were suitable for hopper feeding.

S7.1. Time required for hoppers to develop into adults.

The percentage daily development of hoppers can be expressed as a function of temperature (in Celsius)[26]:

$$q_{hopp}(T) = \begin{cases} 0.222T - 3.166, & if\ 23 \le T \le 32, \\ 0, & otherwise. \end{cases}$$

We integrated the above equation until the first day it reached 100. This gives the length of period required for eggs to develop for a particular location and time. An example is shown in Figure V.

[26] https://doi.org/10.1017/s0007485300031321

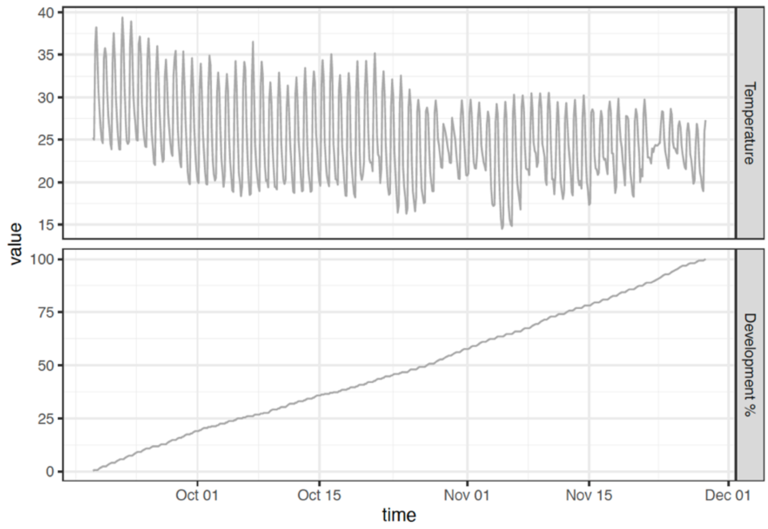

Figure V. *An example of temperature and corresponding accumulation of rate development rate of desert locust hoppers.*

S7.2. Analysis of hopper development period.

The spatial distribution of the duration of hopper development for hoppers that hatch in the study area on 1st October 2020 is shown in Figure W(A). The average hopper development period was 50.9 days (Figure W(B)).

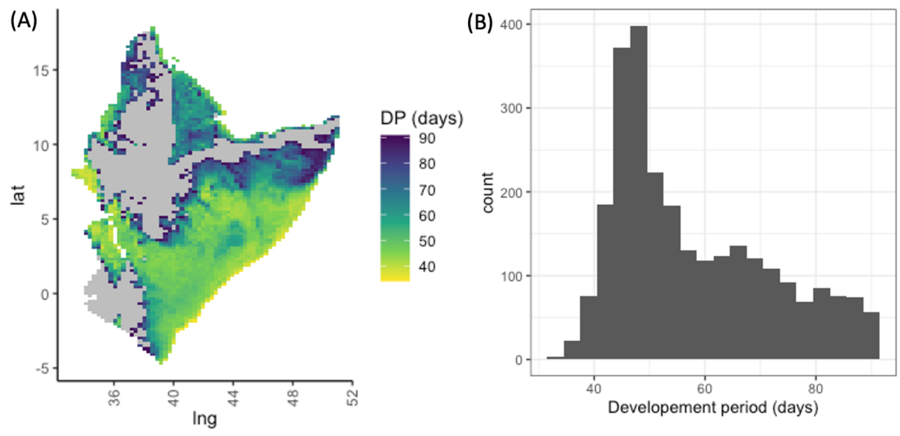

Figure W. *Spatial distribution (A) and histogram (B) of desert locust hopper development periods for hoppers hatching on 1st October 2020. Gray shows areas which are not suitable for development. The base layer of the map is sourced from Natural Earth (https://www.naturalearthdata.com).*

Figure W can be reproduced using file '7_2 Hopper development period.R'.

S7.3. Conditions required for hoppers to develop.

First, if the calculated hopper development period was outside 24-95 days[11], we assumed that hopper development was unsuccessful. When the calculated development period lay within the range 24-95 days, we tested if the environmental conditions were suitable for hopper feeding with success determined if either of two conditions were satisfied during the hopper development period: (i) smoothed NDVI had at least one peak; or (ii) smoothed NDVI was above a threshold, which we set to be 0.09[27].

S8. Estimating food availability for migrating swarms

The aim of analysis was to evaluate how vegetation at landing sites could satisfy feeding requirements of a swarm with the objective of translating the state of vegetation at landing sites into potential feeding periods for a swarm. We reviewed at the literature for information on food requirement for an average swarm and the relationship between land cover type, NDVI, and above ground biomass. There is no universal method to convert NDVI values into above-ground biomass; this requires field data or estimated biomass from the region of interest and for a range of typical NDVI values corresponding to different vegetation communities[28]. Usually, data are collected at lower scales (in the order of meters) and translated to higher scales (hectares or kilometres), which can introduce high levels of uncertainty. We investigated three factors that are important when determining food availability: land cover type, current value of NDVI (vegetation density), and NDVI trend (increasing, decreasing or constant). Based on the values of these factors, we derived rules for estimating feeding period.

S8.1. Food requirement for an average swarm

The daily feeding-rates of individual desert locust vary with age and activity: adults eat roughly half their own weight (~1.0g), while a migrating adult swarming locust would need to eat its own weight and possibly three times as much (2.0g-5.0g)[29]. There is wide variability in estimates of the density and size of locust swarms: from 30 locusts per square yard[30] to 10^9 locusts in a swarm covering an area of 20 square kilometres[31]. This gives a range of locust density between 3.5 x 10^7 to 5 10^7 locusts per 1 km^2. If we assume that a locust eats its own weigh, then the required amount of food consumed by a swarm per square kilometre would be between 70 and 100 metric tonnes of plant material per day.

S8.2. Relationship between land cover type, NDVI, and aboveground biomass

Analysis based on mixed gardens showed that NDVI values between 0.73 and 0.85 corresponded to the total carbon stock between 0.1 and 1.8 tonnes per 30m x 30m pixel[32]. The estimation of carbon content was obtained by multiplying the result of aboveground

[27] https://doi.org/10.1111/j.0906-7590.2004.03779.x
[28] https://doi.org/10.3390/en17010231
[29] https://doi.org/10.2307/2401671
[30] https://doi.org/10.2307/2401671
[31] https://www.cabidigitallibrary.org/doi/full/10.5555/19640501858
[32] https://doi.org/10.1088/1755-1315/1211/1/012015

biomass measurements with 47% conversion factors[33]. This allowed us to convert carbon content back to aboveground mass. For a mangrove forest, NDVI values between 0.15 and 0.45 correspond to aboveground biomass between 1 and 26 Kg per 5 m x 5 m pixel[34]. The relationship between NDVI and aboveground biomass has been investigated for only a few types of crops: NDVI values ranging from 0.1 to 0.71 correspond to aboveground biomass between 4 to 56 grams per square meter for wheat[35], and NDVI values ranging from 0.4 to 0.9 correspond to aboveground biomass between 500 to 5000 kg per ha for soybean[36]. For shrubs, NDVI values ranging from 0.05 to 0.7 correspond to aboveground biomass between 0 to 40 Mg per ha[37], and NDVI values ranging from 0.3 to 0.6 correspond to aboveground biomass between 400 to 700 g per m^2[38]. Figure X shows the data scaled to tonnes per km^2. It can be seen there is a lot of variability when evaluating biomass based on NDVI values. If we assume that the daily plant requirements for a swarm are 100 tonnes per km^2, we expect that there should be enough food for 2-7 days.

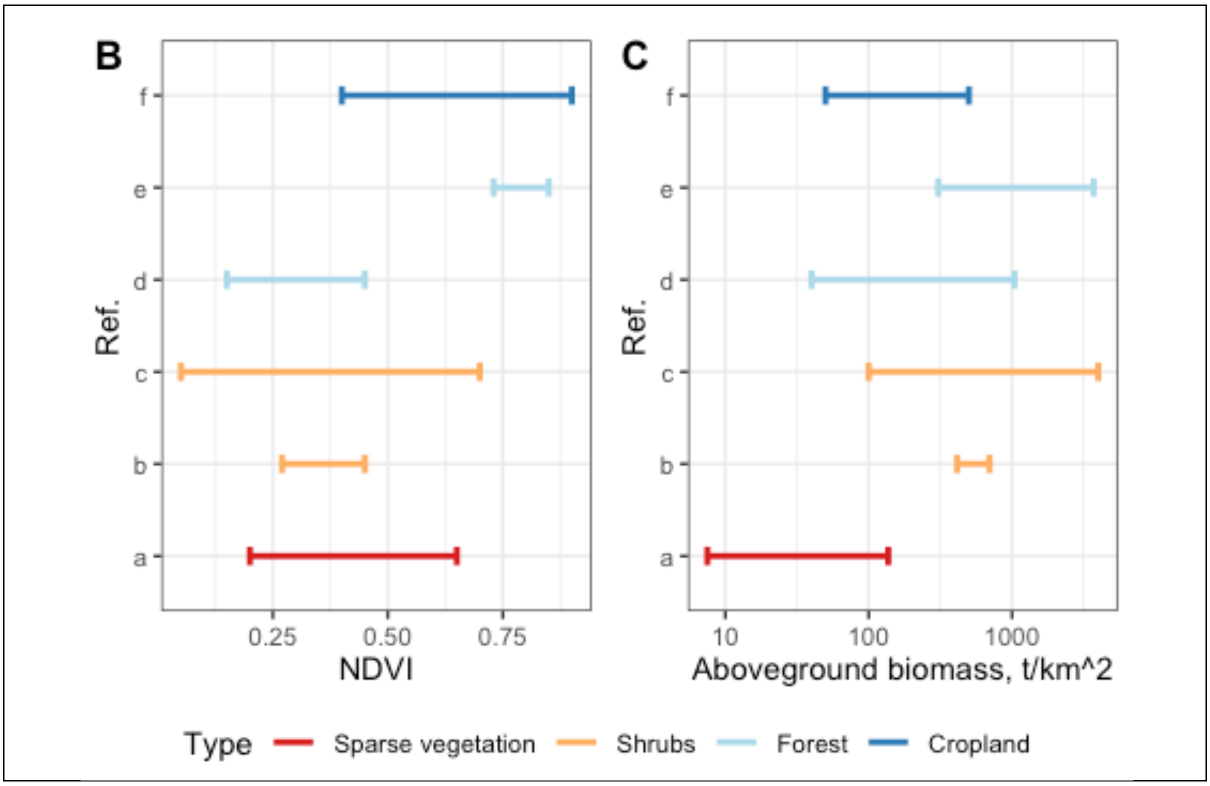

[33] https://library.itc.utwente.nl/papers_2019/msc/nrm/kustiyanto.pdf
[34] https://doi.org/10.1016/j.ejrs.2018.04.006
[35] https://doi.org/10.1556/CRC.39.2011.1.15
[36] https://doi.org/10.3389/fsufs.2022.959681
[37] https://doi.org/10.3390/f11050555
[38] https://doi.org/10.1016/j.rse.2004.01.010

Figure X. *Ranges of estimated NDVI (A) and aboveground biomass (B): sparse vegetation (a[39]), shrubs (b[40], c[41]), forest (d[42], e[43]) and cropland (f[44]).*

S8.3. Relationship between land cover types and NDVI density

An additional criterion for classification is given by the density of vegetation, which we divided into five classes according to the minimum value of the NDVI[45]: lowest density (NDVI ∈ (0, 0.15)), lower density (NDVI ∈ [0.15, 0.3)), dense vegetation (NDVI ∈ [0.3, 0.45)), higher density (NDVI ∈ [0.45, 0.6), highest density (NDVI ∈ [0.6, 1]). Figure Y shows examples of smoothed profiles between September 2019 and January 2021 and their classification according to type of land cover and vegetation density.

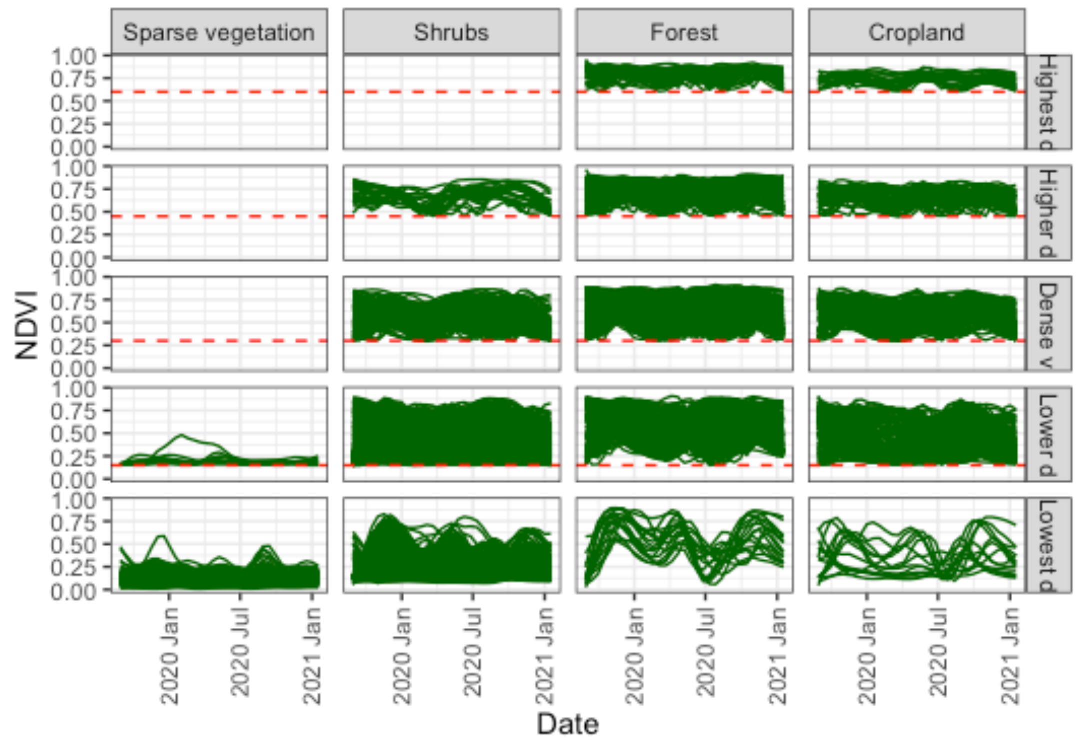

Figure Y. *Smoothed NDVI profile was classified into a particular class if the minimum was above the required threshold of the class (red dashed line).*

S8.4. Feeding period

Based on the above analysis, we divided feeding periods into three classes: short stay (1-2 days), medium stay (2-4 days), and long stay (4-7 days). Figure Z shows how we assigned the class of stay for swarms depending on the environmental conditions at a given location. The

[39] http://dx.doi.org/10.2480/agrmet.69.1.1
[40] http://dx.doi.org/10.3390/f11050555
[41] http://dx.doi.org/10.1016/j.rse.2004.01.010
[42] http://dx.doi.org/10.1088/1755-1315/1211/1/012015}
[43] http://dx.doi.org/10.1016/j.ejrs.2018.04.006
[44] http://dx.doi.org/10.3389/fsufs.2022.959681
[45] https://doi.org/10.1088/1755-1315/126/1/012112

number of days a swarm stayed and fed was sampled from the range corresponding to the class to which the location was assigned. For NDVI values less than zero, swarms would stay only overnight and start flying in the morning.

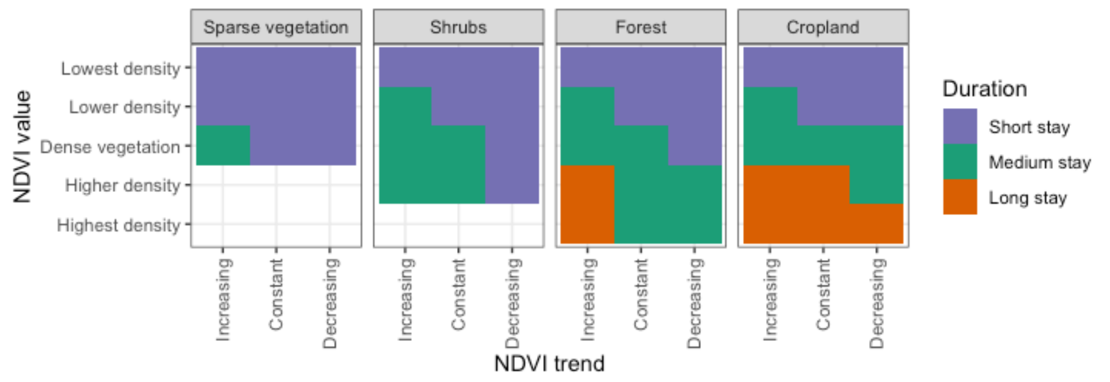

Figure Z. *Length of stay for swarms depends on land cover type, NDVI trend (increasing, decreasing or constant) and vegetation state (value of NDVI). Here short stay is 1-2 days, medium stay is 2-4 days, and long stay is 4-7 days.*

S8.5. Spatial distribution of feeding period

An example of the spatial distribution of potential length of stay on 15th of January 2021 is shown in Figure AA.

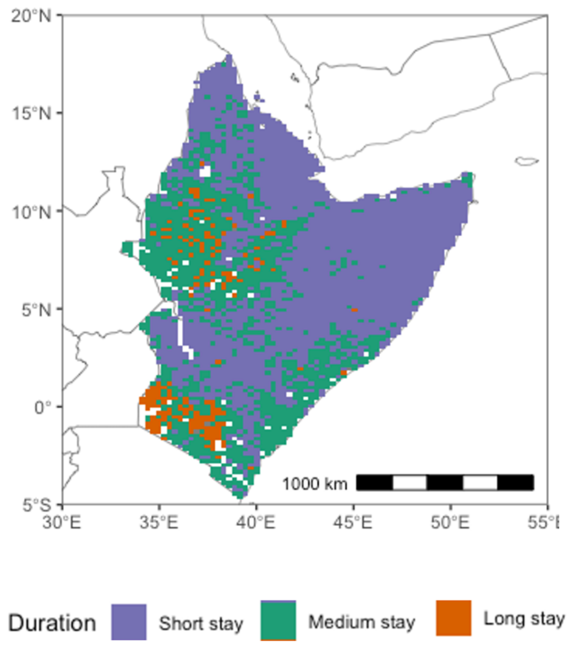

Figure AA. *Spatial distribution of stay length of desert locust swarms on* 15th *of January 2021. Length of stay was classified as short stay (1-2 days), medium stay (2-4 days), or long stay (4-7 days). The base layer of the map is sourced from Natural Earth (https://www.naturalearthdata.com).*

Figure AA can be reproduced using file '8_4 Spatial distribution of feeding period.R'.

S9. Estimating time periods suitable for migration

For this analysis, we first calculated the direction of wind trajectories. We chose three locations that could act as stepping stones for swarm migration from breeding sites in Somalia to Kenya. We fitted the von Mises distribution to daily wind trajectories at these three locations. Temporal changes in the directional mean showed seasonal variation in global wind patterns, while the concentration served as a measure of stochasticity associated with natural variation in turbulent wind flow[46]. Analysing the distribution of the directional mean allowed us to identify time periods when wind direction was suitable for swarms to reach Kenya from breeding sites in Somalia.

S9.1. Calculating wind direction angle

We calculated the angle for each trajectory between a vector corresponding to the East direction and a vector connecting the start and end location of a trajectory for a specified time (Figure BB(A)). To obtain the distribution of wind direction on a particular day and location, we calculated angles for all 1000 wind trajectories (Figure BB(B)).

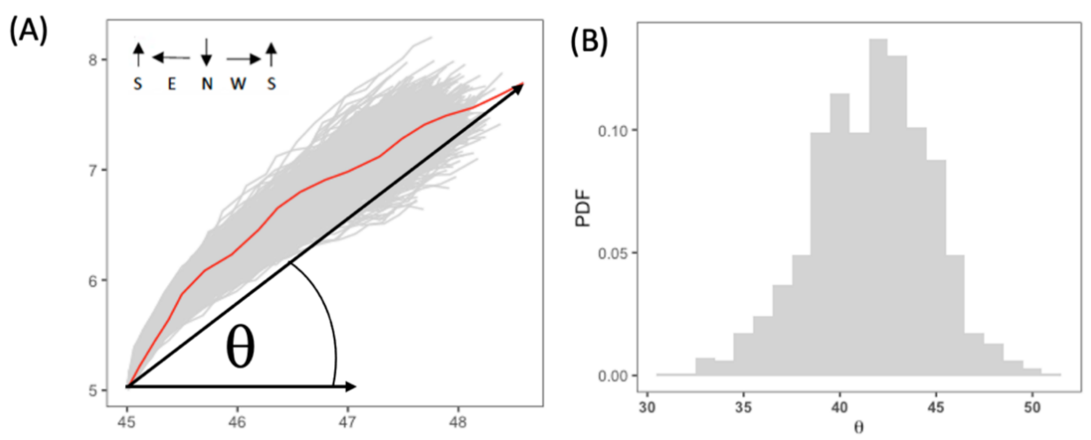

Figure BB. *(A) Calculations of wind direction. (B) Corresponding distribution of wind directions.*

S9.2. Temporal distribution of wind direction

For analysis we chose three locations (Figure CC(A)). We calculated daily distribution of wind direction angles for years 2020 and 2021 (Figure CC(B)). The distributions clearly indicated seasonal changes of wind direction in all three locations as well as difference in ranges of wind direction.

[46] https://www.mdpi.com/2571-905X/7/2/26

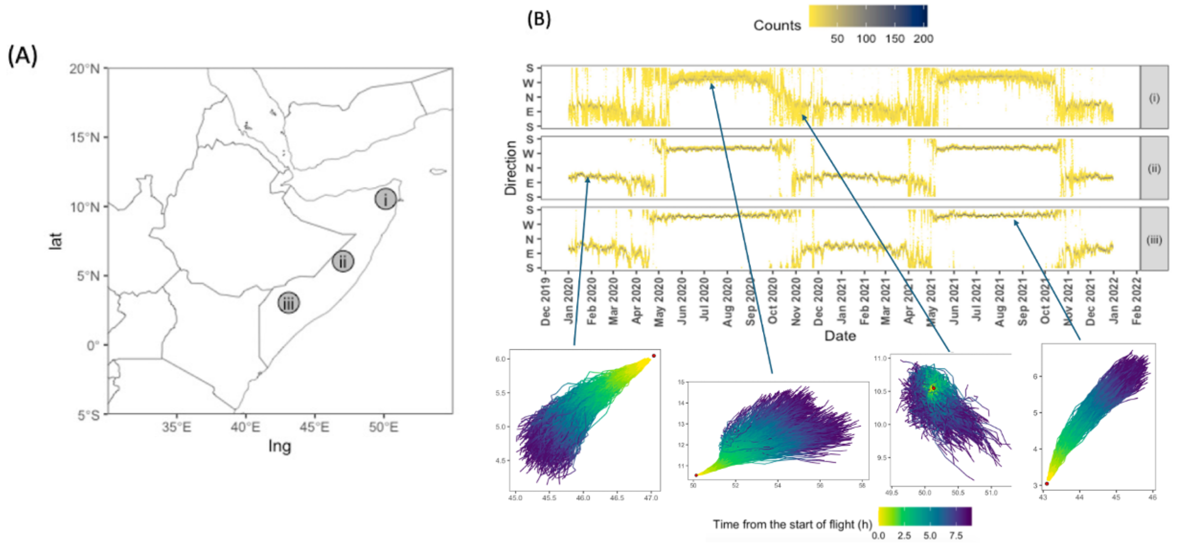

Figure CC. *(A) Three locations for which wind trajectories were analysed. (B) Distribution of trajectory angles during 2020-2021. The lower panel shows few examples of wind trajectory distributions. The base layer of the map is sourced from Natural Earth (https://www.naturalearthdata.com).*

S9.3. Fitting the von Mises distribution

We used Bayesian inference[35] to fit a von Mises distribution to trajectories from the three locations. Fitting was performed for daily trajectories from January 2020 till December 2021. The posterior values are shown in Figure DD. Northerly winds were present in December and January in both 2020 and 2021 and all three locations.

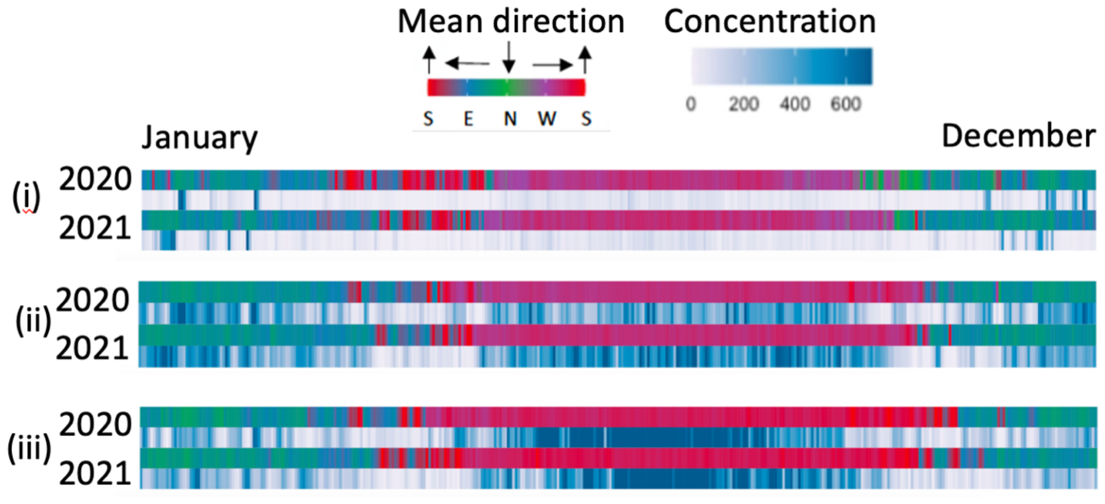

Figure DD. *Fitted parameters of von Mises distribution to direction of wind trajectories: mean direction (top row), and concentration (bottom row).*

Figure DD can be reproduced using file ‘9_3 Characteristics of wind trajectories.R’.

S10. Predicting day to day movement of swarms

Here we consider the application of the framework for modelling of short-term predictive day to day movement of swarms. Short term movement allows for wind-assisted dispersal as swarms disperse to new food sources.

S10.1. Simulating day to day movement of swarms.

When simulating dispersal of a swarm, we sampled a single NAME wind trajectory (Figure EE(A)). We assumed that the propensity of a swarm to start flying in the upcoming days was dependent on food availability on the ground. The number of days before onward movement of a swarm (food period) was sampled uniformly from the appropriate ranges, i.e. short stay (1-2 days), medium stay (2-4 days), or long stay (4-7 days). On the morning of the day the swarm flies, we searched for the closest grid coordinates (Figure EE(B)), randomly sampled a trajectory from the grid point (Figure EE(C)) and then translated the corresponding wind trajectory to start at the required location (Figure EE(D)).

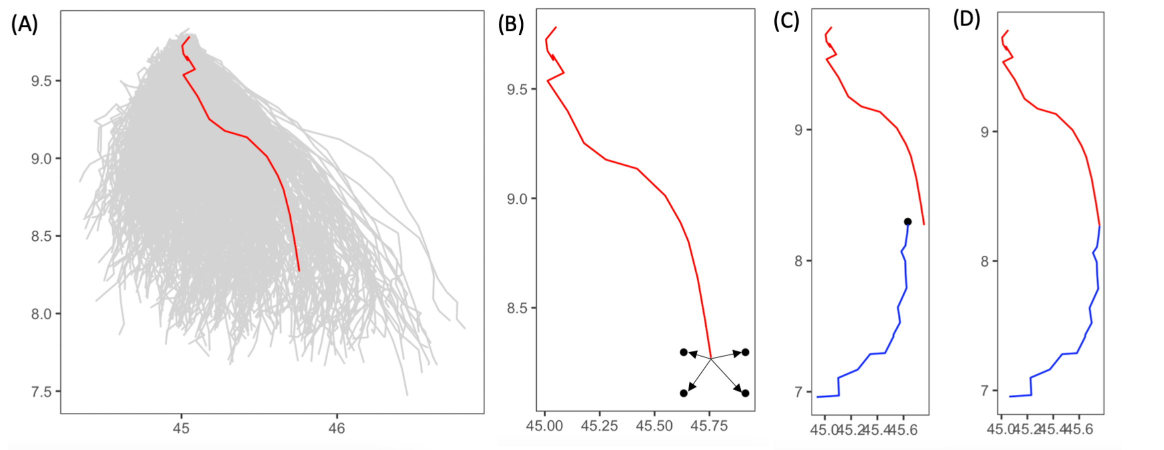

Figure EE. *Simulating day-to day migration of a desert locust swarm. (A) Sample a single wind trajectory from the ensemble. (B) Choose closest source location to the landing site. (C) From this source choose a single trajectory. (D) Translated the corresponding wind trajectory to start at the required location.*

S10.2. Short term prediction of swarm migration

Here we propose how the model could be used for short-term (1-2 days) forecasting of swarm migration. We assumed that swarms started flying in the morning from a particular location (light blue dot in Figure FF(A)) and that a swarm was reported while flying between landing sites (red dot in Figure FF(A)). Consequently, the available data include only the coordinates and date of the sighting, and there is no information about the flight direction, nor is there any knowledge of the location the swarm's flight originated from. The location of the start

and end of the flight for the reported swarm can be estimated by sampling trajectories around the reporting site and retaining those trajectories that pass close to the reporting site. To determine what radius to set for these trajectories, we sampled 100,000 trajectories within 500 km radius and retained those that were within 5 km from the reporting site. The fraction of trajectories passing within 5 km from the reported location as a function of the radius is shown in Figure FF(B). The fraction of accepted trajectories reaches an asymptote at around 250 km.

Short term prediction of swarm migration was simulated as follows. First, we sampled 10,000 flight start coordinates within a 250 km radius around the reporting site (Figure FF(C)). We sampled a trajectory from each starting coordinate for the day of reporting. We retained only those trajectories that passed within 5 km or closer to the reporting location. This provided a short-term forecast of potential landing sites (Figure FF(D)). The landing points of these trajectories were used to derive a risk map of swarm locations at the end of the reporting day. The model can be used to simulate locust migration and feeding for up to seven days in order to mimic a seven-day weather forecast analogous to those used to forecast wheat stem rust[47].

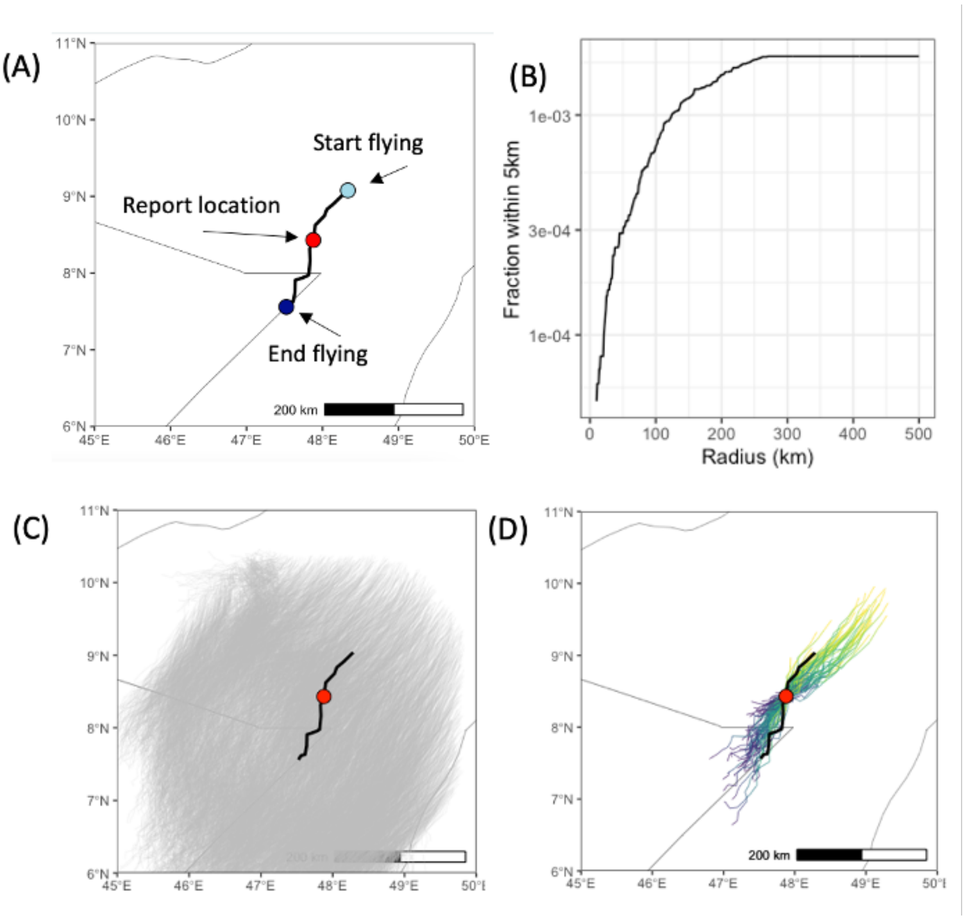

Figure FF. *Short term prediction of desert locust swarm migration. (A) Simulated swarm migration trajectory (black line) and reported location (red dot). (B) Fraction of trajectories passing within 5 km from the reported location as a function of radius for the radius. (C)Trajectories with starting point within 250 km radius. (D) Possible migration trajectories*

[47] https://iopscience.iop.org/article/10.1088/1748-9326/ab4034

on reporting day passing close to the reporting site. The base layer of the map is sourced from Natural Earth (https://www.naturalearthdata.com).

Figure FF can be reproduced using file ‘10_2 Short term prediction of swarm migration.R’.

S11. Simulating breeding, development and movement of DLs

Long-term movement occurs from breeding grounds encompassing egg to hopper and adult development as well as successive movement of swarms conditioned on wind conditions for dispersal and availability of food at landing sites. We illustrate the use of the framework first for longer-term movement of a single swarm emanating from a specific breeding area in Somalia then for multiple swarm movements from two progressively larger potential breeding areas in northern Somalia.

S11.1. Model simulation from a specific breeding site

Simulation started with choosing a location and date for breeding. The location for a potential breeding site in Somalia is shown in Figure GG(A). The date for egg laying was chosen as 1st September 2020. We checked if the location was suitable for egg laying. The breeding sub-model returned a vector with: (i) probability that a site was suitable for breeding; (ii) the maximum amount of precipitation 24-48 hours before; (iii) the maximum level of soil moisture 24-48 hours before. For this particular location, the probability of being suitable for breeding was 0.65. The precipitation profile showed that there was an episode of rain the day before (Figure GG(B)). Based on temperature values, we accumulated daily development rate to obtain a day when hoppers would have hatched, which was 19th September 2020 (Figure GG(C)). Again, based on temperature values, we accumulated daily development rate to obtain a date when hoppers turned into adults. This happened on 28th November 2020 (Figure GG(D)). Next, we tested if there was enough vegetation on the ground. NDVI values were above the threshold (≥ 0.09) during the time when hoppers hatched and became adults (Figure GG(E)). The final step involved migration of swarms, which involved testing how long vegetation available on a ground could sustain a swarm and swarms following prevailing winds. An example of a swarm migration pathway together with landing sites and days stayed is shown in Figure GG(F).

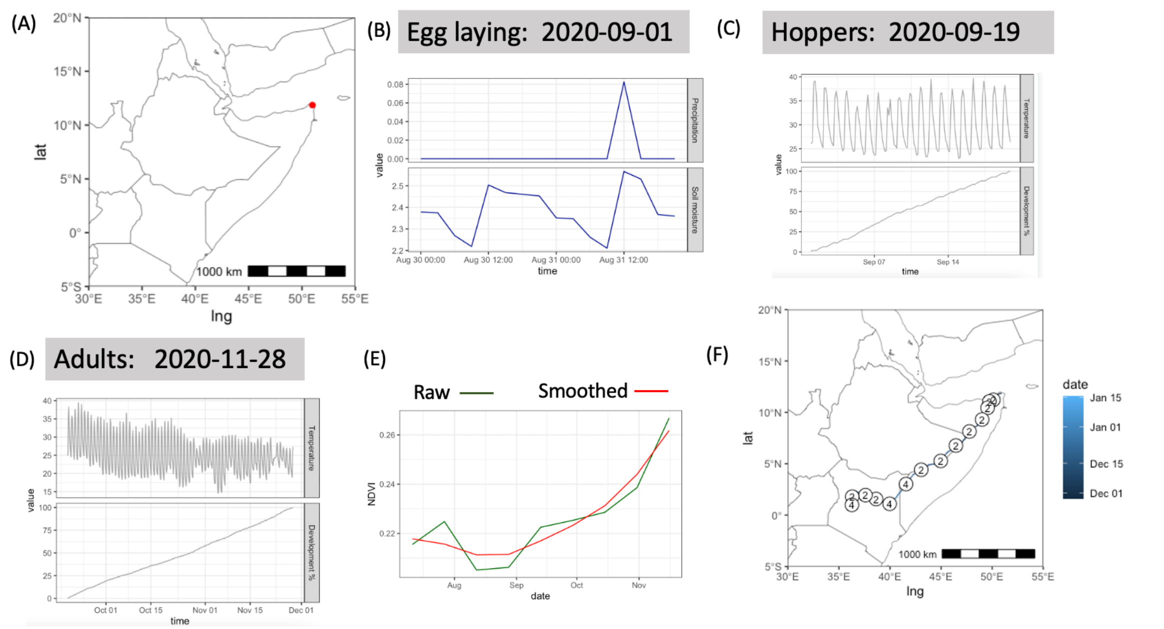

Figure GG. *Model simulations. (A) Breeding location. (B) Precipitation and soil moisture up to 48 hours before egg laying. (C) Temperature profile and change in development from egg to hopper. (D) Temperature profile and change in development from hopper to adult. (E) Raw and smoothed NDVI profile from July to December 2020. (F) Swarm movement pathway. Circles indicate landing sites, and numbers show how long swarms stayed. The base layer of the map is sourced from Natural Earth (https://www.naturalearthdata.com).*

Code is available in file '12 Tutorial.Rmd'.

S11.2. Model simulation from an extended breeding area in north east Somalia

We simulated breeding, hopper and swarm emergence in Somalia during September 2020 and assessed whether simulations of subsequent swarm migration and feeding were consistent with the reported observations of swarms arriving in Kenya, between four and five months later. We simulated locust breeding, development, feeding and dispersal using historic weather data from 15th September 2020 until 15th January 2021. The selected breeding area is indicated by a red square in Figure HH. Simulations that failed to satisfy conditions for development from emergence to adults and simulations for which sampled trajectories terminated in permanent water bodies, were deemed unsuccessful and removed. We ran simulations, until 25 successful breeding, development and migration histories were obtained. The simulations showed that DL hatched, developed from hoppers into adults and started migration before November 2020. We found that from mid December 2020 to mid January 2021 the simulated swarms would have reached northeast Kenya (Figure HH).

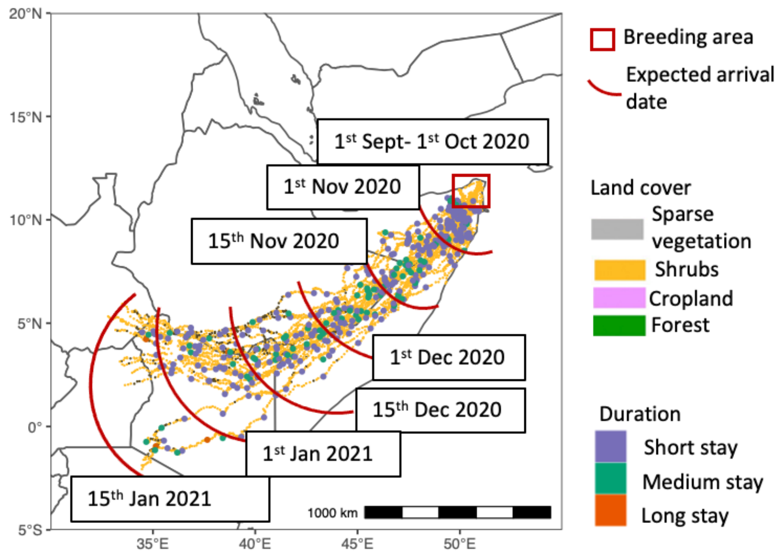

Figure HH. *Simulated expected arrival dates, type of land cover, landing sites and stay duration for desert locust swarms spreading from a breeding area in northern Somalia in Sept 2020. The base layer of the map is sourced from Natural Earth (https://www.naturalearthdata.com).*

S11.3. Model simulation from an extended region in northern Somalia

We chose the area shown in grey in Figure II(A) as the starting location for simulations, i.e. a large potential breeding area. We assumed that the time period for egg laying was between 1st September and 1st October 2020. We ran simulations, until 1,000 successful breeding, development and migration histories were obtained. The potential migration trajectories are shown in Figure II(B). We used these trajectories to calculate two statistics: the expected date of arrival (Figure II(C)) and the proportion of trajectories that visited a location (Figure II(D)). The expected date of arrival was calculated as the median of the dates when trajectories visited a location.

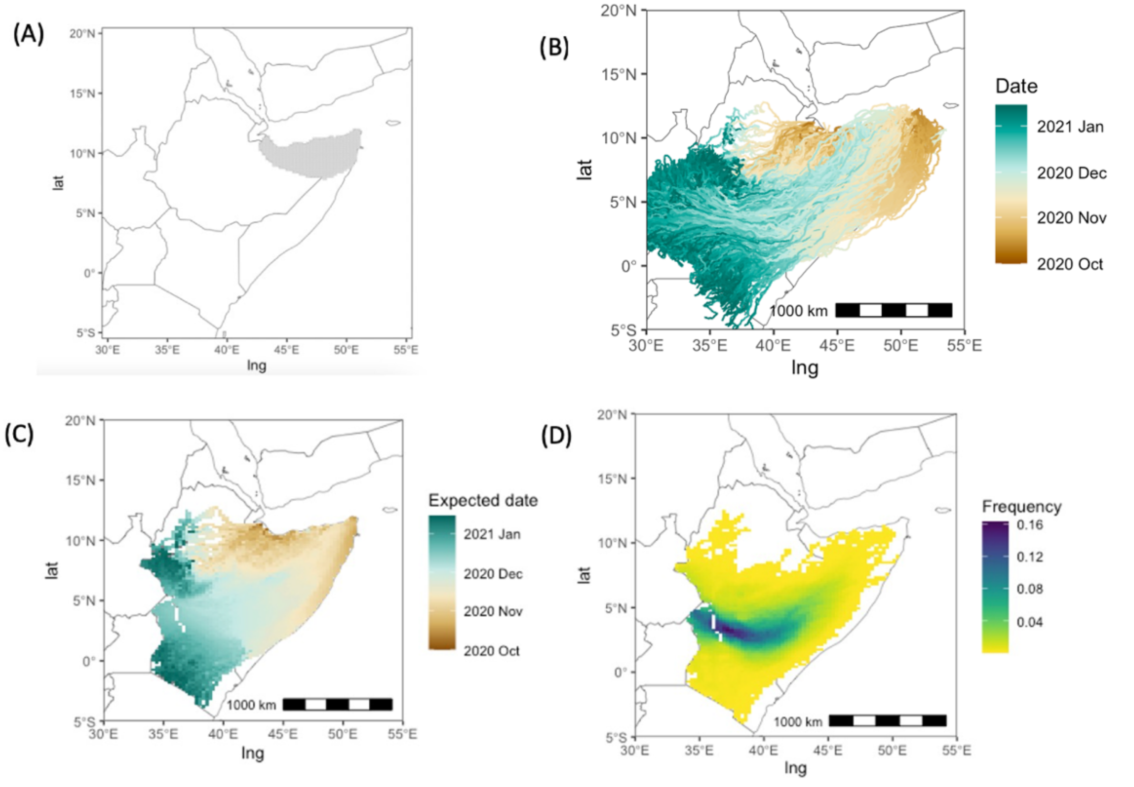

Figure II. *(A) Sites where model simulations started on 1st of September. (B) Potential migration pathways. (C) Distribution of expected arrival dates. (D) Distribution of fraction of simulations reaching grid cell. The base layer of the map is sourced from Natural Earth (https://www.naturalearthdata.com).*

S12. Tutorial

A separate tutorial to run the models is available at Zenodo. This requires R and Rstudio be installed on your machine.

The tutorial can be run by:

- Executing inline code. Make sure to execute the code in the order it appears in the tutorial.
- Rendering the .Rmd file into a R Markdown's supported format, as pdf or doc.

Code is available in file '12 Tutorial.Rmd'.

Bibliography

[1] Standard Operating Procedures (SOP) Desert Locust Biology and Behaviour. https://www.fao.org/ag/locusts/common/ecg/359/en/SOPBiologyENv2021.pdf

[2] Desert Locust briefs 2020, https://www.fao.org/ag/locusts/en/archives/briefs/2515/2516/index.html

[3] Desert Locust briefs 2021, https://www.fao.org/ag/locusts/en/archives/briefs/2515/2568/index.html

[4] SRTM 90m DEM Digital Elevation Database. https://srtm.csi.cgiar.org

[5] ISRIC - World Soil Information's Webdav. https://files.isric.org/soilgrids/former/2017-03-10/data/

[6] ISRIC - World Soil Information's Webdav. https://files.isric.org/public/afsis250m/

[7] Copernicus Global Land Service. https://zenodo.org/record/3939050

[8] Land Cover 100m: collection 3: epoch 2019: Globe. https://zenodo.org/record/3939050

[9] Copernicus Global Land Service. https://zenodo.org/record/3939050

[10] MOD13Q1 v061 MODIS/Terra Vegetation Indices 16-Day L3 Global 250 m SIN Grid https://doi.org/10.5067/MODIS/MOD13Q1.061

[11] Jones A, Thomson D, Hort M, Devenish B. The U.K. Met Office's Next-Generation Atmospheric Dispersion Model, NAME III. In: Air Pollution Modeling and Its Application XVII. Springer US; 2007. p. 580–589. Available from: https://doi.org/10.1007/978-0-387-68854-1_62.

[12] Unified Model https://www.metoffice.gov.uk/research/approach/modelling-systems/unified-model

[13] Frasso G, Eilers PH. L- and V-curves for optimal smoothing. Statistical Modelling. 2014;15(1):91–111. doi:10.1177/1471082x14549288.

[14] Eilers PHC, Pesendorfer V, Bonifacio R. Automatic smoothing of remote sensing data. In: 2017 9th International Workshop on the Analysis of Multitemporal Remote Sensing Images (MultiTemp). IEEE; 2017. p. 1–3. Available from: https://doi.org/10.1109/multi-temp.2017.8076705.

[15] Eilers PHC, Pesendorfer V, Bonifacio R. Automatic smoothing of remote sensing data. In: 2017 9th International Workshop on the Analysis of Multitemporal Remote Sensing Images (MultiTemp). IEEE; 2017. p. 1–3. Available from: https://doi.org/10.1109/multi-temp.2017.8076705.

[16] Muggeo VMR. Interval estimation for the breakpoint in segmented regression: a smoothed score-based approach. Australian &amp New Zealand Journal of Statistics. 2017;59(3):311–322. doi:10.1111/anzs.12200

[17] findPeaks https://github.com/stas-g/findPeaks

[18] Kimathi E, Tonnang HEZ, Subramanian S, Cressman K, Abdel-Rahman EM, Tesfayohannes M, et al. Prediction of breeding regions for the desert locust Schistocerca gregaria in East Africa. Scientific Reports. 2020;10(1). doi:10.1038/s41598-020-68895-2.

[19] Gorelick N, Hancher M, Dixon M, Ilyushchenko S, Thau D, Moore R. Google Earth Engine: Planetary-scale geospatial analysis for everyone. Remote Sensing of Environment. 2017;doi:10.1016/j.rse.2017.06.031.

[20] Oshiro TM, Perez PS, Baranauskas JA. How Many Trees in a Random Forest? In: Machine Learning and Data Mining in Pattern Recognition. Springer Berlin Heidelberg; 2012. p. 154–168. Available from: https://doi.org/10.1007/978-3-642-31537-4_13

[21] Freeman EA, Moisen G. PresenceAbsence: AnRPackage for Presence Absence Analysis. Journal of Statistical Software. 2008;23(11). doi:10.18637/jss.v023.i11.

[22] Kimathi E, Tonnang HEZ, Subramanian S, Cressman K, Abdel-Rahman EM, Tesfayohannes M, et al. Prediction of breeding regions for the desert locust Schistocerca gregaria in East Africa. Scientific Reports. 2020;10(1). doi:10.1038/s41598-020-68895-2.

[23] Desert Locust briefs 2020 https://www.fao.org/ag/locusts/en/archives/briefs/2515/2516/index.html

[24] Walters D, Baran AJ, Boutle I, Brooks M, Earnshaw P, Edwards J, et al. The Met Office Unified Model Global Atmosphere 7.0/7.1 and JULES Global Land 7.0 configurations. Geoscientific Model Development. 2019;12(5):1909–1963. doi:10.5194/gmd-12-1909-2019.

[25] Pedgley, D. 1981. Desert Locust Forecasting Manual. London: Centre for Overseas Pest Research

[26] Symmons PM, Cressman K. Desert Locust Guidelines, Biology and behaviour; 2001.

[27] Despland E, Rosenberg J, Simpson SJ. Landscape structure and locust swarming: a satellite's eye view. Ecography. 2004;27(3):381–391. doi:10.1111/j.0906-7590.2004.03779.x.

[28] Somprasong, K., Hutayanon, T., & Jaroonpattanapong, P. (2023). Using carbon sequestration as a remote-monitoring approach for reclamation's effectiveness in the open pit coal mine: a case study of Mae Moh, Thailand. *Energies*, *17*(1), 231.

[29] Bullen FT. Locusts and Grasshoppers as Pests of Crops and Pasture-A Preliminary Economic Approach. The Journal of Applied Ecology. 1966;3(1):147. doi:10.2307/2401671

[30] Bullen FT. Locusts and Grasshoppers as Pests of Crops and Pasture-A Preliminary Economic Approach. The Journal of Applied Ecology. 1966;3(1):147. doi:10.2307/2401671

[31] Rainey, R.C., 1963. Meteorology and the migration of desert locusts. Applications of synoptic meteorology in locust control.

[32] Malik AD, Nasrudin A, Parikesit, Withaningsih S. Vegetation Stands Biomass and Carbon Stock Estimation using NDVI - Landsat 8 Imagery in Mixed Garden of Rancakalong, Sumedang, Indonesia. IOP Conference Series: Earth and Environmental Science. 2023;1211(1):012015. doi:10.1088/1755-1315/1211/1/012015.

[33] https://www.asb.cgiar.org/PDFwebdocs/MeasuringCarbonstockacrosslandusesystems.pdf

[34] Bindu G, Rajan P, Jishnu ES, Ajith Joseph K. Carbon stock assessment of mangroves using remote sensing and geographic information system. The Egyptian Journal of Remote Sensing and Space Science. 2020;23(1):1–9. doi:10.1016/j.ejrs.2018.04.006

[35] Cabrera-Bosquet, Llorenç, G. Molero, A. N. N. A. Stellacci, J. Bort, S. Nogués, and J. Araus. "NDVI as a potential tool for predicting biomass, plant nitrogen content and growth in wheat genotypes subjected to different water and nitrogen conditions." Cereal Research Communications 39, no. 1 (2011): 147-159.

[36] GD, Bremm C, Bredemeier C, de Lima Menezes J, Alves LA, Tiecher T, et al. Normalized Difference Vegetation Index (NDVI) for soybean biomass and nutrient uptake estimation in response to production systems and fertilization strategies. Frontiers in Sustainable Food Systems. 2023;6. doi:10.3389/fsufs.2022.959681.

[37] Aranha J, Enes T, Calv˜ao A, Viana H. Shrub Biomass Estimates in Former Burnt Areas Using Sentinel 2 Images Processing and Classification. Forests. 2020;11(5):555. doi:10.3390/f11050555

[38] Filella I. Reflectance assessment of seasonal and annual changes in biomass and CO2 uptake of a Mediterranean shrubland submitted to experimental warming and drought. Remote Sensing of Environment. 2004;90(3):308–318. doi:10.1016/j.rse.2004.01.010

[39] Nakano T, Bavuudorj G, Urianhai NG, Shinoda M. Monitoring aboveground biomass in semiarid grasslands using MODIS images. Journal of Agricultural Meteorology. 2013;69(1):33–39. doi:10.2480/agrmet.69.1.1

[40] Farias GD, Bremm C, Bredemeier C, de Lima Menezes J, Alves LA, Tiecher T, et al. Normalized Difference Vegetation Index (NDVI) for soybean biomass and nutrient uptake estimation in response to production systems and fertilization strategies. Frontiers in Sustainable Food Systems. 2023;6. doi:10.3389/fsufs.2022.959681

[41] Zaitunah A, Samsuri, Ahmad AG, Safitri RA. Normalized difference vegetation index (ndvi) analysis for land cover types using landsat 8 oli in besitang watershed, Indonesia. IOP Conference Series: Earth and Environmental Science. 2018;126:012112. doi:10.1088/1755-1315/126/1/012112

[42] Malik AD, Nasrudin A, Parikesit, Withaningsih S. Vegetation Stands Biomass and Carbon Stock Estimation using NDVI - Landsat 8 Imagery in Mixed Garden of Rancakalong, Sumedang, Indonesia. IOP Conference Series: Earth and Environmental Science. 2023;1211(1):012015. doi:10.1088/1755-1315/1211/1/012015.

[43] Bindu G, Rajan P, Jishnu ES, Ajith Joseph K. Carbon stock assessment of mangroves using remote sensing and geographic information system. The Egyptian Journal of Remote Sensing and Space Science. 2020;23(1):1–9. doi:10.1016/j.ejrs.2018.04.006

[44] Farias, G. D., Bremm, C., Bredemeier, C., de Lima Menezes, J., Alves, L. A., Tiecher, T., ... & de Faccio Carvalho, P. C. (2023). Normalized Difference Vegetation Index (NDVI) for soybean biomass and nutrient uptake estimation in response to production systems and fertilization strategies. *Frontiers in Sustainable Food Systems*, *6*, 959681.

[45] Zaitunah A, Samsuri, Ahmad AG, Safitri RA. Normalized difference vegetation index (ndvi) analysis for land cover types using landsat 8 oli in besitang watershed, Indonesia. IOP Conference Series: Earth and Environmental Science. 2018;126:012112. doi:10.1088/1755-1315/126/1/012112

[46] Retkute R, Thurston W, Gilligan CA. Bayesian Inference for Multiple Datasets. Stats. 2024;7(2):434–444. doi:10.3390/stats7020026.

[47] Allen-Sader C, Thurston W, Meyer M, Nure E, Bacha N, Alemayehu Y, et al. An early warning system to predict and mitigate wheat rust diseases in Ethiopia. Environmental Research Letters. 2019;14(11):115004. doi:10.1088/1748-9326/ab4034
